# Surgical Results and Complications for Open, Laparoscopic, and Robot-assisted Radical Prostatectomy: A Reverse Systematic Review

**DOI:** 10.1016/j.euros.2022.08.015

**Published:** 2022-09-08

**Authors:** Tomás Bernardo Costa Moretti, Luís Alberto Magna, Leonardo Oliveira Reis

**Affiliations:** a Doctoral Program in Medical Pathophysiology and UroScience, Division of Urology, Faculty of Medical Sciences, State University of Campinas, Campinas, Brazil; b Department of Medical Genetics, State University of Campinas, Campinas, Brazil; c Center for Life Sciences, Pontifical Catholic University of Campinas, Campinas, Brazil

**Keywords:** Laparoscopic surgery, Open surgery, Robot-assisted surgery, Radical prostatectomy, Methodology, Reverse systematic review, Complications

## Abstract

**Context:**

The advantages of minimally invasive surgery for radical prostatectomy (RP) have been demonstrated in a number of systematic reviews (SRs). However, the rigorous study selection process for SR means that a lot of information can be excluded, leading to a very specific clinical scenario that is often unrepresentative of real life. Our new reverse SR methodology generates a heterogeneous population database that covers a wide range of clinical scenarios.

**Objective:**

To compare perioperative surgical results and complications for open retropubic RP (RRP), laparoscopic RP (LRP), and robot-assisted RP (RARP) in a reverse SR.

**Evidence acquisition:**

Eight databases were searched for SRs on RRP, LRP, or RARP between 2000 and 2020 (80 SRs). All references used in these SRs were captured for analysis (1724 articles). Perioperative outcomes and complications were compared among the RRP, LRP, and RARP approaches.

**Evidence synthesis:**

We identified 559 (32.4%) reports on RRP, 413 (23.9%) on LRP, and 752 (43.7%) on RARP, involving 1 353 485 patients overall. RARP showed a significantly higher annual volume of surgery per surgeon (AVSS) in comparison to RRP and LRP (mean 64.29, 43.26, and 41.47, respectively), a higher percentage of low-risk patients (prostate-specific antigen <10 ng/ml, Gleason <7, stage <cT2), and a lower rate of lymphadenectomy, culminating in a lower complication rate (12.3% for RARP, 16.3% for LRP, 20.2% for RRP). Among all outcomes, only AVSS was significantly correlated with complication rates. An AVSS of 30, 95 and 95 surgeries/yr was required for RARP, LRP, and RRP, respectively, to obtain a complication rate of 12.3% (average for RARP). RARP showed better performance for all perioperative variables studied except for operative time (operative time: 199.8 vs 214.9 vs 169.5 min; estimated blood loss: 228.2 vs 408.0 vs 852.1 ml; blood transfusion rate: 2.8% vs 6.5% vs 19.8%; length of stay: 2.9 vs 5.7 vs 6.1 d; catheter time: 7.8 vs 8.5 vs 11.0 d for RARP vs LRP vs RRP).

**Conclusions:**

Our reverse SR involved a wide real-life representative sample and reference values established in the literature and revealed that minimally invasive surgery had the best perioperative and complication results, especially RARP, which was associated with less complex cases, higher annual surgeon volume, and greater performance.

**Patient summary:**

We used a wide sample representative of real-life surgical practice and reference values established in the literature for three techniques for removal of the prostate to guide patients and physicians in deciding the best surgical treatment for prostate cancer according to availability.

## Take Home Message

Our review revealed a significantly higher annual volume of surgery per surgeon (AVSS), a higher percentage of low-risk patients, and lower rates of lymphadenectomy and complications for robot-assisted radical prostatectomy (RARP) compared to open and laparoscopic techniques. RARP showed better performance for all the perioperative variables studied except for operative time. Among all the outcomes, only AVSS was significantly correlated with complication rates. The AVSS required to achieve a target complication rate was significantly lower for RARP.

## 1 Introduction

The advantages of minimally invasive surgery in the surgical treatment of prostatic carcinoma are well known and the European Association of Urology and American Urological Association guidelines recognize these advantages and recommend the minimally invasive route owing to better perioperative results in terms of bleeding and transfusion rates, length of hospital stay, and complications [1], [2].

During the contemporary history of radical prostatectomy (RP), the three main techniques—retropubic RP (RRP), laparoscopic RP (LRP), and robot-assisted (RARP)—have been compared in several studies with different levels of evidence, ranging from expert opinions to systematic reviews (SRs) [3].

SR with meta-analysis is an excellent tool for bringing together methodologically similar studies in order to increase the number of patients and thus the statistical strength of comparisons. However, during the process of choosing these studies, a lot of information can be excluded, leading to a very specific clinical scenario that is often unrepresentative of real life [4].

Thus, our study group designed a new SR methodology called reverse SR (RSR) to compare the three RP techniques [5] and to generate a heterogeneous population database that covers several different scenarios. Here we used RSR to understand how perioperative variables and complication rates have evolved over the 20 yr for which the three techniques have coexisted and to explore correlation with possible bias and confounding that may have influenced the results and trends.

## 2 Evidence acquisition

### 2.1 Description of the methodology

In classic SR, a systematic search is performed in databases to locate original clinical studies that answered a specific question. After this search, studies that are homogeneous and comparable—that is, studies that used the same methods, populations, and outcomes—are selected for inclusion and can be merged for statistical analysis, called a meta-analysis [6], [7].

In the case of RSR, the opposite path is followed. The literature search is carried out with the objective of identifying all SRs in the history of the technique under study, regardless of the question of interest, and gathering as many of these studies possible to generate a heterogeneous population with complete information for the outcomes that most interested the research community in that area. At this stage, when gathering all the SRs, the main focus is to capture all the studies included in these reviews that were used to answer the investigators’ questions (Supplementary material).

### 2.2 Search methodology and study design

**Fig. 1:**
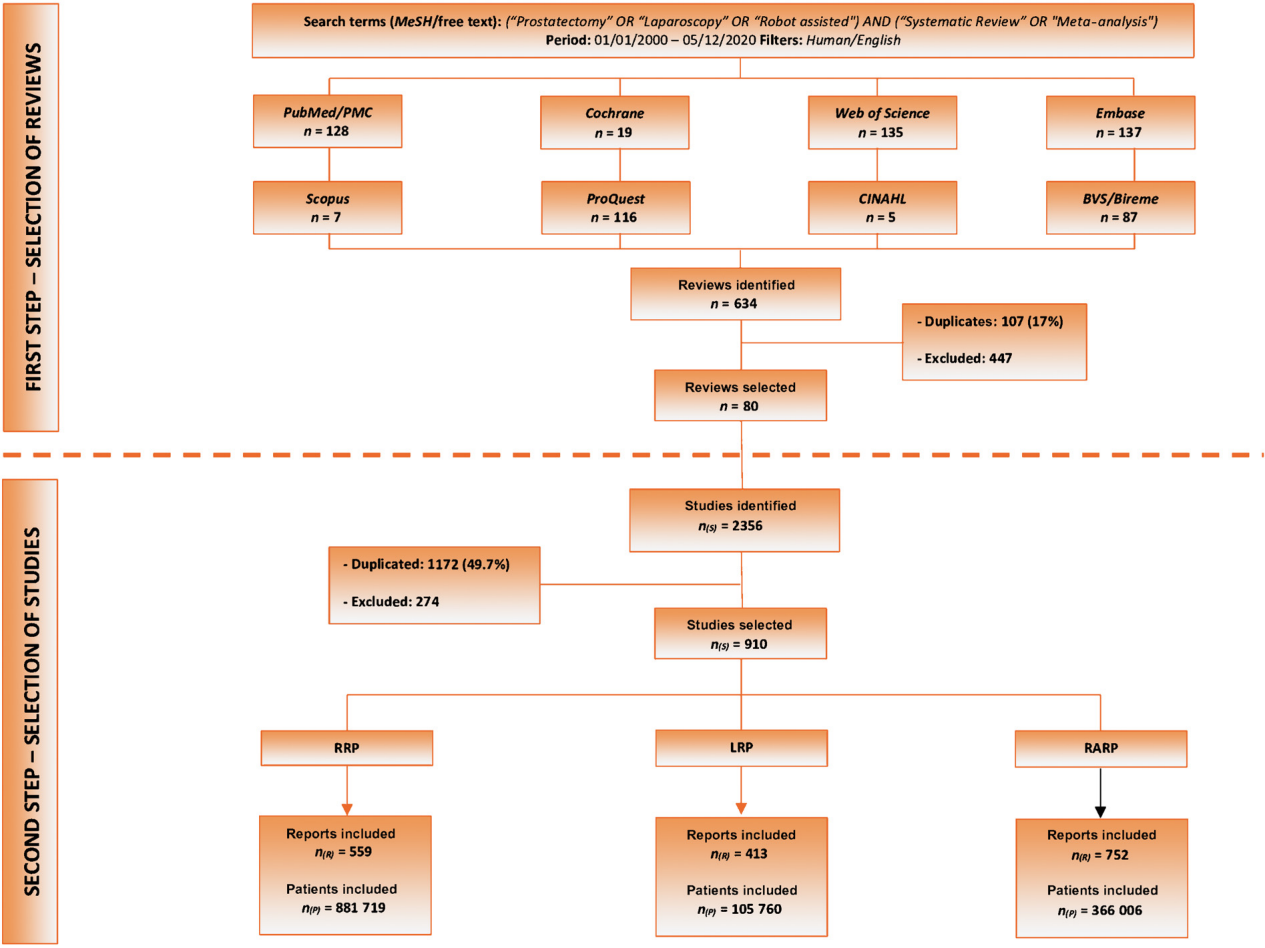
Study design showing the two phases of the reverse systematic review. The first phase involves the selection of classic systematic reviews from the literature. The second phase involves selection of primary studies used in the reviews selected in the first phase. RRP = open radical prostatectomy; LRP = laparoscopic radical prostatectomy; RARP = robot-assisted radical prostatectomy.

In December 2020, a literature search was carried out using eight databases: PubMed, Web of Science, Cochrane Library, Embase, ProQuest, CINAHL (The Cumulative Index to Nursing and Allied Health Literature), VHL/Bireme, and Scopus (Fig. 1). We searched for SRs, with or without meta-analysis, that addressed the techniques of RRP, LRP, and RARP, with a general strategy based on health descriptors and synonyms referring to the terms “Laparoscopy”, “Open”, “Retropubic”, “Prostatectomy”, “Robotic Surgical Procedures”, “Systematic Review”, and “Meta-analysis” in the “Title, Abstract and Subject” fields. We then applied the limiters “humans”, gender (“male”), language (“English”), and type of study (“Systematic Review”). The period in the literature was between January 1, 2000 and December 5, 2020. For each database, and adaptation of the search methodology necessary was carried out (Supplementary material).

After the reviews were identified in the initial search, two researchers (T.B.C.M. and L.O.R.) independently selected reviews that included at least one of the three RP techniques. After the initial screening, the full texts were analyzed and any discrepancies were resolved after open discussion between the authors. Reviews without systematization of the search or integrative methodology, conference and congress abstracts, and papers on other techniques were excluded.

Owing to the difficulty in standardizing health descriptors (MeSH terms) for the databases and classifying a study as an SR, we included studies that, despite not mentioning if the PRISMA criteria [4] were followed in their methodology, provided a clear description of the systematization of the search criteria.

Once all the SRs were chosen, the next step was to extract all the articles cited in the bibliographic references that were included in these SRs for analysis. Publications that were abstracts, meeting reports, or congress proceedings were excluded. As before, two researchers separately reviewed the studies (T.B.C.M. and L.O.R.) and discrepancies in selection were resolved via open discussion.

After the sample was chosen via the systematized method described, all the studies were analyzed by the main author (T.B.C.M.) and the largest amount of data available was captured and tabulated in a dedicated Excel spreadsheet.

When a study evaluated more than one cohort, each one was considered as an isolated study and was called a *report*, which is the unit of publication used in the study.

The global content from all of the studies selected, including bibliographic, demographic, and clinicosurgical variables, was used to generate a reference population database for various studies and applications.

### 2.3 Variables analyzed and comparative methods

For this study, perioperative variables separated into the three groups (RRP, LRP, and RARP) were analyzed, including: number of patients, annual volume of surgeries per surgeon (AVSS), age (yr), body mass index (kg/m^2^), initial prostate-specific antigen (PSA; mg/dl), Gleason score (mean and stratified), clinical T stage, operative time (min), estimated blood loss (EBL; ml), blood transfusion rate (%), length of hospital stay (d), bladder catheterization time (d), and overall and stratified perioperative complication rates (minor, major, and Clavien-Dindo grade I–V [8]).

The mean values for these variables were computed to calculate population reference values. A temporal analysis of the variable means was performed by dividing the reports into four periods in relation to year of publication: first period, before 2005; second period, 2006–2010; third period, 2011–2015; and fourth period, after 2015). In addition, a correlation analysis of the variables was performed to identify factors related to the complication rate.

### 2.4 Statistical analysis

The measure of central tendency was represented by the mean, and dispersion by the standard error of the mean. Comparisons between means were performed using the parametric analysis of variance (ANOVA) test, with multiple variables analyzed according to the homogeneity of the variance, as defined according to the Levene test (Tukey, Bonferroni, or Games-Howell correction). Correlation analyses of continuous variables were performed using Spearman’s correlation. The regression curve was adjusted using the rational regression model (nonlinear). The significance level was set at *p* < 0.05 (two-tailed). Statistical analyses were performed in SPSS v.24. Curve Express Professional v2.7 was used for regression graphs and adjustments.

## 3 Results

The first stage of the systematic search for SRs on RP identified 634 studies in eight databases. After excluding 107 duplicates (17%) and 447 studies that did not meet the inclusion criteria, 80 SRs were included in the second stage (Supplementary material).

In the second stage, all selected SRs were read and the primary studies used were captured, resulting in a total of 2356 citations. After excluding 1172 (49.7%) duplicates and 274 studies that did not meet the inclusion criteria, 910 studies were selected for the global database (Supplementary material). Owing to the existence of more than one cohort in some studies, each cohort was considered separately, resulting in 1724 publication units or reports. Separated by technique, 559 (32.4%) reports on RRP, 413 (23.9%) reports on LRP, and 752 (43.7%) reports on RARP were included (Fig. 1).

Descriptive and comparative statistics for preoperative clinical characteristics for the three technique groups are listed in Table 1.

**Table 1:** Descriptive statistics for clinical variables by surgical technique and univariate comparative analysis of mean values

Parameter	A: Open RP				B: Laparoscopic RP				C: Robot-assisted RP				Analysis of variance	
	*N*_r_	*N*_p_	Mean	SE	*N*_r_	*N*_p_	Mean	SE	*N*_r_	*N*_p_	Mean	SE	*p* value	Multiple comparison†
Patients (*n*)	559	881 719	1577	309	413	105 760	256	24	752	366 006	487	122	<0.001	AB/AC^c^
AVSS	270	151 656	43.26	3.53	266	64 331	41.47	2.53	504	111 900	64.29	4.14	<0.001	AC/BC^c^
Age (yr)	448	545 521	62.78	0.16	381	90 929	62.91	0.15	664	312 188	61.42	0.12	<0.001	AC/BC^c^
BMI (kg/m^2^)	121	43 979	26.18	0.17	179	39 270	26.32	0.14	463	111 753	27.00	0.09	<0.001	AC/BC^a^
PSA (mg/dl)	330	157 184	8.91	0.26	358	79 110	8.75	0.17	599	143 034	7.71	0.19	<0.001	AC/BC^a^
PSA <4 mg/dl (%)	50	53 710	17.55	1.27	24	7245	16.88	3.34	34	12 923	20.48	1.19	0.347	N.S.
PSA 4–10 mg/dl (%)	53	55 496	57.68	1.43	31	9764	60.34	2.28	34	12 923	65.69	1.18	0.02	AC^c^
PSA 10–20 mg/dl (%)	45	75 686	20.00	1.11	33	11 359	24.53	1.71	21	89 774	13.96	1.70	<0.001	AC/BC
PSA >20 mg/dl (%)	27	59 422	8.54	0.97	16	8850	14.23	4.20	12	82 008	5.17	1.54	0.057	N.S.
cGS (mean)	79	16 377	6.09	0.06	148	23 467	6.17	0.04	133	22 938	6.42	0.03	<0.001	AC/BC^c^
cGS <7 (%)	178	143 018	55.92	1.50	144	35 049	58.39	1.59	342	175 112	53.25	1.10	0.029	BC^a^
cGS 7 (%)	165	137 255	34.23	1.25	131	33 592	33.33	1.19	322	172 979	35.56	0.80	0.304	N.S.
cGS >7 (%)	167	143 749	11.05	0.93	125	32 502	9.19	0.89	318	178 675	12.56	0.80	0.042	BC^a^
Stage cT1 (%)	225	174 785	58.66	1.36	211	44 589	57.23	1.64	353	172 436	68.76	1.01	<0.001	AC/BC^c^
Stage cT1a (%)	27	20 924	5.70	2.32	28	6274	5.71	2.54	21	8340	0.65	0.28	0.203	N.S.
Stage cT1b (%)	28	23 280	7.83	2.86	32	7825	7.83	3.30	22	6974	1.11	0.28	0.192	N.S.
Stage cT1c (%)	131	90 360	58.63	1.93	140	28 402	57.30	1.94	210	45 774	71.30	1.21	<0.001	AC/BC^c^
Stage cT2 (%)	212	165 917	38.70	1.28	205	39 521	41.16	1.39	326	161 285	31.76	1.07	<0.001	AC/BC^a^
Stage cT2a (%)	83	77 357	26.22	1.41	102	18 037	25.41	1.11	144	34 059	19.37	0.89	<0.001	AC/BC^a^
Stage cT2b (%)	71	70 035	15.94	1.56	93	16 956	10.83	1.09	117	29 388	8.11	0.81	<0.001	AB/AC^c^
Stage cT2c (%)	42	34 184	9.57	1.32	61	12 482	10.60	1.56	62	17 404	6.67	0.96	0.073	N.S.
Stage cT3 (%)	118	110 734	8.04	1.24	99	24 971	9.67	1.14	184	142 019	5.91	0.69	0.022	BC^c^
Stage cT3a (%)	24	13 859	10.38	3.61	43	8222	11.80	1.55	42	11 360	8.38	1.39	0.412	N.S.
Stage cT3b (%)	13	10 393	3.24	1.22	23	5466	4.52	1.01	30	9675	3.26	0.68	0.519	N.S.
Stage cT4 (%)	8	7 836	1.54	0.69	4	2689	1.13	0.76	11	7489	0.71	0.32	0.503	N.S.

*N*_r_ = number of reports; *N*_p_ = number of patients; AVSS = annual volume of surgeries per surgeon; BMI = body mass index; PSA = prostate-specific antigen; cGS = clinical Gleason score; RP = radical prostatectomy; SE = standard error of the mean; N.S. = not significant (at two-tailed *p* > 0.05).

† Multiple comparison tests among groups A (open RP), B (laparoscopic RP), and C (robot-assisted RP) with appropriate correction: a, Tukey; b, Bonferroni; or c, Games-Howell correction.

The mean number of patients was significantly higher for the RRP studies (*n* = 1577) than for the other two techniques. The higher number of patients in a shorter time led to a higher mean AVSS for RARP (64.29) than for RRP (43.26) and LRP (41.47). For the initial staging variables (PSA, Gleason score, and clinical T stage), there were significant differences between the RARP group and the other two techniques, with a higher percentage of low-risk patients (PSA <10 ng/ml, Gleason <7, stage <cT2) undergoing RARP, and a similar profile between RRP and LRP (Table 1).

**Fig. 2:**
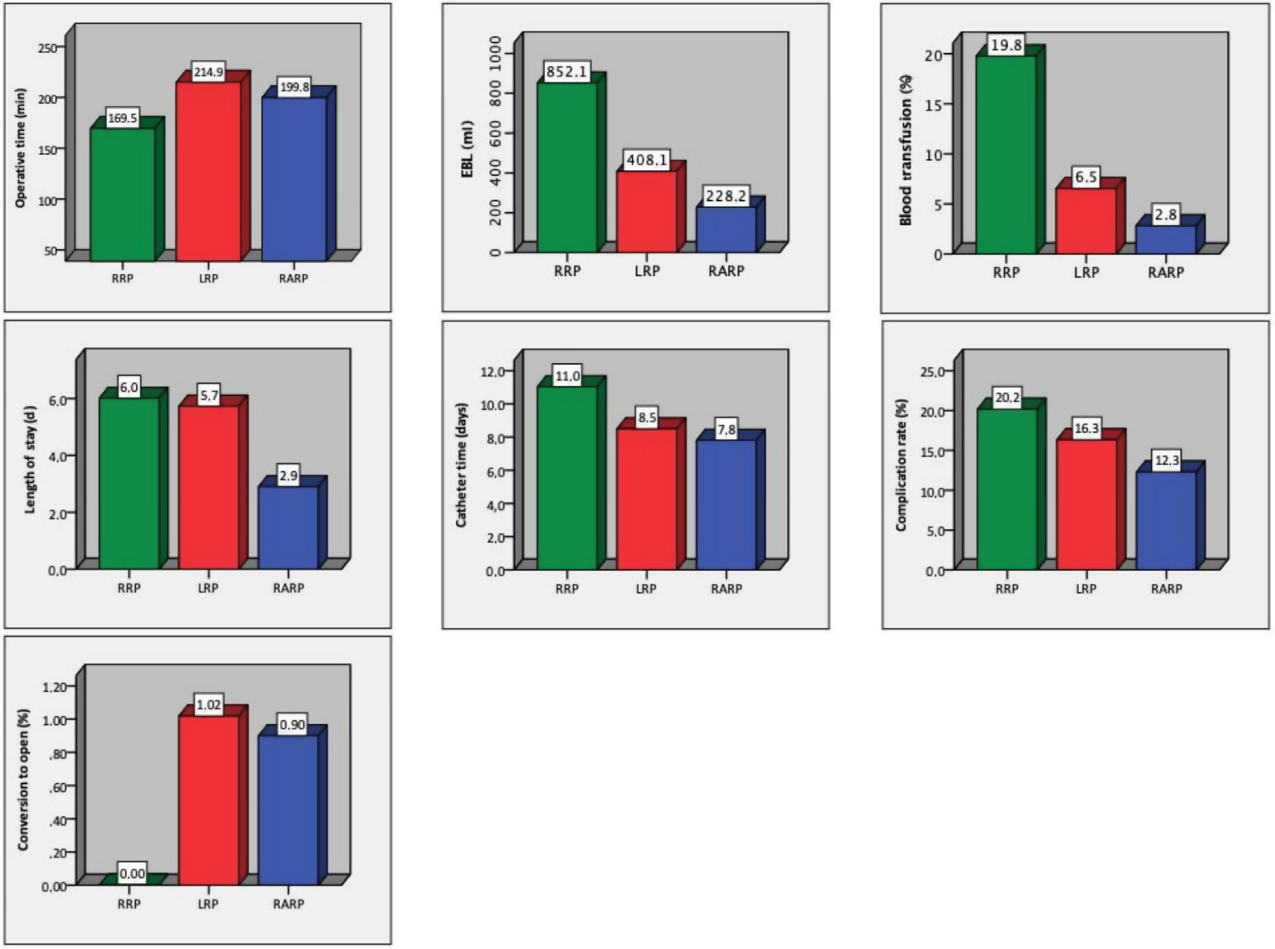
Comparison of the mean values for perioperative variables among the three techniques. RRP = open radical prostatectomy; LRP = laparoscopic radical prostatectomy; RARP = robot-assisted radical prostatectomy; EBL = estimated blood loss.

Descriptive and comparative statistics for perioperative variables for the three groups are listed in Table 2. Analysis of perioperative variables revealed statistically significant differences among the three techniques for almost all the comparisons, as visualized in Figure 2. Robotic surgery showed better performance for all of the variables studied, except for operative time, which was shortest with the open approach.

**Table 2:** Descriptive statistics for perioperative variables by surgical technique and univariate comparative analysis of mean values

Parameter	A: Open RP				B: Laparoscopic RP				C: Robot-assisted RP				Analysis of variance	
	*N*_r_	*N*_p_	Mean	SE	*N*_r_	*N*_p_	Mean	SE	*N*_r_	*N*_p_	Mean	SE	*p* value	Multiple comparison†
Operative time (min)	179	54 876	169.53	3.89	326	73 251	214.92	3.57	473	110 717	199.78	3.04	<0.001	AB/AC/BC^a^
Pelvic lymphadenectomy (%)	110	78 970	82.69	2.68	162	46 893	49.30	2.17	184	122 390	59.53	2.53	<0.001	AB/AC/BC^c^
Nerve-sparing rate (%)	128	62 116	67.05	2.49	185	43 744	56.65	1.96	240	67 212	80.57	1.24	<0.001	AB/AC/BC^c^
Unilateral nerve-sparing (%)	85	40 661	26.09	2.88	158	39 108	19.96	0.98	181	56 043	25.33	1.36	0.010	BC^c^
Bilateral nerve-sparing (%)	103	56 266	59.52	2.90	178	42 389	43.49	2.08	215	61 804	62.50	1.70	<0.001	AB/BC^c^
Estimated blood loss (ml)	194	45 141	852.11	29.95	260	46 361	408.05	14.09	454	104 747	228.18	6.22	<0.001	AB/AC/BC^c^
Blood transfusion (%)	157	347 781	19.77	1.49	228	54 389	6.55	0.55	243	143 225	2.83	0.32	<0.001	AB/AC/BC^c^
Conversion to open RP (%)	N.A.	N.A.	N.A.	N.A.	170	42 013	1.02	0.16	128	29 975	0.90	0.18	0.795	N.S.
Length of stay (d)	159	395 351	6.01	0.35	227	55 730	5.73	0.22	338	117 748	2.90	0.14	<0.001	AC/BC^c^
Catheter time (d)	90	18 007	11.03	0.51	218	41 204	8.50	0.21	246	54 800	7.81	0.17	<0.001	AB/AC/BC^c^
Complication rate (%)	148	368 848	20.17	1.38	243	62 387	16.33	0.76	282	148 237	12.30	0.52	<0.001	AB/AC/BC^c^
Minor complications (%)	26	10 759	20.37	3.34	109	30 049	12.70	1.10	109	33 494	10.08	0.67	<0.001	AC^c^
Major complications (%)	27	10 822	7.01	1.51	112	30 256	5.35	0.50	105	33 407	3.54	0.35	0.002	BC^c^
Clavien I complications (%)	13	7602	8.05	1.41	46	17 318	7.38	0.94	73	25 640	5.18	0.61	0.059	N.S.
Clavien II complications (%)	13	7602	17.71	5.02	48	17 989	6.33	0.72	70	25 903	4.47	0.41	<0.001	AB/AC^b^
Clavien IIIa complications (%)	13	5211	4.49	1.48	42	15 112	2.72	0.43	57	21 797	2.18	0.46	0.103	N.S.
Clavien IIIb complications (%)	12	5128	5.03	1.72	36	13 624	2.35	0.39	50	18 001	1.38	0.23	<0.001	AB/AC^b^
Clavien IVa complications (%)	13	4943	0.96	0.60	31	10 843	0.64	0.18	46	18 755	0.52	0.16	0.550	N.S.
Clavien IVb complications (%)	9	3532	0.00	0.00	21	7028	0.05	0.05	35	12 912	0.02	0.01	0.550	N.S.
Clavien V complications (%)	28	208 494	0.24	0.05	24	8402	0.05	0.03	39	30 565	0.04	0.03	<0.001	AB/AC^c^

*N*_r_ = number of reports; *N*_p_ = number of patients; SE = standard error of the mean; RP = radical prostatectomy; N.S. = not significant (two-tailed *p* > 0.05); N.A. = not applicable.

† Multiple comparison tests among groups A (open RP), B (laparoscopic RP), and C (robot-assisted RP) with appropriate corrections: a, Tukey; b, Bonferroni; or c, Games-Howell corrections.

Temporal analysis revealed that in the first period (before 2005), RRP and LRP were the techniques most used for patients with lower risk (PSA <10 ng/l, Gleason <7, stage T1–2), with better perioperative results obtained with LRP. For the second period there was a change in the case pattern for RARP, with a greater proportion of patients at low risk undergoing surgery via this approach and a gradual improvement in perioperative results over time until the fourth period, when discrepancies become less evident. Regardless of the period, the best perioperative results, except for operative time, were observed for RARP (Table 3).

**Table 3:** Descriptive statistics for perioperative variables by surgical technique and univariate comparative analysis of mean values stratified into four periods according to the year of publication of the studies

Parameter	1st period (before 2005)				2nd period (2006–2010)				3rd period (2011–2015)				4th period (after 2015)			
	Variable mean (*N*_r_; SE)			*p* value†	Variable mean (*N*_r_; SE)			*p* value†	Variable mean (*N*_r_; SE)			*p* value†	Variable mean (*N*_r_; SE)			*p* value†
	A: RRP	B: LRP	C: RARP		A: RRP	B: LRP	C: RARP		A: RRP	B: LRP	C: RARP		A: RRP	B: LRP	C: RARP	
Age (yr)	62.7 (115; 0.3)	62.6 (89; 0.3)	60.3 (35; 0.5)	0.001 AC/BC^a^	62.53 (246; 0.2)	62.35 (166; 0.2)	60.59 (337; 0.1)	<0.001 AC/BC^c^	63.48 (78; 0.3)	63.65 (109; 0.2)	62.20 (227; 0.2)	<0.001 AC/BC^a^	64.61 (9; 1.1)	65.13 (17; 0.7)	63.67 (65; 0.3)	0.138 N.S.
BMI (kg/m^2^)	25.4 (3; 1.6)	26.8 (31; 0.3)	27.4 (16; 0.2)	0.144 N.S.	26.57 (75; 0.2)	26.46 (65; 0.2)	27.39 (232; 0.1)	<0.001 AC/BC^a^	25.49 (39; 0.3)	26.11 (74; 0.2)	26.83 (166; 0.1)	<0.001 AC/BC^a^	26.10 (4; 0.6)	25.44 (9; 0.4)	25.63 (49; 0.2)	0.810 N.S.
iPSA (mg/dl)	9.2 (60; 0.5)	8.8 (87; 0.3)	7.7 (36; 0.3)	0.039 AC/BC^a^	8.94 (202; 0.4)	8.61 (157; 0.2)	7.02 (298; 0.2)	<0.001 AC/BC^c^	8.74 (60; 0.5)	8.51 (98; 0.3)	8.52 (208; 0.4)	0.947 N.S.	7.33 (8; 0.6)	11.31 (16; 1.9)	8.32 (57; 0.3)	0.027 N.S.
iPSA <4 mg/dl (%)	17.3 (23; 1.9)	12.2 (6; 2.3)	N.A.	0.208 N.S.	18.57 (23; 1.9)	20.20 (14; 5.6)	21.70 (29; 1.2)	0.640 N.S.	12.70 (3; 1.7)	12.35 (4; 0.2)	12.00 (4; 1.6)	0.933 N.S.	14.00 (1; N.A.)	N.A. N.A.	19.00 (1; N.A.)	N.A. N.A.
iPSA 4–10 mg/dl (%)	56.1 (26; 1.8)	58.5 (6; 2.2)	N.A.	0.546 N.S.	59.31 (23; 2.5)	61.04 (18; 3.5)	66.68 (29; 1.2)	0.048 AC^c^	55.23 (3; 3.4)	57.27 (6; 3.7)	58.40 (4; 4.1)	0.882 N.S.	69.00 (1; N.A.)	77.00 (1; N.A.)	66.00 (1; N.A.)	N.A. N.A.
iPSA 10–20 mg/dl (%)	20.6 (24; 1.3)	26.3 (7; 3.2)	N.A.	0.063 N.S.	18.96 (14; 2.1)	21.57 (17; 2.7)	10.43 (12; 1.1)	0.005 AC/BC^c^	24.23 (4; 6.2)	29.46 (8; 1.9)	24.50 (4; 5.5)	0.542 N.S.	14.80 (3; 1.7)	23.00 (1; N.A.)	13.98 (5; 2.3)	0.280 N.S.
iPSA >20 mg/dl (%)	8.2 (16; 1.1)	33.0 (2; 30.0)	N.A.	0.011 N.A.	9.33 (8; 2.5)	7.39 (7; 1.8)	1.28 (6; 0.2)	0.030 AC/BC^c^	9.15 (2; 1.8)	15.70 (7; 5.4)	12.30 (3; 2.3)	0.774 N.S.	7.40 (1; N.A.)	N.A. N.A.	5.80 (3; 2.4)	0.776 N.S.
cGS (mean)	5.8 (25; 0.1)	6.0 (50; 0.1)	6.4 (12; 0.2)	0.001 AC/BC^b^	6.17 (49; 0.1)	6.25 (76; 0.1)	6.36 (85; 0.1)	0.023 AC^b^	6.62 (5; 0.2)	6.22 (22; 0.1)	6.51 (32; 0.1)	0.083 N.S.	N.A. N.A.	N.A. N.A.	7.00 (1; N.A.)	N.A. N.A.
cGS <7 (%)	68.8 (33; 1.7)	71.3 (22; 2.7)	46.9 (14; 5.5)	<0.001 AC/BC^b^	56.87 (104; 2.0)	62.39 (48; 2.1)	60.54 (169; 1.3)	0.136 N.S.	43.22 (34; 3.1)	51.97 (61; 2.7)	47.86 (125; 1.8)	0.141 N.S.	42.49 (7; 6.3)	51.97 (13; 4.5)	39.50 (34; 2.8)	0.082 N.S.
cGS 7 (%)	24.9 (29; 1.2)	24.6 (16; 2.8)	35.2 (12; 5.2)	0.021 AC/BC^b^	34.14 (96; 1.1)	30.48 (47; 1.5)	31.05 (159; 0.9)	0.157 N.S.	40.16 (34; 2.2)	37.68 (57; 2.0)	38.58 (118; 1.4)	0.751 N.S.	47.22 (6; 5.8)	35.71 (11; 2.6)	46.59 (33; 2.5)	0.070 N.S.
cGS >7 (%)	6.1 (33; 0.9)	4.2 (14; 0.8)	11.6 (10; 2.7)	0.006 AC/BC^b^	10.47 (92; 1.1)	6.37 (51; 0.9)	9.65 (156; 1.1)	0.126 N.S.	16.25 (35; 2.7)	11.91 (49; 1.4)	15.86 (123; 1.4)	0.249 N.S.	16.04 (7; 5.1)	16.46 (11; 5.9)	14.52 (29; 1.4)	0.887 N.S.
Stage cT1 (%)	50.6 (64; 2.7)	56.2 (57; 3.1)	59.3 (16; 5.2)	0.235 N.S.	61.96 (122; 1.7)	58.91 (88; 2.4)	74.36 (187; 1.0)	<0.001 AC/BC^c^	62.37 (35; 2.9)	56.51 (60; 3.3)	62.86 (126; 1.9)	0.186 N.S.	55.23 (4; 6.8)	49.67 (6; 7.8)	62.37 (24; 4.2)	0.358 N.S.
Stage cT1a (%)	2.3 (14; 0.3)	1.3 (11; 0.5)	0.5 (3; 0.3)	0.059 N.S.	5.33 (11; 2.2)	5.38 (12; 3.5)	1.09 (10; 0.6)	0.437 N.S.	31.30 (2; 29.6)	16.12 (5; 11.0)	0.17 (6; 0.1)	0.188 N.S.	N.A. N.A.	N.A. N.A.	0.15 (1; N.A.)	N.A. N.A.
Stage cT1b (%)	4.8 (16; 0.8)	0.5 (12; 0.2)	2.0 (3; 1.5)	<0.001 BC^c^	9.37 (11; 6.6)	7.07 (13; 4.3)	0.71 (12; 0.2)	0.378 N.S.	39.10 (1; N.A.)	25.20 (6; 13.4)	1.43 (7; 0.5)	0.126 N.S.	N.A. N.A.	0.90 (1; N.A.)	N.A. N.A.	N.A. N.A.
Stage cT1c (%)	52.6 (46; 3.5)	57.7 (40; 3.5)	58.5 (18; 4.8)	0.490 N.S.	61.69 (68; 2.7)	58.93 (70; 2.7)	74.64 (122; 1.2)	<0.001 AC/BC^c^	64.09 (16; 3.4)	54.16 (24; 5.0)	68.65 (62; 2.8)	0.027 BC^b^	37.90 (1; N.A.)	48.27 (6; 7.9)	69.54 (8; 4.5)	0.046 BC^b^
Stage cT2 (%)	47.1 (64; 2.5)	39.4 (55; 2.4)	40.6 (19; 4.9)	0.082 N.S.	34.41 (115; 1.6)	40.99 (88; 2.1)	26.67 (166; 1.3)	<0.001 AC/AB/BC^c^	36.95 (29; 2.7)	40.76 (55; 2.7)	37.22 (117; .2.0)	0.536 N.S.	39.30 (4; 7.6)	60.59 (7; 10.1)	33.40 (24; 3.4)	0.008 BC^b^
Stage cT2a (%)	27.6 (33; 1.9)	26.4 (37; 1.7)	25.5 (19; 3.8)	0.836 N.S.	25.48 (41; 1.9)	25.22 (45; 1.5)	19.43 (81; 0.9)	0.001 AC/BC^c^	25.34 (8; 7.6)	25.48 (17; 3.7)	16.23 (38; 1.7)	0.039 N.S. ^b^	19.70 (1; N.A.)	15.50 (3; 2.1)	18.87 (6; 5.5)	0.908 N.S.
Stage cT2b (%)	20.6 (30; 2.8)	11.1 (35; 1.9)	16.3 (17; 3.7)	0.029 AB ^b^	11.75 (32; 1.6)	9.40 (40; 1.5)	5.72 (59; 0.7)	0.002 AC^c^	13.68 (8; 3.8)	12.43 (15; 2.7)	8.05 (35; 1.2)	0.122 N.S.	27.30 (1; N.A.)	18.37 (3; 6.5)	8.60 (6; 2.1)	0.085 N.S.
Stage cT2c (%)	7.7 (17; 1.5)	2.3 (8; 0.9)	1.0 (3; 1.1)	0.028 BC^c^	11.15 (19; 2.5)	8.61 (36; 1.5)	4.98 (24; 0.9)	0.054 N.S.	8.86 (5; 1.9)	14.34 (14; 3.1)	9.38 (28; 1.8)	0.299 N.S.	15.10 (1; N.A.)	39.17 (3; 13.1)	4.07 (7; 1.3)	0.008 BC^b^
Stage cT3 (%)	3.0 (36; 0.5)	11.6 (21; 2.7)	6.5 (10; 2.8)	<0.001 BC^c^	10.65 (61; 2.1)	6.25 (38; 1.1)	2.79 (71; 0.6)	<0.001 AC/BC^c^	9.46 (18; 2.7)	12.14 (36; 2.2)	8.22 (86; 1.2)	0.266 N.S.	7.27 (3; 3.9)	9.75 (4; 2.4)	6.92 (17; 1.8)	0.788 N.S.
Stage cT3a (%)	2.4 (9; 0.7)	14.5 (10; 2.7)	6.6 (7; 3.5)	0.005 AB ^b^	24.87 (6; 12.3)	9.47 (23; 2.1)	4.19 (13; 2.2)	0.018 AC^b^	6.71 (8; 2.7)	12.61 (7; 4.3)	11.64 (18; 1.8)	0.341 N.S.	24.50 (1; N.A.)	18.77 (3; 4.8)	10.35 (4; 6.7)	0.496 N.S.
Stage cT3b (%)	0.0 (5; 0.0)	11.2 (4; 3.5)	0.0 (1; N.A.)	0.020 N.A.	6.27 (3; 3.0)	2.51 (11; 0.9)	2.88 (12; 1.1)	0.311 N.S.	3.38 (4; 2.1)	3.57 (7; 0.9)	3.42 (16; 0.9)	0.994 N.S.	9.80 (1; N.A.)	6.70 (1; N.A.)	8.50 (1; N.A.)	N.A. N.A.
Stage cT4 (%)	0.0 (1; N.A.)	N.A. N.A.	0.0 (1; N.A.)	N.A. N.A.	2.38 (4; 1.2)	N.A. N.A.	2.00 (2; 0.9)	0.854 N.S.	0.93 (3; 0.6)	1.13 (4; 0.7)	0.48 (8; 0.3)	0.610 N.S.	N.A. N.A.	N.A. N.A.	N.A. N.A.	N.A. N.A.
Operative time (min)	186.6 (25; 8.6)	244.0 (81; 8.4)	253.7 (35; 20.6)	0.006 AB ^b^	173.53 (115; 4.3)	216.07 (138; 4.9)	205.46 (248; 3.8)	<0.001 AB/AC^c^	139.76 (33; 10.7)	191.64 (91; 5.9)	179.73 (142; 4.3)	<0.001 AC/AB ^a^	185.72 (6; 31.6)	190.44 (16; 10.0)	190.42 (48; 6.8)	0.975 N.S.
EBL (ml)	1 091.3 (30; 77.2)	488.5 (54; 34.0)	261.0 (34; 43.3)	<0.001 AC/BC^b^	868.88 (130; 34.8)	406.00 (124; 19.0)	242.70 (246; 8.0)	<0.001 AB/BC/AC^c^	534.50 (30; 57.8)	356.90 (69; 27.8)	201.18 (140; 8.9)	<0.001 AB/BC/AC^c^	895.57 (4; 186.7)	364.76 (13; 46.7)	201.50 (34; 14.4)	<0.001 BC^c^
BT rate (%)	21.0 (26; 3.8)	8.2 (66; 1.1)	4.0 (24; 1.7)	<0.001 AB/AC^c^	21.84 (96; 2.1)	6.45 (97; 0.9)	2.92 (129; 0.4)	<0.001 AB/BC/AC^c^	13.34 (30; 1.8)	4.65 (56; 0.6)	2.76 (71; 0.4)	<0.001 AB/AC^c^	12.64 (5; 4.9)	7.47 (9; 2.8)	0.99 (19; 0.3)	0.001 AC/BC^b^
LOS (d)	6.4 (28; 0.8)	5.2 (46; 0.4)	2.7 (27; 0.6)	<0.001 AC/BC^c^	6.27 (88; 0.5)	5.86 (99; 0.4)	2.62 (172; 0.2)	<0.001 AB/BC/AC^c^	5.34 (38; 0.5)	6.23 (69; 0.4)	3.18 (116; 0.2)	<0.001 AC/BC^c^	4.38 (5; 1.1)	4.22 (13; 0.8)	3.83 (23; 0.5)	0.881 N.S.
Catheter time (d)	12.0 (24; 1.0)	7.7 (60; 0.3)	8.1 (27; 0.8)	<0.001 AB/AC^c^	11.12 (46; 0.7)	8.37 (86; 0.3)	7.84 (117; 0.2)	<0.001 AB/AC^c^	9.95 (17; 0.9)	9.16 (63; 0.5)	7.73 (0.5)	0.004 BC^c^	8.00 (3; 0.5)	10.60 (9; 1.1)	7.58 (24; 0.5)	0.026 BC^b^
Complication rate (%)	20.3 (50; 2.3)	16.0 (71; 1.6)	7.6 (24; 1.6)	0.002 AC/BC^c^	17.28 (63; 1.8)	15.80 (101; 1.2)	12.44 (126; 0.8)	0.013 AC^c^	25.42 (30; 3.7)	17.50 (57; 1.3)	14.10 (100; 0.8)	<0.001 AC^c^	23.14 (5; 7.7)	17.39 (14; 2.7)	9.58 (32; 1.5)	0.004 AC/BC^b^

RRP = open radical prostatectomy; LRP = laparoscopic radical prostatectomy; RARP = robot-assisted radical prostatectomy; *N*_r_ = number of reports; SE = standard error of the mean; N.S. = not significant (two-tailed *p* > 0.05); N.A. = not applicable; BMI = body mass index; iPSA = initial prostate-specific antigen; cGS = clinical Gleason score; EBL = estimated blood loss; BT = blood transfusion; LOS = length of stay

† *p* value for analysis of variance with multiple comparison tests among groups A (RRP), B (LRP), and C (RARP) with appropriate correction: a, Tukey; b, Bonferroni; or c, Games-Howell correction.

After simple correlation analysis among the variables studied, only AVSS was significantly correlated with the overall complication rate among the techniques (Table 4).

**Table 4:** Univariate analysis of simple correlation between complication rates and preoperative variables for each surgical technique

Parameter		A: RRP	B: LRP	C: RARP	Significant correlation with complication rate*
AVSS	*r*	−0.274	−0.233	−0.141	
	*p* value	0.023	0.003	0.043	A/B/C
	*N*_r_	69	160	206	
Age (yr)	*r*	0.332*	0.126	0.056	
	*p* value	<0.001	0.056	0.363	A
	*N*_r_	109	229	268	
Body mass index (kg/m^2^)	*r*	−0.025	0.079	0.086	
	*p* value	0.881	0.401	0.217	N.S.
	*r*	38	115	207	
PSA (mg/dl)	*p* value	−0.049	0.001	0.036	
	*N*_r_	0.658	0.997	0.576	N.S.
	N_r_	84	220	247	
PSA <4 mg/dl (%)	*r*	−0.224	−0.006	0.032	
	*p* value	0.461	0.98	0.896	N.S.
	*N*_r_	13	17	19	
PSA 4–10 mg/dl (%)	*r*	−0.534*	0.312	−0.034	
	*p* value	0.049	0.147	0.889	A
	*N*_r_	14	23	19	
PSA 10–20 mg/dl (%)	*r*	0.417	−0.276	−0.264	
	*p* value	0.178	0.181	0.613	N.S.
	*N*_r_	12	25	6	
PSA >20 mg/dl (%)	*r*	−0.639	−0.272	N.A.	
	*p* value	0.246	0.392	N.A.	N.S.
	*N*_r_	5	12	1	
Clinical GS (mean)	*r*	−0.125	−0.001	−0.305*	
	*p* value	0.533	0.993	0.011	C
	*N*_r_	27	105	68	
Clinical GS <7 (%)	*r*	−0.051	−0.194	0.013	
	*p* value	0.745	0.068	0.873	N.S.
	*N*_r_	43	89	150	
Clinical GS 7 (%)	*r*	−0.074	0.234*	0.051	
	*p* value	0.651	0.034	0.543	B
	*N*_r_	40	83	144	
Clinical GS >7 (%)	*r*	0.317	0.063	−0.011	
	*p* value	0.053	0.598	0.892	N.S.
	*N*_r_	38	73	146	
Stage cT1 (%)	*r*	−0.300*	0.129	0.003	
	*p* value	0.018	0.146	0.967	A
	*N*_r_	62	129	145	
Stage cT1a (%)	*r*	−0.217	−0.25	0.276	
	*p* value	0.547	0.317	0.44	N.S.
	*N*_r_	10	18	10	
Stage cT1b (%)	*r*	−0.178	0.129	0.178	
	*p* value	0.581	0.547	0.58	N.S.
	*N*_r_	12	24	12	
Stage cT1c (%)	*r*	−0.384*	0.135	−0.087	
	*p* value	0.009	0.207	0.398	A
	*N*_r_	45	89	97	
Stage cT2 (%)	*r*	0.295*	−0.147	0.019	
	*p* value	0.023	0.103	0.831	A
	*N*_r_	59	125	135	
Stage cT2a (%)	*r*	−0.159	−0.143	0.076	
	*p* value	0.457	0.253	0.537	N.S.
	*N*_r_	24	66	68	
Stage cT2b (%)	*r*	0.007	−0.056	−0.211	
	*p* value	0.975	0.662	0.1	N.S.
	*N*_r_	21	63	62	
Stage cT2c (%)	*r*	0.411	−0.006	0.061	
	*p* value	0.145	0.971	0.719	N.S.
	*N*_r_	14	43	37	
Stage cT3 (%)	*r*	−0.034	−0.114	−0.194	
	*p* value	0.857	0.342	0.097	N.S.
	*N*_r_	30	71	74	
Stage cT3a (%)	*r*	−0.021	−0.169	0.216	
	*p* value	0.953	0.354	0.346	N.S.
	*N*_r_	10	32	21	
Stage cT3b (%)	*r*	0.806	0.149	−0.496	
	*p* value	0.403	0.568	0.085	N.S.
	*N*_r_	3	17	13	
Stage cT4 (%)	*r*	0.25	−1.000*	0.528	
	*p* value	0.685	<0.001	0.224	B
	*N*_r_	5	2	7	

RRP = open radical prostatectomy; LRP = laparoscopic radical prostatectomy; RARP = robot-assisted radical prostatectomy; *r* = correlation coefficient; *N*_r_ = number of reports; N.S. = not significant; N.A. = not applicable; AVSS = annual volume of surgeries per surgeon; PSA = prostate-specific antigen; GS = Gleason score.

* Significant (two-tailed *p* < 0.05).

**Fig. 3:**
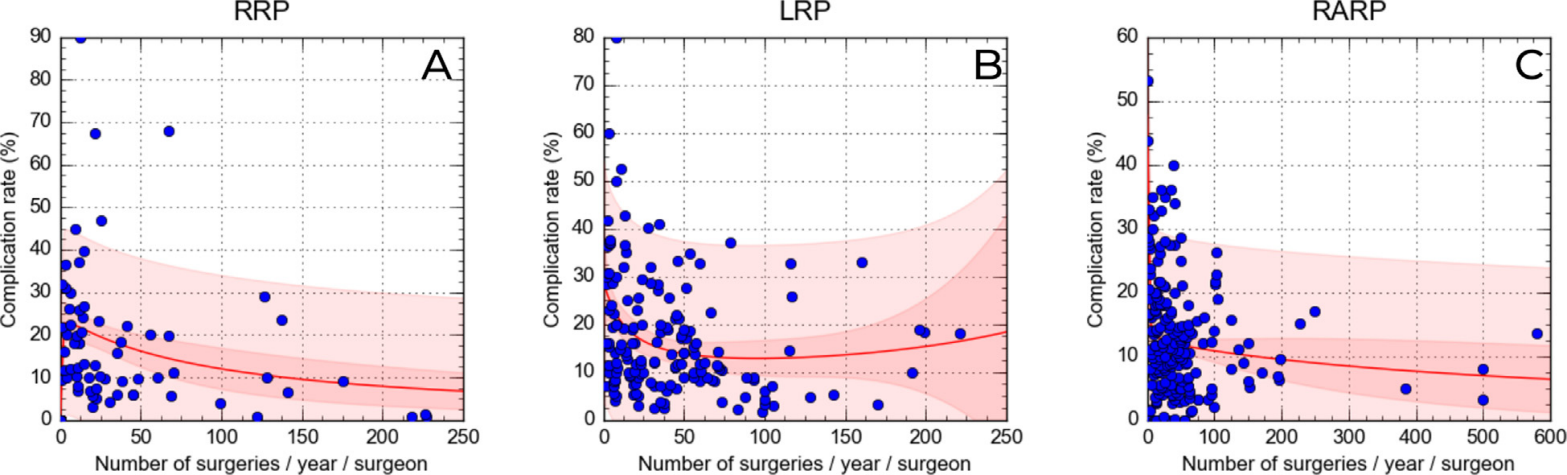
Nonlinear regression models for correlation of complication rates and annual surgery volume per surgeon for each technique. Red lines denote nonlinear regression based on the rational model. RRP = open radical prostatectomy; LRP = laparoscopic radical prostatectomy; RARP = robot-assisted radical prostatectomy.

After nonlinear regression using the rational model, correlations were adjusted to allow prediction of complication rates by AVSS (Table 5). Using this model, AVSS simulation was performed based on the best average AVSS result among the techniques, which was for RARP, with a complication rate of 12.3% for an AVSS of 30.15 surgeries. For RRP, it took 95.33 surgeries/yr per surgeon to achieve a complication rate of 12.3%, and a similar AVSS of 95.41 surgeries/yr for LRP to achieve a complication rate of 12.8% (Fig. 3).

**Table 5:** Univariate analysis of simple correlation between the complication rate and annual surgery volume per surgeon for each operative technique and simulation based on a nonlinear regression model (Fig. 3)

Analysis	Surgical approach		
	RRP	LRP	RARP
Simple correlation			
Pearson correlation coefficient	−0.274	−0.233	−0.141
*p* value	0.023	0.003	0.043
*N*	69	160	206
Curve fit in the rational model			
Correlation coefficient (*r*)	0.67	0.35	0.43
Coefficient of determination (*r*^2^)	0.45	0.12	0.19
Simulation based on the regression model			
Complication rate (%)	12.3	12.8	12.3
Annual surgery volume per surgeon	95.33	95.41	30.15

RRP = open radical prostatectomy; LRP = laparoscopic radical prostatectomy; RARP = robot-assisted radical prostatectomy.

## 4 Discussion

In the current study we applied a new methodology called RSR to capture evidence used in SRs over the 20-yr history of modern RP. Although the current SR methodologies are very effective in filtering the evidence to allow comparison of methodologically similar studies, the need for homogenization often hinders inferences in relation to daily clinical practice, since the scenarios defined in SRs are often not representative of the real world.

In general, our RSR findings corroborate already established evidence that minimally invasive surgery yields better perioperative results in comparison to open surgery. Except for operative time, RARP had superior results to RRP and LRP in terms of the EBL, blood transfusion rate, hospital stay, urinary catheterization time, and complication rate. LRP had a longer operative time, and intermediate results between RRP and RARP for the other variables described above. These advantages explain in part the significantly higher AVSS for RARP.

One of the largest SRs was presented by Novara et al in 2012 [9] and included 110 studies on RARP, for which there was a mean operative time of 152 min, EBL of 166 ml, a transfusion rate of 2%, length of stay of 1.9 d, catheterization time of 6.3 d, and an overall complication rate of 9% (4% grade I, 3% grade II, 2% grade III, 0.4% grade IV, and 0.02% grade V). The meta-analysis comparing the weighted mean difference (WMD) between RRP and RARP showed better results for RARP regarding blood loss (WMD 582.77, 95% confidence interval [CI] 435.25–730.29; *p* < 0.001) and the blood transfusion rate (WMD 7.55, 95% CI 3.65–15.64; *p* < 0.001), but there were no significant differences in operative time (WMD −15.81, 95% CI −68.65 to 37.03; *p* = 0.56) or complication rate (WMD 1.25, 95% CI 0.53–2.93; *p* = 0.61). In comparison, we found worse RARP outcomes according to our analysis of 752 reports, with a mean operative time of 119.8 min, EBL of 228.2 ml, transfusion rate of 2.8%, length of stay of 2.9 d, catheterization time of 7.8 d, and a complication rate of 12.3%.

To date, the largest SR comparing intraoperative and perioperative complications among the three PR techniques was performed by Tewari et al [10] in 2012, which included 39 cohorts for RRP, 57 for LRP, and 42 for RARP. The total intraoperative complication rate was significantly higher for RRP (1.5%) versus RARP (0.4%; *p* < 0.0001) and for LRP (1.6%) versus RARP (0.4%; *p* < 0.0001). There were also significant differences in the total perioperative complication rate for RARP (7.8%) versus RRP (17.9%; *p* < 0.0001) and versus LRP (11.1%; *p* = 0.002). By comparison, we gathered a significantly higher number of reports, with 148 for RRP, 243 for LRP, and 282 for RARP. The increase in the number of reports generated by our approach yielded worse results than those reported by Tewari et al, with overall rates of perioperative complications of 20.2%, 16.3%, and 12.3% for RRP, LRP, and RARP, with significant differences for all three pairwise comparisons (Table 2).

Temporal analysis over the four periods showed that in the first 5 yr after the emergence of minimally invasive surgery, RARP was not used for simpler and low-risk cases, since these patients were more frequent in LRP and RRP study cohorts (Table 3). At this stage, perioperative results with robotic surgery were not very different from those with the other techniques. In the second period, more studies involving patients with low risk undergoing RARP were published, with a consistent improvement in results up to the fourth period. In an analysis of publications carried out by our group [11], we found that the peak for publications on robotic surgery occurred in 2010, between the second and third periods (2005–2015), demonstrating the effort of the scientific community to consolidate RARP as the gold standard [12].

Comparison of the data from our methodology with prior knowledge from the literature reveals that RSR generated worse results for all outcomes, which probably reflects one of the main characteristics of this method. The fact that simple SRs show better results may be because of the need to apply strict criteria for inclusion of studies in the analyses. When several studies are included and a heterogeneous sample of population character is generated, the central limit theorem instantly increases in strength and generates a narrow standard error of the mean, increasing the precision of the population mean, which is then more representative of the real world. Many readers accustomed to the methodological and Cartesian rigor of classical SRs may see this effect as a selection bias; however, other readers who live in a practical real-life world may identify more with RSR results, since these encompass several scenarios that can be extrapolated to daily practice, and the precision and homogeneity of classic SR can be seen as a bias in the same sense.

Our study revealed interesting data regarding the annual volume of surgeries that a surgeon needs to perform to obtain a complication rate similar to the average rate for RARP. To achieve a mean rate of 12.3% for overall complications, a surgeon needs to perform 95 surgeries/yr for RRP and LRP, in contrast to 30 surgeries/yr for RARP. This finding can be interpreted in two ways. A first, more superficial interpretation leads to the conclusion that the RARP learning curve is shorter, as the best complication rate among the three techniques is achieved with a lower frequency of surgeries (average of 2.5 surgeries/mo). However, a second, more in-depth analysis may identify an important selection bias in RARP studies. Considering that the average volume of annual surgeries for RARP is 64, compared to 43 for RRP and 41 for LRP, it is evident that RARP procedures are carried out by surgeons who perform a greater volume of surgeries and are therefore more experienced. In addition, studies on RARP included patients with lower-risk disease, with a higher proportion of patients with PSA <10 mg/dl, Gleason <7, and stage <cT2 (Table 1); in addition, this cohort had a lower rate of lymphadenectomy, which adds complications in the postoperative period. Corroborating this expectation, there was a higher rate of neurovascular bundle preservation for RARP, which is usually performed in patients with lower oncological risk.

In a population study performed before the minimally invasive era, Hu et al [13] analyzed the rates of in-hospital complications for 2292 patients undergoing RRP between 1997 and 1998 in 1210 hospitals using Medicare data. The authors found a complication rate of 21.9% for low-volume (<40 surgeries/yr) and 11.8% for high-volume surgeons (≥40 surgeries/yr), with the latter similar to the rates described for RARP in our study.

The main limitation of our study is inherent to the methodology itself. The fact that the RSR includes all the studies from the SRs, regardless of the inclusion criteria, means that the sample is composed of studies that differ in quality and design. This generates a sample space with as many biases as possible until a population sample that is representative of different clinical scenarios is reached. However, this limitation is purposeful in order to allow readers to understand the power of the population sample and bring the literature data closer to “real-world” findings, since the considerable increase in sample size increases the precision of the population mean according to the central limit theorem. If readers need to compare studies in a homogeneous way, there is already an established methodology that is powerful enough to give such answers—the classic SR with meta-analysis—but with scenarios that are often unrepresentative of the reality in practice for many urologists. The intent of our methodology is not to provide a contrast to data from classic SRs but rather to provide a view of the literature data from a different perspective. If urologists need specific answers, they will certainly find more precise information from SRs with meta-analysis. However, if there is a need for a broader and more representative perspective, the data from this study can be used for comparison of results, including a surgeon’s own results.

Another limitation is related to the presence of weak correlations in the univariate analysis (*r* < 0.39), which indicates that other variables have an influence on the complication rates. This is a consequence of the heterogeneous sample, which is a potential point of criticism. However, because of the high degree of independence and the population nature of the sample, finding a significant correlation, even if weak, made it possible to perform an adjustment in the nonlinear regression with improved correlation and, mainly, with an established clinical logic.

In addition, the narrow standard error of the mean, generated by the population sample over a period of more than 20 yr, makes it statistically practically impossible to change these results, allowing the generation of new reference values to guide patients in the choice between RP techniques.

## 5 Conclusions

Our RSR, which included a wide real-life representative sample and reference values established in the literature, revealed that minimally invasive surgery had the best perioperative and complication results, especially RARP, which was associated with less complex cases, higher annual surgeon volume, and greater performance. To achieve the same levels of complications as with RARP, the annual volume of surgery would need to be three times greater for RRP and LRP, which demonstrates the greater expertise of robotic surgeons compared to surgeons performing the other techniques in the SRs.

Our study can be used as a tool to guide patients and physicians in deciding on the best surgical treatment according to availability. Future studies using the database we constructed for this study could provide information on other oncological and functional outcomes.

  ***Author contributions***: Leonardo Oliveira Reis had full access to all the data in the study and takes responsibility for the integrity of the data and the accuracy of the data analysis.

*Study concept and design*: Reis.

*Acquisition of data*: Moretti.

*Analysis and interpretation of data*: Moretti, Reis, Magna.

*Drafting of the manuscript*: Moretti.

*Critical revision of the manuscript for important intellectual content*: Reis.

*Statistical analysis*: Moretti, Magna.

*Obtaining funding*: Reis.

*Administrative, technical, or material support*: None.

*Supervision*: Reis.

*Other*: None.

  ***Financial disclosures:*** Leonardo Oliveira Reis certifies that all conflicts of interest, including specific financial interests and relationships and affiliations relevant to the subject matter or materials discussed in the manuscript (eg, employment/affiliation, grants or funding, consultancies, honoraria, stock ownership or options, expert testimony, royalties, or patents filed, received, or pending), are the following: None.

  ***Funding/Support and role of the sponsor*:** Leonardo Oliveira Reis is supported by the National Council for Scientific and Technological Development (CNPq Research Productivity 304747/2018-1). The sponsor played no direct role in the study.

  ***Acknowledgments*:** The authors acknowledge the institutions involved, the study patients, and those that provided care for them.

  ***Data sharing statement*:** The data that support the findings of this study are available from the corresponding author on reasonable request.

  ***Ethics considerations*:** The authors certify that the study was performed under the ethics standards laid down in the 1964 Declaration of Helsinki and its later amendments or comparable ethics standards.
