# Supplementary material for: Surgical Results and Complications for Open, Laparoscopic, and Robot-assisted Radical Prostatectomy: A Reverse Systematic Review
Source: Eur Urol Open Sci. 2022 Sep 8;44:150–61. doi: 10.1016/j.euros.2022.08.015 (PMC9468352; doi:10.1016/j.euros.2022.08.015)

**APPENDIX A – METHODOLOGY**

The diagram below represents the path of evidence in the classic Systematic Reviews (SR) and in the reverse systematic review (RSR). The colored circles represent the primary studies available in the literature in different databases (T1, T2..., T8). Blue circles represent SRs available in the literature.

In classic SR, the search starts at the center, through the selection of studies that answer specific questions, generating a corresponding systematic review, with the centrifugal direction, that is, from the center to the periphery.

In the RSR, evidence is captured in a centripetal way. It starts with a search for all systematic reviews on different subjects and we return to the primary works that were chosen by these reviews, composing a heterogeneous and population-based database.


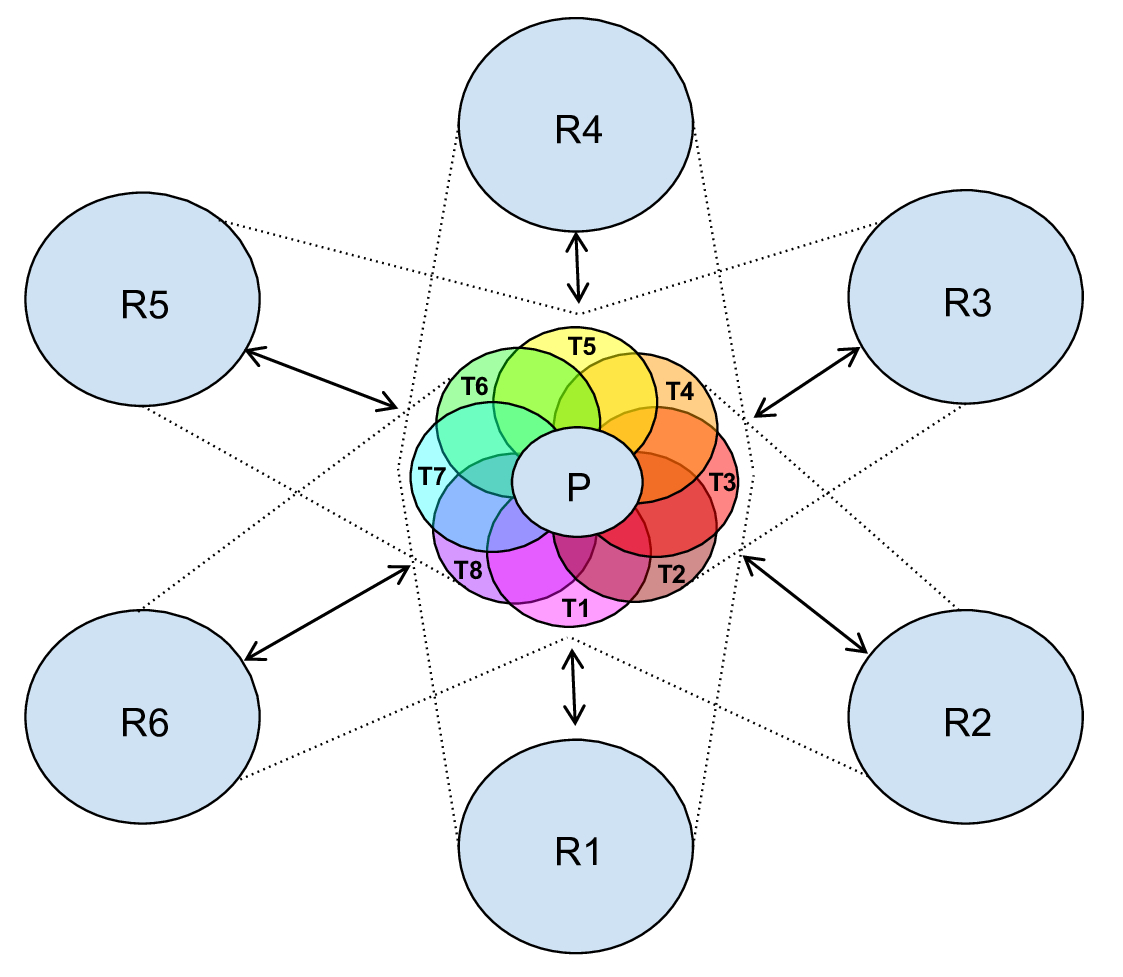

Supplement: Supplementary Appendix A [file mmc1.docx]
