# Supplementary material for: Surgical Results and Complications for Open, Laparoscopic, and Robot-assisted Radical Prostatectomy: A Reverse Systematic Review
Source: Eur Urol Open Sci. 2022 Sep 8;44:150–61. doi: 10.1016/j.euros.2022.08.015 (PMC9468352; doi:10.1016/j.euros.2022.08.015)
Supplement: Supplementary Appendix B [file mmc2.docx]

**APPENDIX B – SEARCH STRATEGY**

Search strategy was systematized in 8 databases described below and were done using health descriptors specific to each base. It was complemented with use of synonyms in open search in title, abstract and subject through Boolean indicators.

**1) Health Science Descriptors**

| **Search Base** | **Vocabulary of subjects** | **Health Science Descriptors** | | |
| --- | --- | --- | --- | --- |
|  |  | **1** | **2** | **3** |
| **PUBMED** | MeSh | *Prostatectomy* | *Laparoscopy* | *Robotic*  *Surgical*  *Procedures* |
| **BVS/BIREME** | DeCS | *Prostatectomy* | *Laparoscopy* | *Robotic*  *Surgical*  *Procedures* |
| **SCOPUS** | -- | *Prostatectomy* | *Laparoscopy* | *Robotic*  *Surgical*  *Procedures* |
| **WEB OF SCIENCE** | -- | *Prostatectomy* | *Laparoscopic* | *Robotic*  *Surgical*  *Procedures* |
| **EMBASE** | Emtree | *Prostatectomy* | *Laparoscopy* | *Robotic*  *Surgical*  *Procedures*  *Use preferred term: Robotic Surgical Procedure* |
| **COCHRANE LIBRARY** | MeSh | *Prostatectomy* | *Laparoscopy* | *Robotic*  *Surgical*  *Procedures* |
| **PROQUEST** | MeSh | *Prostatectomy* | *Laparoscopy* | *Robotic*  *Surgical*  *Procedures* |
| **CINAHL** | MH | *Prostatectomy* | *Laparoscopy* | *Robotic*  *Surgical*  *Procedures* |

**2) Key-words (synonymous):**

**- *Prostatectomy*:** Prostatectomy OR Prostatectomies OR "Prostatectomy, Suprapubic" OR "Prostatectomies, Suprapubic" OR "Suprapubic Prostatectomies" OR "Suprapubic Prostatectomy" OR "Prostatectomy, Retropubic" OR "Prostatectomies, Retropubic" OR "Retropubic Prostatectomies" OR "Retropubic Prostatectomy".

**- *Laparoscopy:*** Laparoscopy OR Laparoscopies OR Celioscopy OR Celioscopies OR Peritoneoscopy OR Peritoneoscopies OR "Surgical Procedures, Laparoscopic" OR "Laparoscopic Surgical Procedure" OR "Procedure, Laparoscopic Surgical" OR "Procedures, Laparoscopic Surgical" OR "Surgery, Laparoscopic" OR "Laparoscopic Surgical Procedures" OR "Laparoscopic Surgery" OR "Laparoscopic Surgeries" OR "Surgeries, Laparoscopic" OR "Laparoscopic Assisted Surgery" OR "Laparoscopic Assisted Surgeries" OR "Surgeries, Laparoscopic Assisted" OR "Surgery, Laparoscopic Assisted" OR "Surgical Procedure, Laparoscopic".

**- *Robotic Surgical Procedures*:** "Robotic Surgical Procedures" OR "Procedure, Robotic Surgical" OR "Procedures, Robotic Surgical" OR "Robotic Surgical Procedure" OR "Surgical Procedure, Robotic" OR "Surgical Procedures, Robotic".

**- *Cystectomy*:** Cystectomy OR Cystectomies.

**3) Free Terms (not descriptors)**

**- All the search bases:** "laparoscopic radical prostatectomy (LRP)" OR LRP OR "laparoscopic assisted radical prostatectomy" OR "Robot-assisted laparoscopic radical prostatectomy (RALRP)" OR "Robot assisted laparoscopic radical prostatectomy (RALRP)" OR RALRP OR "Robotic assisted laparoscopic prostatectomy (RALP)" OR "robot-assisted radical prostatectomy (RARP)" OR "robot assisted radical prostatectomy (RARP)" OR RARP OR "Endoscopic extraperitoneal radical prostatectomy (EERP)" OR EERP OR "Endoscopic extraperitoneal radical prostatectomy (EERPE)" OR EERPE OR "Robot-assisted" OR "Robot assisted" OR "robotic prostatectomy" OR "radical prostatectomy (RP)" OR "radical prostatectomy" OR RP OR RRP OR "retropubic radical prostatectomy" OR "open prostatectomy" OR "laparoscopic radical prostatectomy" OR "robot-assisted prostatectomy" OR "Robot-assisted laparoscopic radical prostatectomy (RALRP)" OR "Robot assisted laparoscopic radical prostatectomy (RALRP)" OR "Robot assisted laparoscopic radical prostatectomy" OR "Robot-assisted laparoscopic radical prostatectomy" OR RALRP OR "Robotic assisted laparoscopic prostatectomy (RALP)" OR "Robot-assisted laparoscopic prostatectomy (RALP)" OR "Robot assisted laparoscopic prostatectomy" OR "Robot-assisted laparoscopic prostatectomy" OR RALP OR "Robot-assisted radical prostatectomy (RARP)" OR "Robot assisted radical prostatectomy (RARP)" OR "Robot-assisted radical prostatectomy" OR "Robot assisted radical prostatectomy" OR RARP OR **"**Robotic prostatectomy".

**- Embase: “**robot-assisted prostatectomy” OR “robot-assisted prostatectomy” OR “laparoscopic radical prostatectomy” OR **"**Robotic radical prostatectomy" OR "Robotic-assisted radical prostatectomy" OR "Robotic assisted radical prostatectomy".

**4) *Systematic Review* Filter**

**- PubMed:** (systematic review [ti] OR meta-analysis [pt] OR meta-analysis [ti] OR systematic literature review [ti] OR this systematic review [tw] OR pooling project [tw] OR (systematic review [tiab] AND review [pt]) OR meta synthesis [ti] OR meta-analy*[ti] OR integrative review [tw] OR integrative research review [tw] OR rapid review [tw] OR umbrella review [tw] OR consensus development conference [pt] OR practice guideline [pt] OR drug class reviews [ti] OR cochrane database syst rev [ta] OR acp journal club [ta] OR health technol assess [ta] OR evid rep technol assess summ [ta] OR jbi database system rev implement rep [ta]) OR (clinical guideline [tw] AND management [tw]) OR ((evidence based[ti] OR evidence-based medicine [mh] OR best practice* [ti] OR evidence synthesis [tiab]) AND (review [pt] OR diseases category[mh] OR behavior and behavior mechanisms [mh] OR therapeutics [mh] OR evaluation studies[pt] OR validation studies[pt] OR guideline [pt] OR pmcbook)) OR ((systematic [tw] OR systematically [tw] OR critical [tiab] OR (study selection [tw]) OR (predetermined [tw] OR inclusion [tw] AND criteri* [tw]) OR exclusion criteri* [tw] OR main outcome measures [tw] OR standard of care [tw] OR standards of care [tw]) AND (survey [tiab] OR surveys [tiab] OR overview* [tw] OR review [tiab] OR reviews [tiab] OR search* [tw] OR handsearch [tw] OR analysis [ti] OR critique [tiab] OR appraisal [tw] OR (reduction [tw]AND (risk [mh] OR risk [tw]) AND (death OR recurrence))) AND (literature [tiab] OR articles [tiab] OR publications [tiab] OR publication [tiab] OR bibliography [tiab] OR bibliographies [tiab] OR published [tiab] OR pooled data [tw] OR unpublished [tw] OR citation [tw] OR citations [tw] OR database [tiab] OR internet [tiab] OR textbooks [tiab] OR references [tw] OR scales [tw] OR papers [tw] OR datasets [tw] OR trials [tiab] OR meta-analy* [tw] OR (clinical [tiab] AND studies [tiab]) OR treatment outcome [mh] OR treatment outcome [tw] OR pmcbook)) NOT (letter [pt] OR newspaper article [pt]).

Available in: (https://www.nlm.nih.gov/bsd/pubmed_subsets/sysreviews_strategy.html)

**5) Filters**

**- Search period:** from January 1, 2000 to December 5, 2020.

**- Language:** English

**- Type of study:** Systematic Review and Meta-analysis

**- Species:** Human

**6) Search Strategy**

Each specific search strategy for each database is described below, with the date of the search and the number of articles found:

**1 – PUBMED/PMC (MEDLINE)**

- **Search Date:** December 5, 2020.
- **Number of articles found: 128**
- **Search Strategy:** ((((((((Robotics[MeSH Terms]) OR Robotics[Title/Abstract])) OR (((((((Robotic Surgical Procedures[MeSH Terms]) OR "Robotic Surgical Procedures "[Title/Abstract]) OR "Procedure, Robotic Surgical"[Title/Abstract]) OR "Procedures, Robotic Surgical"[Title/Abstract]) OR "Robotic Surgical Procedure"[Title/Abstract]) OR "Surgical Procedure, Robotic"[Title/Abstract]) OR "Surgical Procedures, Robotic"[Title/Abstract])) OR (((((((((((("Robot-assisted laparoscopic radical prostatectomy (RALRP)") OR "Robot assisted laparoscopic radical prostatectomy (RALRP)") OR "Robot assisted laparoscopic radical prostatectomy") OR "Robot-assisted laparoscopic radical prostatectomy") OR RALRP)) OR ((((("Robot-assisted radical prostatectomy (RARP)") OR "Robot assisted radical prostatectomy (RARP)") OR "Robot-assisted radical prostatectomy") OR "Robot assisted radical prostatectomy") OR RARP)) OR "Robotic prostatectomy") OR (("Robot-assisted prostatectomy") OR "Robot assisted prostatectomy")) OR "Robotic radical prostatectomy") OR (("Robotic-assisted radical prostatectomy") OR "Robotic assisted radical prostatectomy")) OR (((((((("Robotic assisted laparoscopic prostatectomy (RALP)") OR "Robot-assisted laparoscopic prostatectomy (RALP)") OR "Robot assisted laparoscopic prostatectomy") OR "Robot-assisted laparoscopic prostatectomy") OR RALP)))))) AND (((((((((((Prostatectomy[MeSH Terms]) OR Prostatectomy[Title/Abstract]) OR Prostatectomies[Title/Abstract]) OR "Prostatectomy, Suprapubic"[Title/Abstract]) OR "Prostatectomies, Suprapubic"[Title/Abstract]) OR "Suprapubic Prostatectomies"[Title/Abstract]) OR "Suprapubic Prostatectomy"[Title/Abstract]) OR "Prostatectomy, Retropubic"[Title/Abstract]) OR "Prostatectomies, Retropubic"[Title/Abstract]) OR "Retropubic Prostatectomies"[Title/Abstract]) OR "Retropubic Prostatectomy"[Title/Abstract]))) AND systematic [sb] Filters: Publication date from 2000/01/01 to 2020/12/05; Humans; English.

**2 - BVS / BIREME**

- **Search Date:** December, 2020
- **Number of articles found:** 87 (MEDLINE, LILACS, IBECS)
- **Search Strategy:** (tw:(prostatectomy OR prostatectomies OR "Prostatectomy, Suprapubic" OR "Prostatectomies, Suprapubic" OR "Suprapubic Prostatectomies" OR "Suprapubic Prostatectomy" OR "Prostatectomy, Retropubic" OR "Prostatectomies, Retropubic" OR "Retropubic Prostatectomies" OR "Retropubic Prostatectomy" )) AND (tw:(robotics)) OR (tw:("Robot-assisted laparoscopic radical prostatectomy (RALRP)" OR "Robot assisted laparoscopic radical prostatectomy (RALRP)" OR "Robot assisted laparoscopic radical prostatectomy" OR "Robot-assisted laparoscopic radical prostatectomy" OR ralrp )) OR (tw:("Robotic assisted laparoscopic prostatectomy (RALP)" OR "Robot-assisted laparoscopic prostatectomy (RALP)" OR "Robot assisted laparoscopic prostatectomy" OR "Robot-assisted laparoscopic prostatectomy" OR ralp)) OR (tw:("Robot-assisted radical prostatectomy (RARP)" OR "Robot assisted radical prostatectomy (RARP)" OR "Robot-assisted radical prostatectomy" OR "Robot assisted radical prostatectomy" OR rarp)) OR (tw:("Robotic prostatectomy" )) OR (tw:("Robot-assisted prostatectomy"or "Robot assisted prostatectomy")) OR (tw:("Robotic radical prostatectomy")) OR (tw:("Robotic-assisted radical prostatectomy" OR "Robotic assisted radical prostatectomy")) OR (tw:("Robotic Surgical Procedures" OR "Procedure, Robotic Surgical" OR "Procedures, Robotic Surgical" OR "Robotic Surgical Procedure" OR "Surgical Procedure, Robotic" OR "Surgical Procedures, Robotic")) AND (tw:("SYSTEMATIC REVIEWS" OR "SYSTEMATIC REVIEW" )) AND (instance:"regional") AND ( la:("en"))

**3 - CINAHL** (The Cumulative Index to Nursing and Allied Health Literature)

- **Search Date:** December 5, 2020
- **Number of articles found:** 05
- **Search Strategy:** (MH "Prostatectomy") OR "Prostatectomy" OR Prostatectomy OR Prostatectomies OR "Prostatectomy, Suprapubic" OR "Prostatectomies, Suprapubic" OR "Suprapubic Prostatectomies" OR "Suprapubic Prostatectomy" OR "Prostatectomy, Retropubic" OR "Prostatectomies, Retropubic" OR "Retropubic Prostatectomies" OR "Retropubic Prostatectomy" AND (MH "Robotics") OR "Robotics" OR ( "Robot-assisted laparoscopic radical prostatectomy (RALRP)" OR "Robot assisted laparoscopic radical prostatectomy (RALRP)" OR "Robot assisted laparoscopic radical prostatectomy" OR "Robot-assisted laparoscopic radical prostatectomy" OR RALRP ) OR ( "Robotic assisted laparoscopic prostatectomy (RALP)" OR "Robot-assisted laparoscopic prostatectomy (RALP)" OR "Robot assisted laparoscopic prostatectomy" OR "Robot-assisted laparoscopic prostatectomy" OR RALP ) OR ( "Robot-assisted radical prostatectomy (RARP)" OR "Robot assisted radical prostatectomy (RARP)" OR "Robot-assisted radical prostatectomy" OR "Robot assisted radical prostatectomy" OR RARP ) OR "Robotic prostatectomy" OR "Robot-assisted prostatectomy" OR "Robot assisted prostatectomy" OR "Robotic radical prostatectomy" OR ( "Robotic-assisted radical prostatectomy" OR "Robotic assisted radical prostatectomy" ) OR (MH "Robotic Surgical Procedures") OR "Robotic Surgical Procedures" OR "Robotic Surgical Procedures" OR "Procedure, Robotic Surgical" OR "Procedures, Robotic Surgical" OR "Robotic Surgical Procedure" OR "Surgical Procedure, Robotic" OR "Surgical Procedures, Robotic" AND TI "SYSTEMATIC REVIEWS" OR TI "SYSTEMATIC REVIEW"

**4 - WEB OF SCIENCE**

- **Search Date:** December 5, 2020
- **Number of articles found:** 135
- **Search Strategy:** (("Robot-assisted laparoscopic radical prostatectomy (RALRP)" OR "Robot assisted laparoscopic radical prostatectomy (RALRP)" OR "Robot assisted laparoscopic radical prostatectomy" OR "Robot-assisted laparoscopic radical prostatectomy" OR RALRP) OR ("Robotic assisted laparoscopic prostatectomy (RALP)" OR "Robot-assisted laparoscopic prostatectomy (RALP)" OR "Robot assisted laparoscopic prostatectomy" OR "Robot-assisted laparoscopic prostatectomy" OR RALP) OR ("Robot-assisted radical prostatectomy (RARP)" OR "Robot assisted radical prostatectomy (RARP)" OR "Robot-assisted radical prostatectomy" OR "Robot assisted radical prostatectomy" OR RARP) OR ("Robotic prostatectomy") OR ("Robot-assisted prostatectomy" OR "Robot assisted prostatectomy") OR ("Robotic radical prostatectomy") OR ("Robotic-assisted radical prostatectomy" OR "Robotic assisted radical prostatectomy")) OR (("Robotic Surgical Procedures" OR "Procedure, Robotic Surgical" OR "Procedures, Robotic Surgical" OR "Robotic Surgical Procedure" OR "Surgical Procedure, Robotic" OR "Surgical Procedures, Robotic")) OR (Robotics)) AND (Prostatectomy OR Prostatectomies OR "Prostatectomy, Suprapubic" OR "Prostatectomies, Suprapubic" OR "Suprapubic Prostatectomies" OR "Suprapubic Prostatectomy" OR "Prostatectomy, Retropubic" OR "Prostatectomies, Retropubic" OR "Retropubic Prostatectomies" OR "Retropubic Prostatectomy") AND ( 2020 OR 2019 OR 2012 OR 2005 OR 2018 OR 2011 OR 2004 OR 2017 OR 2010 OR 2003 OR 2016 OR 2009 OR 2002 OR 2015 OR 2008 OR 2001 OR 2014 OR 2007 OR 2000 OR 2013 OR 2006 ) AND ("SYSTEMATIC REVIEWS" OR "SYSTEMATIC REVIEW")

**5 – EMBASE**

- **Search Date:** December 5, 2020
- **Number of articles found:** 137
- **Search Strategy:** ('prostatectomy'/exp OR 'prostatectomy'/syn) AND ('systematic reviews':ab,ti OR 'systematic review':ab,ti) AND ('robotics'/exp OR 'robotic assisted laparoscopic prostatectomy'/exp OR 'robotic assisted laparoscopic prostatectomy (ralp)' OR 'robot-assisted laparoscopic prostatectomy (ralp)' OR 'robot assisted laparoscopic prostatectomy'/exp OR 'robot assisted laparoscopic prostatectomy' OR 'robot-assisted laparoscopic prostatectomy'/exp OR 'robot-assisted laparoscopic prostatectomy' OR ralp OR 'robot-assisted prostatectomy'/exp OR 'robot-assisted prostatectomy'/syn OR 'robot-assisted laparoscopic radical prostatectomy (ralrp)' OR 'robot assisted laparoscopic radical prostatectomy (ralrp)' OR 'robot assisted laparoscopic radical prostatectomy'/exp OR 'robot assisted laparoscopic radical prostatectomy' OR 'robot-assisted laparoscopic radical prostatectomy'/exp OR 'robot-assisted laparoscopic radical prostatectomy' OR ralrp OR 'robot-assisted radical prostatectomy (rarp)' OR 'robot assisted radical prostatectomy (rarp)' OR 'robot-assisted radical prostatectomy'/exp OR 'robot-assisted radical prostatectomy' OR 'robot assisted radical prostatectomy'/exp OR 'robot assisted radical prostatectomy' OR rarp OR 'robotic prostatectomy'/exp OR 'robotic prostatectomy' OR 'robotic radical prostatectomy'/exp OR 'robotic radical prostatectomy' OR 'robotic-assisted radical prostatectomy'/exp OR 'robotic-assisted radical prostatectomy' OR 'robotic assisted radical prostatectomy'/exp OR 'robotic assisted radical prostatectomy' OR 'robotic surgical procedure'/exp OR 'robotic surgical procedure'/syn) AND [english]/lim AND (2000:py OR 2001:py OR 2002:py OR 2003:py OR 2006:py OR 2007:py OR 2008:py OR 2009:py OR 2010:py OR 2011:py OR 2012:py OR 2013:py OR 2014:py OR 2015:py OR 2016:py OR 2017:py OR 2018:py OR 2019:py OR 2020:py)

**6 - COCHRANE LIBRARY**

- **Search date:** December 5, 2020
- **Number of articles found:**19
- **Search Strategy:**

MeSH descriptor: [Prostatectomy] explode all trees OR (Prostatectomy OR Prostatectomies OR "Prostatectomy, Suprapubic" OR "Prostatectomies, Suprapubic" OR "Suprapubic Prostatectomies" OR "Suprapubic Prostatectomy" OR "Prostatectomy, Retropubic" OR "Prostatectomies, Retropubic" OR "Retropubic Prostatectomies" OR "Retropubic Prostatectomy"):ti,ab,kw AND MeSH descriptor: [Robotics] explode all trees OR (Robotics):ti,ab,kw OR ("Robot-assisted laparoscopic radical prostatectomy (RALRP)" OR "Robot assisted laparoscopic radical prostatectomy (RALRP)" OR "Robot assisted laparoscopic radical prostatectomy" OR "Robot-assisted laparoscopic radical prostatectomy" OR RALRP) OR ("Robotic assisted laparoscopic prostatectomy (RALP)" OR "Robot-assisted laparoscopic prostatectomy (RALP)" OR "Robot assisted laparoscopic prostatectomy" OR "Robot-assisted laparoscopic prostatectomy" OR RALP) OR ("Robot-assisted radical prostatectomy (RARP)" OR "Robot assisted radical prostatectomy (RARP)" OR "Robot-assisted radical prostatectomy" OR "Robot assisted radical prostatectomy" OR RARP) OR ("Robotic prostatectomy") OR ("Robot-assisted prostatectomy"OR "Robot assisted prostatectomy") OR ("Robotic radical prostatectomy") OR ("Robotic-assisted radical prostatectomy" OR "Robotic assisted radical prostatectomy") OR MeSH descriptor: [Robotic Surgical Procedures] explode all trees OR ("Robotic Surgical Procedures" OR "Procedure, Robotic Surgical" OR "Procedures, Robotic Surgical" OR "Robotic Surgical Procedure" OR "Surgical Procedure, Robotic" OR "Surgical Procedures, Robotic"):ti,ab,kw AND ("SYSTEMATIC REVIEWS"):ti,ab,kw OR ("SYSTEMATIC REVIEW"):ti,ab,kw

**7 – PROQUEST CENTRAL**

- **Search Date:** December 5, 2020
- **Number of articles found:** 116
- **Search Strategy:** ((MJMESH.EXACT.EXPLODE("Prostatectomy") OR (Prostatectomy OR Prostatectomies OR "Prostatectomy, Suprapubic" OR "Prostatectomies, Suprapubic" OR "Suprapubic Prostatectomies" OR "Suprapubic Prostatectomy" OR "Prostatectomy, Retropubic" OR "Prostatectomies, Retropubic" OR "Retropubic Prostatectomies" OR "Retropubic Prostatectomy")) AND ((MESH.EXACT("Robotics") OR Robotics) OR (("Robot-assisted laparoscopic radical prostatectomy (RALRP)" OR "Robot assisted laparoscopic radical prostatectomy (RALRP)" OR "Robot assisted laparoscopic radical prostatectomy" OR "Robot-assisted laparoscopic radical prostatectomy" OR RALRP) OR ("Robotic assisted laparoscopic prostatectomy (RALP)" OR "Robot-assisted laparoscopic prostatectomy (RALP)" OR "Robot assisted laparoscopic prostatectomy" OR "Robot-assisted laparoscopic prostatectomy" OR RALP) OR ("Robot-assisted radical prostatectomy (RARP)" OR "Robot assisted radical prostatectomy (RARP)" OR "Robot-assisted radical prostatectomy" OR "Robot assisted radical prostatectomy" OR RARP) OR "Robotic prostatectomy" OR ("Robot-assisted prostatectomy" OR "Robot assisted prostatectomy") OR "Robotic radical prostatectomy" OR ("Robotic-assisted radical prostatectomy" OR "Robotic assisted radical prostatectomy")) OR ((MJMESH.EXACT.EXPLODE("Robotic Surgical Procedures:E.04.749.500") OR MJMESH.EXACT.EXPLODE("Robotic Surgical Procedures:L.01.313.500.750.100.710.800.500") OR MJMESH.EXACT.EXPLODE("Robotic Surgical Procedures:E.02.950.875.500")) OR ("Robotic Surgical Procedures" OR "Procedure, Robotic Surgical" OR "Procedures, Robotic Surgical" OR "Robotic Surgical Procedure" OR "Surgical Procedure, Robotic" OR "Surgical Procedures, Robotic")))) AND (ti("SYSTEMATIC REVIEWS") OR ti("SYSTEMATIC REVIEW")) AND (la.exact("ENG") AND pd(20000101-20201205))

**8 – SCOPUS**

- **Search date:** December 5, 2020
- **Number of articles found:** 7
- **Search Strategy:** (TITLE-ABS-KEY(Prostatectomy OR Prostatectomies OR "Prostatectomy, Suprapubic" OR "Prostatectomies, Suprapubic" OR "Suprapubic Prostatectomies" OR "Suprapubic Prostatectomy" OR "Prostatectomy, Retropubic" OR "Prostatectomies, Retropubic" OR "Retropubic Prostatectomies" O)) and ((TITLE-ABS-KEY(Robotics)) or ((ALL("Robot-assisted laparoscopic radical prostatectomy (RALRP)" OR "Robot assisted laparoscopic radical prostatectomy (RALRP)" OR "Robot assisted laparoscopic radical prostatectomy" OR "Robot-assisted laparoscopic radical prostatectomy" OR RALRP ) OR ALL("Robotic assisted laparoscopic prostatectomy (RALP)" OR "Robot-assisted laparoscopic prostatectomy (RALP)" OR "Robot assisted laparoscopic prostatectomy" OR "Robot-assisted laparoscopic prostatectomy" OR RALP) OR ALL("Robot-assisted radical prostatectomy (RARP)" OR "Robot assisted radical prostatectomy (RARP)" OR "Robot-assisted radical prostatectomy" OR "Robot assisted radical prostatectomy" OR RARP) OR ALL("Robotic prostatectomy" ) OR ALL("Robot-assisted prostatectomy" OR "Robot assisted prostatectomy") OR ALL("Robotic radical prostatectomy") OR ALL("Robotic-assisted radical prostatectomy" OR "Robotic assisted radical prostatectomy"))) or (TITLE-ABS-KEY("Robotic Surgical Procedures" OR "Procedure, Robotic Surgical" OR "Procedures, Robotic Surgical" OR "Robotic Surgical Procedure" OR "Surgical Procedure, Robotic" OR "Surgical Procedures, Robotic"))) and ((TITLE-ABS-KEY("SYSTEMATIC REVIEWS") OR TITLE-ABS-KEY("SYSTEMATIC REVIEW"))
