# Supplementary material for: Surgical Results and Complications for Open, Laparoscopic, and Robot-assisted Radical Prostatectomy: A Reverse Systematic Review
Source: Eur Urol Open Sci. 2022 Sep 8;44:150–61. doi: 10.1016/j.euros.2022.08.015 (PMC9468352; doi:10.1016/j.euros.2022.08.015)
Supplement: Supplementary Appendix C [file mmc3.docx]

**APPENDIX C - INCLUDED SYSTEMATIC REVIEWS**

Selected systematic review studies for analysis are listed below (A^1-80^):

1. Abboudi H, Khan MS, Guru KA, et al. Learning curves for urological procedures: A systematic review. Review. BJU International. 2014;114(4):617-629. doi:10.1111/bju.12315

2. Allan C, Ilic D. Laparoscopic versus Robotic-Assisted Radical Prostatectomy for the Treatment of Localised Prostate Cancer: A Systematic Review. Review. Urologia Internationalis. 2016;96(4):373-378. doi:10.1159/000435861

3. Autorino R, Zargar H, White WM, et al. Current applications of near-infrared fluorescence imaging in robotic urologic surgery: A systematic review and critical analysis of the literature. Review. Urology. 2014;84(4):751-759. doi:10.1016/j.urology.2014.05.059

4. Bai Y, Pu C, Yuan H, et al. Assessing the Impact of Barbed Suture on Vesicourethral Anastomosis During Minimally Invasive Radical Prostatectomy: A Systematic Review and Meta-analysis. Urology. Jun 2015;85(6):1368-75. doi:10.1016/j.urology.2015.02.033

5. Baladakis J, Perera M, Bolton D, Lawrentschuk N, Adam A. Is There an Optimal Curative Option in HIV-Positive Men with Localized Prostate Cancer? A Systematic Review. Curr Urol. Jul 2019;12(4):169-176. doi:10.1159/000499309

6. Bellangino M, Verrill C, Leslie T, Bell RW, Hamdy FC, Lamb AD. Systematic Review of Studies Reporting Positive Surgical Margins After Bladder Neck Sparing Radical Prostatectomy. Review. Current Urology Reports. 2017;18(12)doi:10.1007/s11934-017-0745-0

7. Berryhill R, Jhaveri J, Yadav R, et al. Robotic prostatectomy: a review of outcomes compared with laparoscopic and open approaches. Urology. Jul 2008;72(1):15-23. doi:10.1016/j.urology.2007.12.038

8. Bertolo R, Tracey A, Dasgupta P, et al. Supra-pubic versus urethral catheter after robot-assisted radical prostatectomy: systematic review of current evidence. Review. World Journal of Urology. Sep 2018;36(9):1365-1372. doi:10.1007/s00345-018-2275-x

9. Bertolo R, Hung A, Porpiglia F, Bove P, Schleicher M, Dasgupta P. Systematic review of augmented reality in urological interventions: the evidences of an impact on surgical outcomes are yet to come. World Journal of Urology. Mar 2019 2019:1-10. doi:http://dx.doi.org/10.1007/s00345-019-02711-z

10. Cao L, Yang Z, Qi L, Chen M. Robot-assisted and laparoscopic vs open radical prostatectomy in clinically localized prostate cancer: perioperative, functional, and oncological outcomes: A Systematic review and meta-analysis. Medicine (Baltimore). May 2019;98(22):e15770. doi:10.1097/MD.0000000000015770

11. Carneiro A, Cha JD, Baccaglini W, et al. Should aspirin be suspended prior to robot-assisted radical prostatectomy? A systematic review and meta-analysis. Review. Therapeutic Advances in Urology. 2019;11doi:10.1177/1756287218816595

12. Cathcart P, Murphy DG, Moon D, Costello AJ, Frydenberg M. Perioperative, functional and oncological outcomes after open and minimally invasive prostate cancer surgery: experience from Australasia. BJU Int. Apr 2011;107 Suppl 3:11-9. doi:10.1111/j.1464-410X.2011.10053.x

13. Checcucci E, Amparore D, De Luca S, Autorino R, Fiori C, Porpiglia F. Precision prostate cancer surgery: an overview of new technologies and techniques. Minerva Urol Nefrol. Oct 2019;71(5):487-501. doi:10.23736/S0393-2249.19.03365-4

14. Checcucci E, Veccia A, Fiori C, et al. Retzius-sparing robot-assisted radical prostatectomy vs the standard approach: a systematic review and analysis of comparative outcomes. BJU Int. Jan 2020;125(1):8-16. doi:10.1111/bju.14887

15. Choo MSMD, Kim MMD, Ku JHMDP, Kwak CMDP, Kim HHMDP, Jeong CWMDP. Extended versus Standard Pelvic Lymph Node Dissection in Radical Prostatectomy on Oncological and Functional Outcomes: A Systematic Review and Meta-Analysis. Annals of Surgical Oncology. 2017;24(7):2047-2054. doi:http://dx.doi.org/10.1245/s10434-017-5822-6

16. Coelho RF, Rocco B, Patel MB, et al. Retropubic, laparoscopic, and robot-assisted radical prostatectomy: a critical review of outcomes reported by high-volume centers. J Endourol. Dec 2010;24(12):2003-15. doi:10.1089/end.2010.0295

17. De Carlo F, Celestino F, Verri C, Masedu F, Liberati E, Di Stasi SM. Retropubic, laparoscopic, and robot-assisted radical prostatectomy: Surgical, oncological, and functional outcomes: A systematic review. Review. Urologia Internationalis. 2014;93(4):373-383. doi:10.1159/000366008

18. De Hong C, Liang Ren L, Qiang W, et al. Comparison of efficacy and safety of conventional laparoscopic radical prostatectomy by the transperitoneal versus extraperitoneal procedure. Sci Rep. Oct 2015;5:14442. doi:10.1038/srep14442

19. Du Y, Long Q, Guan B, et al. Robot-Assisted Radical Prostatectomy Is More Beneficial for Prostate Cancer Patients: A System Review and Meta-Analysis. Med Sci Monit. 2018/01 2018;24:272-287.

20. Fernando H, Garcia C, Hossack T, et al. Incidence, Predictive Factors and Preventive Measures for Inguinal Hernia following Robotic and Laparoscopic Radical Prostatectomy: A Systematic Review. J Urol. 06 2019;201(6):1072-1079. doi:10.1097/JU.0000000000000133

21. Ferronha F, Barros F, Santos VV, Ravery V, Delmas V. Is there any evidence of superiority between retropubic, laparoscopic or robot-assisted radical prostatectomy? Int Braz J Urol. 2011 Mar-Apr 2011;37(2):146-58; discussion 159-60.

22. Ficarra V, Cavalleri S, Novara G, Aragona M, Artibani W. Evidence from Robot-Assisted Laparoscopic Radical Prostatectomy: A Systematic Review. Review. European Urology. 2007;51(1):45-56. doi:10.1016/j.eururo.2006.06.017

23. Ficarra V, Novara G, Ahlering TE, et al. Systematic review and meta-analysis of studies reporting potency rates after robot-assisted radical prostatectomy. Review. European Urology. 2012;62(3):418-430. doi:10.1016/j.eururo.2012.05.046

24. Ficarra V, Novara G, Artibani W, et al. Retropubic, Laparoscopic, and Robot-Assisted Radical Prostatectomy: A Systematic Review and Cumulative Analysis of Comparative Studies. Review. European Urology. 2009;55(5):1037-1063. doi:10.1016/j.eururo.2009.01.036

25. Ficarra V, Novara G, Rosen RC, et al. Systematic review and meta-analysis of studies reporting urinary continence recovery after robot-assisted radical prostatectomy. Review. European Urology. 2012;62(3):405-417. doi:10.1016/j.eururo.2012.05.045

26. Frota R, Turna B, Barros R, Gill IS. Comparison of radical prostatectomy techniques: open, laparoscopic and robotic assisted. Int Braz J Urol. 2008 May-Jun 2008;34(3):259-68; discussion 268-9.

27. García-Perdomo HA, Correa-Ochoa JJ, Contreras-García R, Daneshmand S. Effectiveness of extended pelvic lymphadenectomy in the survival of prostate cancer: a systematic review and meta-analysis. Central European Journal of Urology. 2018;71(3):262-269. doi:http://dx.doi.org/10.5173/ceju.2018.1703

28. Grasso AAC, Mistretta FA, Sandri M, et al. Posterior musculofascial reconstruction after radical prostatectomy: an updated systematic review and a meta-analysis. Review. BJU International. 2016;118(1):20-34. doi:10.1111/bju.13480

29. Haifler M, Benjamin B, Ghinea R, Avital S. The impact of previous laparoscopic inguinal hernia repair on radical prostatectomy. J Endourol. Nov 2012;26(11):1458-62. doi:10.1089/end.2012.0285

30. Heer R, Raymond I, Jackson MJ, Soomro NA. A critical systematic review of recent clinical trials comparing open retropubic, laparoscopic and robot-assisted laparoscopic radical prostatectomy. Article. Reviews on Recent Clinical Trials. 2011;6(3):241-249. doi:10.2174/157488711796575513

31. Huang X, Wang L, Zheng X, Wang X. Comparison of perioperative, functional, and oncologic outcomes between standard laparoscopic and robotic-assisted radical prostatectomy: a systemic review and meta-analysis. Surg Endosc. Mar 2017;31(3):1045-1060. doi:10.1007/s00464-016-5125-1

32. Ilic D, Evans SM, Allan CA, Jung JH, Murphy D, Frydenberg M. Laparoscopic and robot-assisted vs open radical prostatectomy for the treatment of localized prostate cancer: a Cochrane systematic review. Review. BJU International. 2018;121(6):845-853. doi:10.1111/bju.14062

33. Kallidonis P, Rai BP, Qazi H, et al. Critical appraisal of literature comparing minimally invasive extraperitoneal and transperitoneal radical prostatectomy: A systematic review and meta-analysis. Arab J Urol. Dec 2017;15(4):267-279. doi:10.1016/j.aju.2017.07.003

34. Kang DC, Hardee MJ, Fesperman SF, Stoffs TL, Dahm P. Low Quality of Evidence for Robot-Assisted Laparoscopic Prostatectomy: Results of a Systematic Review of the Published Literature. Article. European Urology. 2010;57(6):930-937. doi:10.1016/j.eururo.2010.01.034

35. Kilminster S, Müller S, Menon M, Joseph JV, Ralph DJ, Patel HR. Predicting erectile function outcome in men after radical prostatectomy for prostate cancer. BJU Int. Aug 2012;110(3):422-6. doi:10.1111/j.1464-410X.2011.10757.x

36. Kim JW, Kim DK, Ahn HK, Jung HD, Lee JY, Cho KS. Effect of Bladder Neck Preservation on Long-Term Urinary Continence after Robot-Assisted Laparoscopic Prostatectomy: A Systematic Review and Meta-Analysis. J Clin Med. Nov 2019;8(12)doi:10.3390/jcm8122068

37. Kowalewski KF, Tapking C, Hetjens S, et al. Interrupted versus Continuous Suturing for Vesicourethral Anastomosis During Radical Prostatectomy: A Systematic Review and Meta-analysis. Eur Urol Focus. Nov 2019;5(6):980-991. doi:10.1016/j.euf.2018.05.009

38. Lee SH, Seo HJ, Lee NR, Son SK, Kim DK, Rha KH. Robot-assisted radical prostatectomy has lower biochemical recurrence than laparoscopic radical prostatectomy: Systematic review and meta-analysis. Review. Investigative and Clinical Urology. 2017;58(3):152-163. doi:10.4111/icu.2017.58.3.152

39. Leow JJ, Leong EK, Serrell EC, et al. Systematic Review of the Volume–Outcome Relationship for Radical Prostatectomy. Review. European Urology Focus. 2018;4(6):775-789. doi:10.1016/j.euf.2017.03.008

40. Li HX, Liu CX, Zhang HB, et al. The Use of Unidirectional Barbed Suture for Urethrovesical Anastomosis during Robot-Assisted Radical Prostatectomy: A Systematic Review and Meta-Analysis of Efficacy and Safety. Article. Plos One. Jul 2015;10(7)doi:10.1371/journal.pone.0131167

41. Li J, Jiang Q, Li Q, Zhang Y, Gao L. Does time interval between prostate biopsy and surgery affect outcomes of radical prostatectomy? A systematic review and meta-analysis. Int Urol Nephrol. Nov 2019;doi:10.1007/s11255-019-02344-6

42. Li MX, Cheng P, Yao L, et al. Suprapubic tube compared with urethral catheter drainage after robot-assisted radical prostatectomy: A systematic review and meta-analysis. Article. Asian journal of surgery. 2019;42(1):71-80. doi:10.1016/j.asjsur.2018.08.004

43. Lim SK, Kim KH, Shin TY, Rha KH. Current status of robot-assisted laparoscopic radical prostatectomy: how does it compare with other surgical approaches? Int J Urol. Mar 2013;20(3):271-84. doi:10.1111/j.1442-2042.2012.03193.x

44. Lin YF, Lai SK, Liu QY, et al. Efficacy and safety of barbed suture in minimally invasive radical prostatectomy: A systematic review and meta-analysis. Review. Kaohsiung Journal of Medical Sciences. Mar 2017;33(3):107-115. doi:10.1016/j.kjms.2016.12.005

45. Marra AR, Puig-Asensio M, Edmond MB, Schweizer ML, Nepple KG. Infectious Complications of Conventional Laparoscopic vs Robotic Laparoscopic Prostatectomy: A Systematic Literature Review and Meta-Analysis. J Endourol. 03 2019;33(3):179-188. doi:10.1089/end.2018.0815

46. Mochtar CA, Kauer PC, Laguna MP, de la Rosette JJ. Urinary leakage after laparoscopic radical prostatectomy: a systematic review. J Endourol. Nov 2007;21(11):1371-9. doi:10.1089/end.2006.9979

47. Moran PS, O'Neill M, Teljeur C, et al. Robot-assisted radical prostatectomy compared with open and laparoscopic approaches: A systematic review and meta-analysis. Review. International Journal of Urology. 2013;20(3):312-321. doi:10.1111/iju.12070

48. Mungovan SF, Sandhu JS, Akin O, Smart NA, Graham PL, Patel MI. Preoperative Membranous Urethral Length Measurement and Continence Recovery Following Radical Prostatectomy: A Systematic Review and Meta-analysis. Eur Urol. Mar 2017;71(3):368-78. doi:10.1016/j.eururo.2016.06.023

49. Novara G, Ficarra V, Mocellin S, et al. Systematic review and meta-analysis of studies reporting oncologic outcome after robot-assisted radical prostatectomy. Review. European Urology. 2012;62(3):382-404. doi:10.1016/j.eururo.2012.05.047

50. Novara G, Ficarra V, Rosen RC, et al. Systematic review and meta-analysis of perioperative outcomes and complications after robot-assisted radical prostatectomy. Review. European Urology. 2012;62(3):431-452. doi:10.1016/j.eururo.2012.05.044

51. O'Callaghan ME, Raymond E, Campbell J, et al. Tools for predicting patient-reported outcomes in prostate cancer patients undergoing radical prostatectomy: a systematic review of prognostic accuracy and validity. Prostate Cancer and Prostatic Diseases. 2017;20(4):378-388. doi:http://dx.doi.org/10.1038/pcan.2017.28

52. Pan XW, Cui XM, Teng JF, et al. Robot-Assisted Radical Prostatectomy vs. Open Retropubic Radical Prostatectomy for Prostate Cancer: A Systematic Review and Meta-analysis. Review. Indian Journal of Surgery. Dec 2015;77:S1326-S1333. doi:10.1007/s12262-014-1170-y

53. Parsons JK, Bennett JL. Outcomes of retropubic, laparoscopic, and robotic-assisted prostatectomy. Urology. Aug 2008;72(2):412-6. doi:10.1016/j.urology.2007.11.026

54. Phukan C, Mclean A, Nambiar A, et al. Retzius sparing robotic assisted radical prostatectomy vs. conventional robotic assisted radical prostatectomy: a systematic review and meta-analysis. World J Urol. May 2019;doi:10.1007/s00345-019-02798-4

55. Picozzi SC, Ricci C, Bonavina L, et al. Feasibility and outcomes regarding open and laparoscopic radical prostatectomy in patients with previous synthetic mesh inguinal hernia repair: meta-analysis and systematic review of 7,497 patients. World J Urol. Jan 2015;33(1):59-67. doi:10.1007/s00345-014-1282-9

56. Ploussard G, Briganti A, De La Taille A, et al. Pelvic lymph node dissection during robot-assisted radical prostatectomy: Efficacy, limitations, and complications - A systematic review of the literature. Review. European Urology. 2014;65(1):7-16. doi:10.1016/j.eururo.2013.03.057

57. Ramsay C, Pickard R, Robertson C, et al. Systematic review and economic modelling of the relative clinical benefit and cost-effectiveness of laparoscopic surgery and robotic surgery for removal of the prostate in men with localised prostate cancer. Health Technol Assess. 2012;16(41):1-313. doi:10.3310/hta16410

58. Rassweiler J, Hruza M, Teber D, Su LM. Laparoscopic and robotic assisted radical prostatectomy--critical analysis of the results. Eur Urol. Apr 2006;49(4):612-24. doi:10.1016/j.eururo.2005.12.054

59. Reeves F, Preece P, Kapoor J, et al. Preservation of the Neurovascular Bundles Is Associated with Improved Time to Continence After Radical Prostatectomy But Not Long-term Continence Rates: Results of a Systematic Review and Meta-analysis. Review. European Urology. Oct 2015;68(4):692-704. doi:10.1016/j.eururo.2014.10.020

60. Robertson C, Close A, Fraser C, et al. Relative effectiveness of robot-assisted and standard laparoscopic prostatectomy as alternatives to open radical prostatectomy for treatment of localised prostate cancer: A systematic review and mixed treatment comparison meta-analysis. Article. BJU International. 2013;112(6):798-812. doi:10.1111/bju.12247

61. Rocco B, Cozzi G, Spinelli MG, et al. Posterior musculofascial reconstruction after radical prostatectomy: A systematic review of the literature. Article. European Urology. 2012;62(5):779-790. doi:10.1016/j.eururo.2012.05.041

62. Sandoval Salinas C, González Rangel AL, Cataño Cataño JG, Fuentes Pachón JC, Castillo Londoño JS. Efficacy of Robotic-Assisted Prostatectomy in Localized Prostate Cancer: A Systematic Review of Clinical Trials. Adv Urol. 2013;2013doi:10.1155/2013/105651

63. Seo HJ, Lee NR, Son SK, Kim DK, Rha KH, Lee SH. Comparison of robot-assisted radical prostatectomy and open radical prostatectomy outcomes: A systematic review and meta-analysis. Article. Yonsei Medical Journal. 2016;57(5):1165-1177. doi:10.3349/ymj.2016.57.5.1165

64. Srougi V, Bessa J, Baghdadi M, et al. Surgical method influences specimen margins and biochemical recurrence during radical prostatectomy for high-risk prostate cancer: a systematic review and meta-analysis. Review. World journal of urology. 2017;35(10):1481-1488. doi:10.1007/s00345-017-2021-9

65. Steffens D, Thanigasalam R, Leslie S, Maneck B, Young JM, Solomon M. Robotic Surgery in Uro-oncology: A Systematic Review and Meta-analysis of Randomized Controlled Trials. Urology. 2017;106(1):9-17. doi:10.1016/j.urology.2017.03.015

66. Tai TE, Wu CC, Kang YN, Wu JC. Effects of Retzius sparing on robot-assisted laparoscopic prostatectomy: a systematic review with meta-analysis. Surg Endosc. Oct 2019;doi:10.1007/s00464-019-07190-2

67. Tal R, Alphs HH, Krebs P, Nelson CJ, Mulhall JP. Erectile function recovery rate after radical prostatectomy: a meta-analysis. J Sex Med. Sep 2009;6(9):2538-46. doi:10.1111/j.1743-6109.2009.01351.x

68. Tan A, Ashrafian H, Scott AJ, et al. Robotic surgery: disruptive innovation or unfulfilled promise? A systematic review and meta-analysis of the first 30 years. Review. Surgical Endoscopy and Other Interventional Techniques. Oct 2016;30(10):4330-4352. doi:10.1007/s00464-016-4752-x

69. Tewari A, Sooriakumaran P, Bloch DA, Seshadri-Kreaden U, Hebert AE, Wiklund P. Positive surgical margin and perioperative complication rates of primary surgical treatments for prostate cancer: A systematic review and meta-analysis comparing retropubic, laparoscopic, and robotic prostatectomy. Review. European Urology. 2012;62(1):1-15. doi:10.1016/j.eururo.2012.02.029

70. Tooher R, Swindle P, Woo H, Miller J, Maddern G. Laparoscopic radical prostatectomy for localized prostate cancer: a systematic review of comparative studies. J Urol. Jun 2006;175(6):2011-7. doi:10.1016/S0022-5347(06)00265-5

71. Touijer K, Guillonneau B. Laparoscopic radical prostatectomy: a critical analysis of surgical quality. Eur Urol. Apr 2006;49(4):625-32. doi:10.1016/j.eururo.2006.01.018

72. Trinh QD, Bjartell A, Freedland SJ, et al. A Systematic Review of the Volume–Outcome Relationship for Radical Prostatectomy. Eur Urol. Nov 2013;64(5):786-98. doi:10.1016/j.eururo.2013.04.012

73. Veccia A, Antonelli A, Francavilla S, et al. Minimally Invasive Radical Prostatectomy after Previous Bladder Outlet Surgery: A Systematic Review and Pooled Analysis of Comparative Studies. J Urol. 09 2019;202(3):511-517. doi:10.1097/JU.0000000000000312

74. Wang L, Wang B, Ai Q, et al. Long-term cancer control outcomes of robot-assisted radical prostatectomy for prostate cancer treatment: a meta-analysis. Int Urol Nephrol. 2017/02 2017;49(6):995-1005.

75. Weng H, Zeng XT, Li S, et al. Intrafascial versus interfascial nerve sparing in radical prostatectomy for localized prostate cancer: a systematic review and meta-analysis. Article. Scientific Reports. Sep 2017;7doi:10.1038/s41598-017-11878-7

76. Whiting PF, Moore THM, Jameson CM, et al. Symptomatic and quality-of-life outcomes after treatment for clinically localised prostate cancer: a systematic review. Review. Bju International. Aug 2016;118(2):193-204. doi:10.1111/bju.13499

77. Wilt TJ, MacDonald R, Rutks I, Shamilyan TA, Taylor BC, Kane RL. Systematic review: Comparative effectiveness and harms of treatments for clinically localized prostate cancer. Review. Annals of Internal Medicine. Mar 2008;148(6):435-448. doi:10.7326/0003-4819-148-6-200803180-00209

78. Yossepowitch O, Bjartell A, Eastham JA, et al. Positive surgical margins in radical prostatectomy: outlining the problem and its long-term consequences. Eur Urol. Jan 2009;55(1):87-99. doi:10.1016/j.eururo.2008.09.051

79. Yossepowitch O, Briganti A, Eastham JA, et al. Positive surgical margins after radical prostatectomy: A systematic review and contemporary update. Review. European Urology. 2014;65(2):303-313. doi:10.1016/j.eururo.2013.07.039

80. Yuh B, Artibani W, Heidenreich A, et al. The role of robot-assisted radical prostatectomy and pelvic lymph node dissection in the management of high-risk prostate cancer: A systematic review. Review. European Urology. 2014;65(5):918-927. doi:10.1016/j.eururo.2013.05.026
