# Supplementary material for: Surgical Results and Complications for Open, Laparoscopic, and Robot-assisted Radical Prostatectomy: A Reverse Systematic Review
Source: Eur Urol Open Sci. 2022 Sep 8;44:150–61. doi: 10.1016/j.euros.2022.08.015 (PMC9468352; doi:10.1016/j.euros.2022.08.015)
Supplement: Supplementary Appendix D [file mmc4.docx]

**APPENDIX D - INCLUDED STUDIES**

Selected studies for analysis are listed below (C^1-910^):

1. Abbou CC, Hoznek A, Salomon L, et al. Laparoscopic radical prostatectomy with a remote controlled robot. J Urol. Jun 2001;165(6 Pt 1):1964-6. doi:10.1097/00005392-200106000-00027.

2. Abbou CC, Salomon L, Hoznek A, et al. Laparoscopic radical prostatectomy: preliminary results. Urology. May 2000;55(5):630-4.

3. Abdel Raheem A, Kim DK, Santok GD, et al. Stratified analysis of 800 Asian patients after robot-assisted radical prostatectomy with a median 64 months of follow up. Int J Urol. 09 2016;23(9):765-74. doi:10.1111/iju.13151

4. Abdollah F, Budäus L, Sun M, et al. Impact of caseload on total hospital charges: a direct comparison between minimally invasive and open radical prostatectomy--a population based study. J Urol. Mar 2011;185(3):855-61. doi:10.1016/j.juro.2010.10.051

5. Abdollah F, Dalela D, Sood A, et al. Intermediate-term cancer control outcomes in prostate cancer patients treated with robotic-assisted laparoscopic radical prostatectomy: a multi-institutional analysis. World J Urol. Oct 2016;34(10):1357-66. doi:10.1007/s00345-016-1781-y

6. Abdollah F, Sun M, Suardi N, et al. Prediction of functional outcomes after nerve-sparing radical prostatectomy: results of conditional survival analyses. Eur Urol. Jul 2012;62(1):42-52. doi:10.1016/j.eururo.2012.02.057

7. Abdollah F, Sun M, Suardi N, et al. A novel tool to assess the risk of urinary incontinence after nerve-sparing radical prostatectomy. BJU Int. May 2013;111(6):905-13. doi:10.1111/j.1464-410X.2012.11560.x

8. Abraham NE, Makarov DV, Laze J, Stefanovics E, Desai R, Lepor H. Patient centered outcomes in prostate cancer treatment: predictors of satisfaction up to 2 years after open radical retropubic prostatectomy. J Urol. Nov 2010;184(5):1977-81. doi:10.1016/j.juro.2010.06.099

9. Adam C, Salomon G, Walther S, et al. Photodynamic diagnosis using 5-aminolevulinic acid for the detection of positive surgical margins during radical prostatectomy in patients with carcinoma of the prostate: a multicentre, prospective, phase 2 trial of a diagnostic procedure. Eur Urol. Jun 2009;55(6):1281-8. doi:10.1016/j.eururo.2009.02.027

10. Afzal MZ, Tobert CM, Bulica E, Noyes SL, Lane BR. Modification of Technique for Suprapubic Catheter Placement After Robot-assisted Radical Prostatectomy Reduces Catheter-associated Complications. Urology. Aug 2015;86(2):401-6. doi:10.1016/j.urology.2015.02.078

11. Agarwal PK, Sammon J, Bhandari A, et al. Safety profile of robot-assisted radical prostatectomy: a standardized report of complications in 3317 patients. Eur Urol. May 2011;59(5):684-98. doi:10.1016/j.eururo.2011.01.045

12. Ahlering TE, Eichel L, Chou D, Skarecky DW. Feasibility study for robotic radical prostatectomy cautery-free neurovascular bundle preservation. Urology. May 2005;65(5):994-7. doi:10.1016/j.urology.2004.11.023

13. Ahlering TE, Eichel L, Edwards RA, Lee DI, Skarecky DW. Robotic radical prostatectomy: a technique to reduce pT2 positive margins. Urology. Dec 2004;64(6):1224-8. doi:10.1016/j.urology.2004.08.021

14. Ahlering TE, Eichel L, Edwards R, Skarecky DW. Impact of obesity on clinical outcomes in robotic prostatectomy. Urology. Apr 2005;65(4):740-4. doi:10.1016/j.urology.2004.10.061

15. Ahlering TE, Eichel L, Skarecky D. Rapid communication: early potency outcomes with cautery-free neurovascular bundle preservation with robotic laparoscopic radical prostatectomy. J Endourol. 2005 Jul-Aug 2005;19(6):715-8. doi:10.1089/end.2005.19.715

16. Ahlering TE, Eichel L, Skarecky D. Evaluation of long-term thermal injury using cautery during nerve sparing robotic prostatectomy. Urology. Dec 2008;72(6):1371-4. doi:10.1016/j.urology.2007.11.101

17. Ahlering TE, Kaplan AG, Yee DS, Skarecky DW. Prostate weight and early potency in robot-assisted radical prostatectomy. Urology. Dec 2008;72(6):1263-8. doi:10.1016/j.urology.2008.05.055

18. Ahlering TE, Rodriguez E, Skarecky DW. Overcoming obstacles: nerve-sparing issues in radical prostatectomy. J Endourol. Apr 2008;22(4):745-50. doi:10.1089/end.2007.9834

19. Ahlering TE, Skarecky D, Lee D, Clayman RV. Successful transfer of open surgical skills to a laparoscopic environment using a robotic interface: initial experience with laparoscopic radical prostatectomy. J Urol. Nov 2003;170(5):1738-41. doi:10.1097/01.ju.0000092881.24608.5e

20. Ahlering TE, Woo D, Eichel L, Lee DI, Edwards R, Skarecky DW. Robot-assisted versus open radical prostatectomy: a comparison of one surgeon's outcomes. Urology. May 2004;63(5):819-22. doi:10.1016/j.urology.2004.01.038

21. Akand M, Celik O, Avci E, Duman I, Erdogru T. Open, laparoscopic and robot-assisted laparoscopic radical prostatectomy: comparative analysis of operative and pathologic outcomes for three techniques with a single surgeon's experience. Eur Rev Med Pharmacol Sci. Feb 2015;19(4):525-31.

22. Al-Shaiji TF, Kanaroglou N, Thom A, et al. A cost-analysis comparison of laparoscopic radical prostatectomy versus open radical prostatectomy: the McMaster Institute of Urology experience. Can Urol Assoc J. Aug 2010;4(4):237-41. doi:10.5489/cuaj.09166

23. Albers P, Schäfers S, Löhmer H, de Geeter P. Seminal vesicle-sparing perineal radical prostatectomy improves early functional results in patients with low-risk prostate cancer. BJU Int. Nov 2007;100(5):1050-4. doi:10.1111/j.1464-410X.2007.07123.x

24. Albisinni S, Aoun F, LE Dinh D, et al. Comparing conventional laparoscopic to robotic-assisted extended pelvic lymph node dissection in men with intermediate and high-risk prostate cancer: a matched-pair analysis. Minerva Urol Nefrol. 02 2017;69(1):101-107. doi:10.23736/S0393-2249.16.02799-5

25. Alemozaffar M, Duclos A, Hevelone ND, et al. Technical refinement and learning curve for attenuating neurapraxia during robotic-assisted radical prostatectomy to improve sexual function. Eur Urol. Jun 2012;61(6):1222-8. doi:10.1016/j.eururo.2012.02.053

26. Alessandro S, Alessandro G, Susanna C, et al. Laparoscopic versus open radical prostatectomy in high prostate volume cases: impact on oncological and functional results. Int Braz J Urol. 2016 Mar-Apr 2016;42(2):223-33.

27. Alibhai SM, Leach M, Tomlinson G. Impact of hospital and surgeon volume on mortality and complications after prostatectomy. J Urol. Jul 2008;180(1):155-62; discussion 162-3. doi:10.1016/j.juro.2008.03.040

28. Allaf ME, Palapattu GS, Trock BJ, Carter HB, Walsh PC. Anatomical extent of lymph node dissection: impact on men with clinically localized prostate cancer. J Urol. Nov 2004;172(5 Pt 1):1840-4. doi:10.1097/01.ju.0000140912.45821.1d

29. Allaparthi SB, Hoang T, Dhanani NN, Tuerk IA. Significance of prostate weight on peri and postoperative outcomes of robot assisted laparoscopic extraperitoneal radical prostatectomy. Can J Urol. Oct 2010;17(5):5383-9.

30. Alvin LW, Gee SH, Hong HH, et al. Oncological outcomes following robotic-assisted radical prostatectomy in a multiracial Asian population. J Robot Surg. Sep 2015;9(3):201-9. doi:10.1007/s11701-015-0516-1

31. Anastasiadis AG, Salomon L, Katz R, Hoznek A, Chopin D, Abbou CC. Radical retropubic versus laparoscopic prostatectomy: a prospective comparison of functional outcome. Urology. Aug 2003;62(2):292-7.

32. Anceschi U, Gaffi M, Molinari C, Anceschi C. Posterior reconstruction and outcomes of laparoscopic radical prostatectomy in a high-risk setting. JSLS. 2013 Oct-Dec 2013;17(4):535-42. doi:10.4293/108680813X13794522666365

33. C A, B A, R I, et al. Extraperitoneal robot-assisted radical prostatectomy: Comparison with transperitoneal technique. World J Clin Urol. Jul 24, 2013 2013;2(2):3-9. doi:10.5410/wjcu.v2.i2.3

34. Anderson JE, Chang DC, Parsons JK, Talamini MA. The first national examination of outcomes and trends in robotic surgery in the United States. J Am Coll Surg. Jul 2012;215(1):107-14; discussion 114-6. doi:10.1016/j.jamcollsurg.2012.02.005

35. Antiphon P, Hoznek A, Benyoussef A, et al. Complete solo laparoscopic radical prostatectomy: initial experience. Urology. Apr 2003;61(4):724-8; discussion 728-9.

36. Arai Y, Egawa S, Terachi T, et al. Morbidity of laparoscopic radical prostatectomy: summary of early multi-institutional experience in Japan. Int J Urol. Aug 2003;10(8):430-4.

37. Arai Y, Egawa S, Tobisu K, et al. Radical retropubic prostatectomy: time trends, morbidity and mortality in Japan. BJU Int. Feb 2000;85(3):287-94. doi:10.1046/j.1464-410x.2000.00468.x

38. Arslan M, Tuncel A, Aslan Y, Kozacioglu Z, Gunlusoy B, Atan A. Comparison of the urethrovesical anastomoses with polyglecaprone (Monocryl®) and bidirectional barbed (V-Loc 180®) running sutures in laparoscopic radical prostatectomy. Arch Ital Urol Androl. Jun 2014;86(2):90-4. doi:10.4081/aiua.2014.2.90

39. Artibani W, Fracalanza S, Cavalleri S, et al. Learning curve and preliminary experience with da Vinci-assisted laparoscopic radical prostatectomy. Urol Int. 2008;80(3):237-44. doi:10.1159/000127333

40. Artibani W, Grosso G, Novara G, et al. Is laparoscopic radical prostatectomy better than traditional retropubic radical prostatectomy? An analysis of peri-operative morbidity in two contemporary series in Italy. Eur Urol. Oct 2003;44(4):401-6.

41. Asawabharuj K, Ramart P, Nualyong C, et al. Comparison of urinary continence outcome between robotic assisted laparoscopic prostatectomy versus laparoscopic radical prostatectomy. J Med Assoc Thai. Apr 2014;97(4):393-8.

42. Asimakopoulos AD, Annino F, D'Orazio A, et al. Complete periprostatic anatomy preservation during robot-assisted laparoscopic radical prostatectomy (RALP): the new pubovesical complex-sparing technique. Eur Urol. Sep 2010;58(3):407-17. doi:10.1016/j.eururo.2010.04.032

43. Asimakopoulos AD, Miano R, Di Lorenzo N, Spera E, Vespasiani G, Mugnier C. Laparoscopic versus robot-assisted bilateral nerve-sparing radical prostatectomy: comparison of pentafecta rates for a single surgeon. Surg Endosc. Nov 2013;27(11):4297-304. doi:10.1007/s00464-013-3046-9

44. Asimakopoulos AD, Pereira Fraga CT, Annino F, Pasqualetti P, Calado AA, Mugnier C. Randomized comparison between laparoscopic and robot-assisted nerve-sparing radical prostatectomy. J Sex Med. May 2011;8(5):1503-12. doi:10.1111/j.1743-6109.2011.02215.x

45. Asimakopoulos AD, Topazio L, De Angelis M, et al. Retzius-sparing versus standard robot-assisted radical prostatectomy: a prospective randomized comparison on immediate continence rates. Surg Endosc. 07 2019;33(7):2187-2196. doi:10.1007/s00464-018-6499-z

46. Atug F, Castle EP, Srivastav SK, Burgess SV, Thomas R, Davis R. Positive surgical margins in robotic-assisted radical prostatectomy: impact of learning curve on oncologic outcomes. Eur Urol. May 2006;49(5):866-71; discussion 871-2. doi:10.1016/j.eururo.2006.02.054

47. Atug F, Castle EP, Srivastav SK, Burgess SV, Thomas R, Davis R. Prospective evaluation of concomitant lymphadenectomy in robot-assisted radical prostatectomy: preliminary analysis of outcomes. J Endourol. Jul 2006;20(7):514-8. doi:10.1089/end.2006.20.514

48. Atug F, Castle EP, Woods M, Srivastav SK, Thomas R, Davis R. Transperitoneal versus extraperitoneal robotic-assisted radical prostatectomy: is one better than the other? Urology. Nov 2006;68(5):1077-81. doi:10.1016/j.urology.2006.07.008

49. Atug F, Kural AR, Tufek I, Srivastav S, Akpinar H. Anterior and posterior reconstruction technique and its impact on early return of continence after robot-assisted radical prostatectomy. J Endourol. Apr 2012;26(4):381-6. doi:10.1089/end.2010.0654

50. Augustin H, Hammerer P, Graefen M, et al. Intraoperative and perioperative morbidity of contemporary radical retropubic prostatectomy in a consecutive series of 1243 patients: results of a single center between 1999 and 2002. Eur Urol. Feb 2003;43(2):113-8. doi:10.1016/s0302-2838(02)00495-5

51. Augustin H, Pummer K, Daghofer F, Habermann H, Primus G, Hubmer G. Patient self-reporting questionnaire on urological morbidity and bother after radical retropubic prostatectomy. Eur Urol. Aug 2002;42(2):112-17. doi:10.1016/s0302-2838(02)00259-2

52. Awad H, Santilli S, Ohr M, et al. The effects of steep trendelenburg positioning on intraocular pressure during robotic radical prostatectomy. Anesth Analg. Aug 2009;109(2):473-8. doi:10.1213/ane.0b013e3181a9098f

53. Ayyathurai R, Manoharan M, Nieder AM, Kava B, Soloway MS. Factors affecting erectile function after radical retropubic prostatectomy: results from 1620 consecutive patients. BJU Int. Apr 2008;101(7):833-6. doi:10.1111/j.1464-410X.2007.07409.x

54. Badani KK, Kaul S, Menon M. Evolution of robotic radical prostatectomy: assessment after 2766 procedures. Cancer. Nov 2007;110(9):1951-8. doi:10.1002/cncr.23027

55. Ball AJ, Gambill B, Fabrizio MD, et al. Prospective longitudinal comparative study of early health-related quality-of-life outcomes in patients undergoing surgical treatment for localized prostate cancer: a short-term evaluation of five approaches from a single institution. J Endourol. Oct 2006;20(10):723-31. doi:10.1089/end.2006.20.723

56. Barbosa HoN, Siqueira TM, Barreto F, Menezes LG, Luna MJ, Calado AA. 4-Ports endoscopic extraperitoneal radical prostatectomy: preliminary and learning curve results. Int Braz J Urol. 2016 May-Jun 2016;42(3):438-48.

57. Barnoiu OS, Baron Lopez F, Garcia Galisteo E, et al. Comprehensive prediction model of urinary incontinence one year following robot-assisted radical prostatectomy. Urol Int. 2013;90(1):31-5. doi:10.1159/000343735

58. Barnoiu OS, Garcia Galisteo E, Baron Lopez F, et al. Prospective urodynamic model for prediction of urinary incontinence after robot-assisted radical prostatectomy. Urol Int. 2014;92(3):306-9. doi:10.1159/000354352

59. Barocas DA, Salem S, Kordan Y, et al. Robotic assisted laparoscopic prostatectomy versus radical retropubic prostatectomy for clinically localized prostate cancer: comparison of short-term biochemical recurrence-free survival. J Urol. Mar 2010;183(3):990-6. doi:10.1016/j.juro.2009.11.017

60. Barré C. Open radical retropubic prostatectomy. Eur Urol. Jul 2007;52(1):71-80. doi:10.1016/j.eururo.2006.11.057

61. Barry MJ, Gallagher PM, Skinner JS, Fowler FJ. Adverse effects of robotic-assisted laparoscopic versus open retropubic radical prostatectomy among a nationwide random sample of medicare-age men. J Clin Oncol. Feb 2012;30(5):513-8. doi:10.1200/JCO.2011.36.8621

62. Baumert H, Fromont G, Adorno Rosa J, Cahill D, Cathelineau X, Vallancien G. Impact of learning curve in laparoscopic radical prostatectomy on margin status: prospective study of first 100 procedures performed by one surgeon. J Endourol. Mar 2004;18(2):173-6. doi:10.1089/089277904322959824

63. Begg CB, Riedel ER, Bach PB, et al. Variations in morbidity after radical prostatectomy. N Engl J Med. Apr 2002;346(15):1138-44. doi:10.1056/NEJMsa011788

64. Bentas W, Wolfram M, Jones J, Bräutigam R, Kramer W, Binder J. Robotic technology and the translation of open radical prostatectomy to laparoscopy: the early Frankfurt experience with robotic radical prostatectomy and one year follow-up. Eur Urol. Aug 2003;44(2):175-81. doi:10.1016/s0302-2838(03)00256-2

65. Berge V, Berg RE, Hoff JR, et al. A prospective study of transition from laparoscopic to robot-assisted radical prostatectomy: quality of life outcomes after 36-month follow-up. Urology. Apr 2013;81(4):781-6. doi:10.1016/j.urology.2013.01.017

66. Bhandari A, McIntire L, Kaul SA, Hemal AK, Peabody JO, Menon M. Perioperative complications of robotic radical prostatectomy after the learning curve. J Urol. Sep 2005;174(3):915-8. doi:10.1097/01.ju.0000169458.96014.f8

67. Bhayani SB, Pavlovich CP, Hsu TS, Sullivan W, Su L. Prospective comparison of short-term convalescence: laparoscopic radical prostatectomy versus open radical retropubic prostatectomy. Urology. Mar 2003;61(3):612-6.

68. Bianco FJ, Grignon DJ, Sakr WA, et al. Radical prostatectomy with bladder neck preservation: impact of a positive margin. Eur Urol. May 2003;43(5):461-6. doi:10.1016/s0302-2838(03)00103-9

69. Bianco FJ, Scardino PT, Eastham JA. Radical prostatectomy: long-term cancer control and recovery of sexual and urinary function ("trifecta"). Urology. Nov 2005;66(5 Suppl):83-94. doi:10.1016/j.urology.2005.06.116

70. Bickert D, Frickel D. Laparoscopic radical prostatectomy. AORN J. Apr 2002;75(4):762-6, 768-74, 777-80 passim; quiz 785-90.

71. Biki B, Mascha E, Moriarty DC, Fitzpatrick JM, Sessler DI, Buggy DJ. Anesthetic technique for radical prostatectomy surgery affects cancer recurrence: a retrospective analysis. Anesthesiology. Aug 2008;109(2):180-7. doi:10.1097/ALN.0b013e31817f5b73

72. Billia M, Elhage O, Challacombe B, et al. Oncological outcomes of robotic-assisted radical prostatectomy after more than 5 years. World J Urol. Apr 2014;32(2):413-8. doi:10.1007/s00345-013-1120-5

73. Binder J, Kramer W. Robotically-assisted laparoscopic radical prostatectomy. BJU Int. Mar 2001;87(4):408-10. doi:10.1046/j.1464-410x.2001.00115.x

74. Bivalacqua TJ, Pierorazio PM, Gorin MA, Allaf ME, Carter HB, Walsh PC. Anatomic extent of pelvic lymph node dissection: impact on long-term cancer-specific outcomes in men with positive lymph nodes at time of radical prostatectomy. Urology. Sep 2013;82(3):653-8. doi:10.1016/j.urology.2013.03.086

75. Bivalacqua TJ, Schaeffer EM, Alphs H, et al. Intraperitoneal effects of extraperitoneal laparoscopic radical prostatectomy. Urology. Aug 2008;72(2):273-7. doi:10.1016/j.urology.2007.12.040

76. Blana A, Straub M, Wild PJ, et al. Approach to endoscopic extraperitoneal radical prostatectomy (EERPE): the impact of previous laparoscopic experience on the learning curve. BMC Urol. Jul 2007;7:11. doi:10.1186/1471-2490-7-11

77. Boczko J, Erturk E, Golijanin D, Madeb R, Patel H, Joseph JV. Impact of prostate size in robot-assisted radical prostatectomy. J Endourol. Feb 2007;21(2):184-8. doi:10.1089/end.2006.0163

78. Boczko J, Erturk E, Joseph JV. Is there a proper pelvic size for an extraperitoneal robot-assisted radical prostatectomy? J Endourol. Nov 2007;21(11):1353-6. doi:10.1089/end.2007.9898

79. Bolenz C, Gupta A, Hotze T, et al. Cost comparison of robotic, laparoscopic, and open radical prostatectomy for prostate cancer. Eur Urol. Mar 2010;57(3):453-8. doi:10.1016/j.eururo.2009.11.008

80. Bolenz C, Gupta A, Roehrborn CG, Lotan Y. Predictors of costs for robotic-assisted laparoscopic radical prostatectomy. Urol Oncol. 2011 May-Jun 2011;29(3):325-9. doi:10.1016/j.urolonc.2011.01.016

81. Bollens R, Vanden Bossche M, Roumeguere T, et al. Extraperitoneal laparoscopic radical prostatectomy. Results after 50 cases. Eur Urol. Jul 2001;40(1):65-9. doi:49750

82. Boorjian SA, Crispen PL, Carlson RE, et al. Impact of obesity on clinicopathologic outcomes after robot-assisted laparoscopic prostatectomy. J Endourol. Jul 2008;22(7):1471-6. doi:10.1089/end.2008.0056

83. Boorjian SA, Karnes RJ, Crispen PL, et al. The impact of positive surgical margins on mortality following radical prostatectomy during the prostate specific antigen era. J Urol. Mar 2010;183(3):1003-9. doi:10.1016/j.juro.2009.11.039

84. Borin JF, Skarecky DW, Narula N, Ahlering TE. Impact of urethral stump length on continence and positive surgical margins in robot-assisted laparoscopic prostatectomy. Urology. Jul 2007;70(1):173-7. doi:10.1016/j.urology.2007.03.050

85. Boris RS, Kaul SA, Sarle RC, Stricker HJ. Radical prostatectomy: a single surgeon comparison of retropubic, perineal, and robotic approaches. Can J Urol. Jun 2007;14(3):3566-70.

86. Bove P, Iacovelli V, Celestino F, De Carlo F, Vespasiani G, Finazzi Agrò E. 3D vs 2D laparoscopic radical prostatectomy in organ-confined prostate cancer: comparison of operative data and pentafecta rates: a single cohort study. BMC Urol. Feb 2015;15:12. doi:10.1186/s12894-015-0006-9

87. Braslis KG, Bowsher WG, Joyce G, Peters J, Costello AJ. Evolving experience with radical prostatectomy. Br J Urol. Sep 1993;72(3):341-8. doi:10.1111/j.1464-410x.1993.tb00730.x

88. Breyer BN, Davis CB, Cowan JE, Kane CJ, Carroll PR. Incidence of bladder neck contracture after robot-assisted laparoscopic and open radical prostatectomy. BJU Int. Dec 2010;106(11):1734-8. doi:10.1111/j.1464-410X.2010.09333.x

89. Brien JC, Barone B, Fabrizio M, Given R. Posterior reconstruction before vesicourethral anastomosis in patients undergoing robot-assisted laparoscopic prostatectomy leads to earlier return to baseline continence. J Endourol. Mar 2011;25(3):441-5. doi:10.1089/end.2010.0251

90. Briganti A, Capitanio U, Chun FK, et al. Impact of surgical volume on the rate of lymph node metastases in patients undergoing radical prostatectomy and extended pelvic lymph node dissection for clinically localized prostate cancer. Eur Urol. Oct 2008;54(4):794-802. doi:10.1016/j.eururo.2008.05.018

91. Briganti A, Gallina A, Suardi N, et al. Predicting erectile function recovery after bilateral nerve sparing radical prostatectomy: a proposal of a novel preoperative risk stratification. J Sex Med. Jul 2010;7(7):2521-31. doi:10.1111/j.1743-6109.2010.01845.x

92. Brown JA, Garlitz C, Gomella LG, et al. Pathologic comparison of laparoscopic versus open radical retropubic prostatectomy specimens. Urology. Sep 2003;62(3):481-6.

93. Brown JA, Garlitz C, Gomella LG, McGinnis DE, Diamond SM, Strup SE. Perioperative morbidity of laparoscopic radical prostatectomy compared with open radical retropubic prostatectomy. Urol Oncol. 2004 Mar-Apr 2004;22(2):102-6. doi:10.1016/S1078-1439(03)00101-7

94. Brown JA, Rodin DM, Harisinghani M, Dahl DM. Impact of preoperative endorectal MRI stage classification on neurovascular bundle sparing aggressiveness and the radical prostatectomy positive margin rate. Urol Oncol. 2009 Mar-Apr 2009;27(2):174-9. doi:10.1016/j.urolonc.2008.04.009

95. Brown JA, Rodin DM, Lee B, Dahl DM. Laparoscopic radical prostatectomy and body mass index: an assessment of 151 sequential cases. J Urol. Feb 2005;173(2):442-5. doi:10.1097/01.ju.0000148865.89309.cb

96. Brown JA, Rodin D, Lee B, Dahl DM. Transperitoneal versus extraperitoneal approach to laparoscopic radical prostatectomy: an assessment of 156 cases. Urology. Feb 2005;65(2):320-4. doi:10.1016/j.urology.2004.09.018

97. Brunocilla E, Schiavina R, Pultrone CV, et al. Preservation of the smooth muscular internal (vesical) sphincter and of the proximal urethra for the early recovery of urinary continence after retropubic radical prostatectomy: a prospective case-control study. Int J Urol. Feb 2014;21(2):157-62. doi:10.1111/iju.12206

98. Budäus L, Abdollah F, Sun M, et al. Annual surgical caseload and open radical prostatectomy outcomes: improving temporal trends. J Urol. Dec 2010;184(6):2285-90. doi:10.1016/j.juro.2010.08.024

99. Budäus L, Isbarn H, Schlomm T, et al. Current technique of open intrafascial nerve-sparing retropubic prostatectomy. Eur Urol. Aug 2009;56(2):317-24. doi:10.1016/j.eururo.2009.05.044

100. Burgess SV, Atug F, Castle EP, Davis R, Thomas R. Cost analysis of radical retropubic, perineal, and robotic prostatectomy. J Endourol. Oct 2006;20(10):827-30. doi:10.1089/end.2006.20.827

101. Burkhard FC, Kessler TM, Fleischmann A, Thalmann GN, Schumacher M, Studer UE. Nerve sparing open radical retropubic prostatectomy--does it have an impact on urinary continence? J Urol. Jul 2006;176(1):189-95. doi:10.1016/S0022-5347(06)00574-X

102. Busch J, Gonzalgo ML, Leva N, et al. Matched comparison of robot-assisted, laparoscopic and open radical prostatectomy regarding pathologic and oncologic outcomes in obese patients. World J Urol. Mar 2015;33(3):397-402. doi:10.1007/s00345-014-1326-1

103. Busch J, Stephan C, Herold A, et al. Long-term oncological and continence outcomes after laparoscopic radical prostatectomy: a single-centre experience. BJU Int. Dec 2012;110(11 Pt C):E985-90. doi:10.1111/j.1464-410X.2012.11279.x

104. Caballero Romeu JP, Palacios Ramos J, Pereira Arias JG, Gamarra Quintanilla M, Astobieta Odriozola A, Ibarluzea González G. [Radical prostatectomy: evaluation of learning curve outcomes laparoscopic and robotic-assisted laparoscopic techniques with radical retropubic prostatectomy]. Actas Urol Esp. 2008 Nov-Dec 2008;32(10):968-75. doi:10.1016/s0210-4806(08)73974-3

105. Campeggi A, Xylinas E, Ploussard G, et al. Impact of body mass index on perioperative morbidity, oncological, and functional outcomes after extraperitoneal laparoscopic radical prostatectomy. Urology. Sep 2012;80(3):576-84. doi:10.1016/j.urology.2012.04.066

106. Capello SA, Boczko J, Patel HR, Joseph JV. Randomized comparison of extraperitoneal and transperitoneal access for robot-assisted radical prostatectomy. J Endourol. Oct 2007;21(10):1199-202. doi:10.1089/end.2007.9906

107. Capello SA, Patel HR, Joseph JV. Surgical case order does not affect outcomes during robot-assisted radical prostatectomy. J Robot Surg. May 2008;2(1):25-9. doi:10.1007/s11701-007-0066-2

108. Carini M, Masieri L, Minervini A, Lapini A, Serni S. Oncological and functional results of antegrade radical retropubic prostatectomy for the treatment of clinically localised prostate cancer. Eur Urol. Mar 2008;53(3):554-61. doi:10.1016/j.eururo.2007.07.004

109. Carlsson S, Nilsson AE, Schumacher MC, et al. Surgery-related complications in 1253 robot-assisted and 485 open retropubic radical prostatectomies at the Karolinska University Hospital, Sweden. Urology. May 2010;75(5):1092-7. doi:10.1016/j.urology.2009.09.075

110. Carlsson S, Nilsson A, Wiklund PN. Postoperative urinary continence after robot-assisted laparoscopic radical prostatectomy. Scand J Urol Nephrol. 2006;40(2):103-7. doi:10.1080/00365590500368120

111. Carlucci JR, Nabizada-Pace F, Samadi DB. Robot-assisted laparoscopic radical prostatectomy: technique and outcomes of 700 cases. Int J Biomed Sci. Sep 2009;5(3):201-8.

112. Carter SC, Lipsitz S, Shih YC, Nguyen PL, Trinh QD, Hu JC. Population-based determinants of radical prostatectomy operative time. BJU Int. May 2014;113(5b):E112-8. doi:10.1111/bju.12451

113. Carvalhal GF, Griffin CR, Kan D, Loeb S, Catalona WJ. Reducing blood loss in open radical retropubic prostatectomy with prophylactic periprostatic sutures. BJU Int. Jun 2010;105(12):1650-3. doi:10.1111/j.1464-410X.2009.09034.x

114. Castle EP, Atug F, Woods M, Thomas R, Davis R. Impact of body mass index on outcomes after robot assisted radical prostatectomy. World J Urol. Feb 2008;26(1):91-5. doi:10.1007/s00345-007-0217-0

115. Catalona WJ, Carvalhal GF, Mager DE, Smith DS. Potency, continence and complication rates in 1,870 consecutive radical retropubic prostatectomies. J Urol. Aug 1999;162(2):433-8.

116. Cathelineau X, Cahill D, Widmer H, Rozet F, Baumert H, Vallancien G. Transperitoneal or extraperitoneal approach for laparoscopic radical prostatectomy: a false debate over a real challenge. J Urol. Feb 2004;171(2 Pt 1):714-6. doi:10.1097/01.ju.0000103885.71434.02

117. Cathelineau X, Rozet F, Vallancien G. Robotic radical prostatectomy: the European experience. Urol Clin North Am. Nov 2004;31(4):693-9, viii. doi:10.1016/j.ucl.2004.06.001

118. Celik O, Akand M, Ekin G, Duman I, Ilbey YO, Erdogru T. Laparoscopic Radical Prostatectomy Alone or With Laparoscopic Herniorrhaphy. JSLS. 2015 Oct-Dec 2015;19(4)doi:10.4293/JSLS.2015.00090

119. Chabert CC, Merrilees DA, Neill MG, Eden CG. Curtain dissection of the lateral prostatic fascia and potency after laparoscopic radical prostatectomy: a veil of mystery. BJU Int. May 2008;101(10):1285-8. doi:10.1111/j.1464-410X.2008.07595.x

120. Chalasani V, Martinez CH, Lim D, et al. Impact of body mass index on perioperative outcomes during the learning curve for robot-assisted radical prostatectomy. Can Urol Assoc J. Aug 2010;4(4):250-4. doi:10.5489/cuaj.09083

121. Chan EO, Groome PA, Siemens DR. Validation of quality indicators for radical prostatectomy. Int J Cancer. Dec 2008;123(11):2651-7. doi:10.1002/ijc.23782

122. Chan RC, Barocas DA, Chang SS, et al. Effect of a large prostate gland on open and robotically assisted laparoscopic radical prostatectomy. BJU Int. May 2008;101(9):1140-4. doi:10.1111/j.1464-410X.2007.07428.x

123. Chandak P, Byrne N, Lynch H, et al. Three-dimensional printing in robot-assisted radical prostatectomy - an Idea, Development, Exploration, Assessment, Long-term follow-up (IDEAL) Phase 2a study. BJU Int. 09 2018;122(3):360-361. doi:10.1111/bju.14189

124. Chang IH, Byun SS, Hong SK, Lee SE. Assessing the body mass index of patients might help to predict blood loss during radical retropubic prostatectomy in Korean men. BJU Int. Mar 2007;99(3):570-4. doi:10.1111/j.1464-410X.2006.06637.x

125. Chang KD, Abdel Raheem A, Santok GDR, et al. Anatomical Retzius-space preservation is associated with lower incidence of postoperative inguinal hernia development after robot-assisted radical prostatectomy. Hernia. 08 2017;21(4):555-561. doi:10.1007/s10029-017-1588-9

126. Chang LW, Hung SC, Hu JC, Chiu KY. Retzius-sparing Robotic-assisted Radical Prostatectomy Associated with Less Bladder Neck Descent and Better Early Continence Outcome. Anticancer Res. Jan 2018;38(1):345-351. doi:10.21873/anticanres.12228

127. Cheetham PJ, Lee DJ, Rose-Morris A, Brewster SF, Badani KK. Does the presence of robotic surgery affect demographics in patients choosing to undergo radical prostatectomy? A multi-center contemporary analysis. J Robot Surg. Sep 2010;4(3):155-60. doi:10.1007/s11701-010-0200-4

128. Chen MK, Luo Y, Zhang H, et al. Laparoscopic radical prostatectomy plus extended lymph nodes dissection for cases with non-extra node metastatic prostate cancer: 5-year experience in a single Chinese institution. J Cancer Res Clin Oncol. May 2013;139(5):871-8. doi:10.1007/s00432-013-1395-3

129. Chien GW, Mikhail AA, Orvieto MA, et al. Modified clipless antegrade nerve preservation in robotic-assisted laparoscopic radical prostatectomy with validated sexual function evaluation. Urology. Aug 2005;66(2):419-23. doi:10.1016/j.urology.2005.03.015

130. Chino J, Schroeck FR, Sun L, et al. Robot-assisted laparoscopic prostatectomy is not associated with early postoperative radiation therapy. BJU Int. Nov 2009;104(10):1496-500. doi:10.1111/j.1464-410X.2009.08588.x

131. Cho JW, Kim TH, Sung GT. Laparoscopic Radical Prostatectomy versus Robot-Assisted Laparoscopic Radical Prostatectomy: A Single Surgeon's Experience. Korean J Urol. 12/ 2009;50(12):1198-1202.

132. Choi H, Ko YH, Kang SG, et al. Biopsy related prostate status does not affect on the clinicopathological outcome of robotic assisted laparoscopic radical prostatectomy. Cancer Res Treat. Dec 2009;41(4):205-10. doi:10.4143/crt.2009.41.4.205

133. Choi SK, Park S, Ahn H. Randomized clinical trial of a bladder neck plication stitch during robot-assisted radical prostatectomy. Asian J Androl. 2015 Mar-Apr 2015;17(2):304-8. doi:10.4103/1008-682X.139258

134. Choi WW, Freire MP, Soukup JR, et al. Nerve-sparing technique and urinary control after robot-assisted laparoscopic prostatectomy. World J Urol. Feb 2011;29(1):21-7. doi:10.1007/s00345-010-0601-z

135. Choi WW, Gu X, Lipsitz SR, D'Amico AV, Williams SB, Hu JC. The effect of minimally invasive and open radical prostatectomy surgeon volume. Urol Oncol. Sep 2012;30(5):569-76. doi:10.1016/j.urolonc.2010.06.009

136. Choo MS, Choi WS, Cho SY, Ku JH, Kim HH, Kwak C. Impact of prostate volume on oncological and functional outcomes after radical prostatectomy: robot-assisted laparoscopic versus open retropubic. Korean J Urol. Jan 2013;54(1):15-21. doi:10.4111/kju.2013.54.1.15

137. Chuang MS, O'Connor RC, Laven BA, Orvieto MA, Brendler CB. Early release of the neurovascular bundles and optical loupe magnification lead to improved and earlier return of potency following radical retropubic prostatectomy. J Urol. Feb 2005;173(2):537-9. doi:10.1097/01.ju.0000148941.57203.ec

138. Chun FK, Briganti A, Antebi E, et al. Surgical volume is related to the rate of positive surgical margins at radical prostatectomy in European patients. BJU Int. Dec 2006;98(6):1204-9. doi:10.1111/j.1464-410X.2006.06442.x

139. Chun FK, Graefen M, Zacharias M, et al. Anatomic radical retropubic prostatectomy-long-term recurrence-free survival rates for localized prostate cancer. World J Urol. Aug 2006;24(3):273-80. doi:10.1007/s00345-006-0058-2

140. Chung JS, Kim WT, Ham WS, et al. Comparison of oncological results, functional outcomes, and complications for transperitoneal versus extraperitoneal robot-assisted radical prostatectomy: a single surgeon's experience. J Endourol. May 2011;25(5):787-92. doi:10.1089/end.2010.0222

141. Chłosta PL, Drewa T, Jaskulski J, Dobruch J, Varkarakis J, Borówka A. Bladder neck preservation during classic laparoscopic radical prostatectomy - point of technique and preliminary results. Wideochir Inne Tech Maloinwazyjne. Jun 2012;7(2):89-95. doi:10.5114/wiitm.2011.25981

142. Cindolo L, Salzano L, Mirone V, et al. Thromboprophylaxis in radical retropubic prostatectomy: efficacy and patient compliance of a dual modality. Urol Int. 2009;83(1):12-8. doi:10.1159/000224861

143. Coakley FV, Eberhardt S, Kattan MW, Wei DC, Scardino PT, Hricak H. Urinary continence after radical retropubic prostatectomy: relationship with membranous urethral length on preoperative endorectal magnetic resonance imaging. J Urol. Sep 2002;168(3):1032-5. doi:10.1097/01.ju.0000025881.75827.a5

144. Coelho RF, Chauhan S, Orvieto MA, Palmer KJ, Rocco B, Patel VR. Predictive factors for positive surgical margins and their locations after robot-assisted laparoscopic radical prostatectomy. Eur Urol. Jun 2010;57(6):1022-9. doi:10.1016/j.eururo.2010.01.040

145. Coelho RF, Chauhan S, Orvieto MA, et al. Influence of modified posterior reconstruction of the rhabdosphincter on early recovery of continence and anastomotic leakage rates after robot-assisted radical prostatectomy. Eur Urol. 01 2011;59(1):72-80. doi:10.1016/j.eururo.2010.08.025

146. Coelho RF, Palmer KJ, Rocco B, et al. Early complication rates in a single-surgeon series of 2500 robotic-assisted radical prostatectomies: report applying a standardized grading system. Eur Urol. Jun 2010;57(6):945-52. doi:10.1016/j.eururo.2010.02.001

147. Cohen MS, Triaca V, Silverman ML, Tuerk IA. Progression of laparoscopic radical prostatectomy: improved outcomes with the extraperitoneal approach and a running anastomosis. J Endourol. Aug 2006;20(8):574-9. doi:10.1089/end.2006.20.574

148. Connolly SS, Cathcart PJ, Gilmore P, et al. Robotic radical prostatectomy as the initial step in multimodal therapy for men with high-risk localised prostate cancer: initial experience of 160 men. BJU Int. Mar 2012;109(5):752-9. doi:10.1111/j.1464-410X.2011.10548.x

149. Constantinides CA, Tyritzis SI, Skolarikos A, Liatsikos E, Zervas A, Deliveliotis C. Short- and long-term complications of open radical prostatectomy according to the Clavien classification system. BJU Int. Feb 2009;103(3):336-40. doi:10.1111/j.1464-410X.2008.08080.x

150. Cooperberg MR, Kane CJ, Cowan JE, Carroll PR. Adequacy of lymphadenectomy among men undergoing robot-assisted laparoscopic radical prostatectomy. BJU Int. Jan 2010;105(1):88-92. doi:10.1111/j.1464-410X.2009.08699.x

151. Coronato EE, Harmon JD, Ginsberg PC, et al. A multi-institutional comparison of radical retropubic prostatectomy, radical perineal prostatectomy, and robot-assisted laparoscopic prostatectomy for treatment of localized prostate cancer. J Robot Surg. Oct 2009;3(3):175. doi:10.1007/s11701-009-0158-2

152. Costello AJ, Haxhimolla H, Crowe H, Peters JS. Installation of telerobotic surgery and initial experience with telerobotic radical prostatectomy. BJU Int. Jul 2005;96(1):34-8. doi:10.1111/j.1464-410X.2005.05562.x

153. Coughlin GD, Yaxley JW, Chambers SK, et al. Robot-assisted laparoscopic prostatectomy versus open radical retropubic prostatectomy: 24-month outcomes from a randomised controlled study. Lancet Oncol. 08 2018;19(8):1051-1060. doi:10.1016/S1470-2045(18)30357-7

154. Crocitto LE, Ly M, Satterthwaite R, Wilson T, Nelson RA. Can robotic assisted laparoscopic prostatectomy be recommended to obese patients? J Robot Surg. 2008;1(4):297-302. doi:10.1007/s11701-007-0059-1

155. Curto F, Benijts J, Pansadoro A, et al. Nerve sparing laparoscopic radical prostatectomy: our technique. Eur Urol. Feb 2006;49(2):344-52. doi:10.1016/j.eururo.2005.11.029

156. D'Alonzo RC, Gan TJ, Moul JW, et al. A retrospective comparison of anesthetic management of robot-assisted laparoscopic radical prostatectomy versus radical retropubic prostatectomy. J Clin Anesth. Aug 2009;21(5):322-8. doi:10.1016/j.jclinane.2008.09.005

157. Dahl DM, Barry MJ, McGovern FJ, Chang Y, Walker-Corkery E, McDougal WS. A prospective study of symptom distress and return to baseline function after open versus laparoscopic radical prostatectomy. J Urol. Sep 2009;182(3):956-65. doi:10.1016/j.juro.2009.05.044

158. Dahl DM, He W, Lazarus R, McDougal WS, Wu CL. Pathologic outcome of laparoscopic and open radical prostatectomy. Urology. Dec 2006;68(6):1253-6. doi:10.1016/j.urology.2006.08.1054

159. Dahl DM, L'esperance JO, Trainer AF, et al. Laparoscopic radical prostatectomy: initial 70 cases at a U.S. university medical center. Urology. Nov 2002;60(5):859-63.

160. Dalela D, Jeong W, Prasad MA, et al. A Pragmatic Randomized Controlled Trial Examining the Impact of the Retzius-sparing Approach on Early Urinary Continence Recovery After Robot-assisted Radical Prostatectomy. Eur Urol. Nov 2017;72(5):677-685. doi:10.1016/j.eururo.2017.04.029

161. Dalkin BL, Christopher BA, Shawler D. Health related quality of life outcomes after radical prostatectomy: attention to study design and the patient-based importance of single-surgeon studies. Urol Oncol. 2006 Jan-Feb 2006;24(1):28-32. doi:10.1016/j.urolonc.2005.05.009

162. Damani A, Van Hemelrijck M, Wulaningsih W, Crawley D, Cahill D. Are you now a good surgeon? T2 positive margin status as a quality outcome measure following radical prostatectomy. World J Urol. Jan 2017;35(1):35-43. doi:10.1007/s00345-016-1836-0

163. Dangle PP, Shah KK, Kaffenberger B, Patel VR. The use of high resolution optical coherence tomography to evaluate robotic radical prostatectomy specimens. Int Braz J Urol. 2009 May-Jun 2009;35(3):344-53. doi:10.1590/s1677-55382009000300011

164. Daouacher G, Waldén M. A simple reconstruction of the posterior aspect of rhabdosphincter and sparing of puboprostatic collar reduces the time to early continence after laparoscopic radical prostatectomy. J Endourol. Apr 2014;28(4):481-6. doi:10.1089/end.2013.0633

165. Dash A, Dunn RL, Resh J, Wei JT, Montie JE, Sanda MG. Patient, surgeon, and treatment characteristics associated with homologous blood transfusion requirement during radical retropubic prostatectomy: multivariate nomogram to assist patient counseling. Urology. Jul 2004;64(1):117-22. doi:10.1016/j.urology.2004.02.018

166. Davis JW, Chang DW, Chevray P, et al. Randomized phase II trial evaluation of erectile function after attempted unilateral cavernous nerve-sparing retropubic radical prostatectomy with versus without unilateral sural nerve grafting for clinically localized prostate cancer. Eur Urol. May 2009;55(5):1135-43. doi:10.1016/j.eururo.2008.08.051

167. Davis JW, Kamat A, Munsell M, Pettaway C, Pisters L, Matin S. Initial experience of teaching robot-assisted radical prostatectomy to surgeons-in-training: can training be evaluated and standardized? BJU Int. Apr 2010;105(8):1148-54. doi:10.1111/j.1464-410X.2009.08997.x

168. Deliveliotis C, Delis A, Papatsoris A, Antoniou N, Varkarakis IM. Local steroid application during nerve-sparing radical retropubic prostatectomy. BJU Int. Sep 2005;96(4):533-5. doi:10.1111/j.1464-410X.2005.05679.x

169. Deliveliotis C, Liakouras C, Delis A, Skolarikos A, Varkarakis J, Protogerou V. Prostate operations: long-term effects on sexual and urinary function and quality of life. Comparison with an age-matched control population. Urol Res. Aug 2004;32(4):283-9. doi:10.1007/s00240-004-0411-0

170. Descazeaud A, Debré B, Flam TA. Age difference between patient and partner is a predictive factor of potency rate following radical prostatectomy. J Urol. Dec 2006;176(6 Pt 1):2594-8; discussion 2598. doi:10.1016/j.juro.2006.07.145

171. Dev HS, Wiklund P, Patel V, et al. Surgical margin length and location affect recurrence rates after robotic prostatectomy. Urol Oncol. Mar 2015;33(3):109.e7-13. doi:10.1016/j.urolonc.2014.11.005

172. Di Pierro GB, Baumeister P, Stucki P, Beatrice J, Danuser H, Mattei A. A prospective trial comparing consecutive series of open retropubic and robot-assisted laparoscopic radical prostatectomy in a centre with a limited caseload. Eur Urol. Jan 2011;59(1):1-6. doi:10.1016/j.eururo.2010.10.026

173. Diaz M, Peabody JO, Kapoor V, et al. Oncologic outcomes at 10 years following robotic radical prostatectomy. Eur Urol. Jun 2015;67(6):1168-1176. doi:10.1016/j.eururo.2014.06.025

174. Dillioglugil O, Leibman BD, Leibman NS, Kattan MW, Rosas AL, Scardino PT. Risk factors for complications and morbidity after radical retropubic prostatectomy. J Urol. May 1997;157(5):1760-7.

175. Do HM, Turner K, Dietel A, Wedderburn A, Liatsikos E, Stolzenburg JU. Previous laparoscopic inguinal hernia repair does not adversely affect the functional or oncological outcomes of endoscopic extraperitoneal radical prostatectomy. Urology. Apr 2011;77(4):963-7. doi:10.1016/j.urology.2010.06.068

176. Do M, Haefner T, Liatsikos E, et al. Endoscopic extraperitoneal radical prostatectomy after previous transurethral resection of prostate: oncologic and functional outcomes of 100 cases. Urology. Jun 2010;75(6):1348-52. doi:10.1016/j.urology.2009.09.009

177. Do M, Liatsikos EN, Kallidonis P, et al. Hernia repair during endoscopic extraperitoneal radical prostatectomy: outcome after 93 cases. J Endourol. Apr 2011;25(4):625-9. doi:10.1089/end.2010.0406

178. Donnellan SM, Duncan HJ, MacGregor RJ, Russell JM. Prospective assessment of incontinence after radical retropubic prostatectomy: objective and subjective analysis. Urology. Feb 1997;49(2):225-30. doi:10.1016/S0090-4295(96)00451-7

179. Doumerc N, Yuen C, Savdie R, Rahman MB, Pe Benito R, Stricker P. Robot-assisted laparoscopic prostatectomy: analysis of an experienced open surgeon's learning curve after 300 procedures. J Robot Surg. Jan 2010;3(4):229-34. doi:10.1007/s11701-010-0171-5

180. Doumerc N, Yuen C, Savdie R, et al. Should experienced open prostatic surgeons convert to robotic surgery? The real learning curve for one surgeon over 3 years. BJU Int. Aug 2010;106(3):378-84. doi:10.1111/j.1464-410X.2009.09158.x

181. Drouin SJ, Vaessen C, Hupertan V, et al. Comparison of mid-term carcinologic control obtained after open, laparoscopic, and robot-assisted radical prostatectomy for localized prostate cancer. World J Urol. Oct 2009;27(5):599-605. doi:10.1007/s00345-009-0379-z

182. Dubbelman Y, Groen J, Wildhagen M, Rikken B, Bosch R. The recovery of urinary continence after radical retropubic prostatectomy: a randomized trial comparing the effect of physiotherapist-guided pelvic floor muscle exercises with guidance by an instruction folder only. BJU Int. Aug 2010;106(4):515-22. doi:10.1111/j.1464-410X.2010.09159.x

183. Durand X, Vaessen C, Bitker MO, Richard F. [Retropubic, laparoscopic and robot-assisted total prostatectomies: comparison of postoperative course and histological and functional results based on a series of 86 prostatectomies]. Prog Urol. Jan 2008;18(1):60-7. doi:10.1016/j.purol.2007.10.013

184. Duthie JB, Pickford JE, Gilling PJ. Robot-assisted laparoscopic prostatectomy: a 2010 update. N Z Med J. Nov 2010;123(1325):30-4.

185. Eastham JA, Kattan MW, Riedel E, et al. Variations among individual surgeons in the rate of positive surgical margins in radical prostatectomy specimens. J Urol. Dec 2003;170(6 Pt 1):2292-5. doi:10.1097/01.ju.0000091100.83725.51

186. Eastham JA, Kuroiwa K, Ohori M, et al. Prognostic significance of location of positive margins in radical prostatectomy specimens. Urology. Nov 2007;70(5):965-9. doi:10.1016/j.urology.2007.08.040

187. Eastham JA, Scardino PT, Kattan MW. Predicting an optimal outcome after radical prostatectomy: the trifecta nomogram. J Urol. Jun 2008;179(6):2207-10; discussion 2210-1. doi:10.1016/j.juro.2008.01.106

188. Eden CG, Arora A, Rouse P. Extended vs standard pelvic lymphadenectomy during laparoscopic radical prostatectomy for intermediate- and high-risk prostate cancer. BJU Int. Aug 2010;106(4):537-42. doi:10.1111/j.1464-410X.2009.09161.x

189. Eden CG, Cahill D, Vass JA, Adams TH, Dauleh MI. Laparoscopic radical prostatectomy: the initial UK series. BJU Int. Dec 2002;90(9):876-82.

190. Eden CG, Chang CM, Gianduzzo T, Moon DA. The impact of obesity on laparoscopic radical prostatectomy. BJU Int. Dec 2006;98(6):1279-82. doi:10.1111/j.1464-410X.2006.06443.x

191. Eden CG, King D, Kooiman GG, Adams TH, Sullivan ME, Vass JA. Transperitoneal or extraperitoneal laparoscopic radical prostatectomy: does the approach matter? J Urol. Dec 2004;172(6 Pt 1):2218-23.

192. Eden CG, Neill MG, Louie-Johnsun MW. The first 1000 cases of laparoscopic radical prostatectomy in the UK: evidence of multiple 'learning curves'. BJU Int. May 2009;103(9):1224-30. doi:10.1111/j.1464-410X.2008.08169.x

193. Eden CG, Richards AJ, Ooi J, Moon DA, Laczko I. Previous bladder outlet surgery does not affect medium-term outcomes after laparoscopic radical prostatectomy. BJU Int. Feb 2007;99(2):399-402. doi:10.1111/j.1464-410X.2006.06642.x

194. Eden CG, Zacharakis E, Bott S. The learning curve for laparoscopic extended pelvic lymphadenectomy for intermediate- and high-risk prostate cancer: implications for compliance with existing guidelines. BJU Int. Aug 2013;112(3):346-54. doi:10.1111/j.1464-410X.2012.11671.x

195. Eden CG, Moschonas D, Soares R. Urinary continence four weeks following Retzius-sparing robotic radical prostatectomy: The UK experience. Journal of Clinical Urology. 2018;11(1):15-20. doi:10.1177/2051415817706635

196. Egawa S, Kuruma H, Suyama K, Iwamura M, Baba S. Delayed recovery of urinary continence after laparoscopic radical prostatectomy. Int J Urol. Apr 2003;10(4):207-12.

197. Eggener SE, Yossepowitch O, Serio AM, Vickers AJ, Scardino PT, Eastham JA. Radical prostatectomy shortly after prostate biopsy does not affect operative difficulty or efficacy. Urology. Jun 2007;69(6):1128-33. doi:10.1016/j.urology.2007.01.089

198. Eichelberg C, Erbersdobler A, Haese A, et al. Frozen section for the management of intraoperatively detected palpable tumor lesions during nerve-sparing scheduled radical prostatectomy. Eur Urol. Jun 2006;49(6):1011-6; discussion 1016-8. doi:10.1016/j.eururo.2006.02.035

199. El-Feel A, Davis JW, Deger S, et al. Positive margins after laparoscopic radical prostatectomy: a prospective study of 100 cases performed by 4 different surgeons. Eur Urol. Jun 2003;43(6):622-6.

200. Ellison LM, Heaney JA, Birkmeyer JD. The effect of hospital volume on mortality and resource use after radical prostatectomy. J Urol. Mar 2000;163(3):867-9.

201. Ellison LM, Trock BJ, Poe NR, Partin AW. The effect of hospital volume on cancer control after radical prostatectomy. J Urol. Jun 2005;173(6):2094-8. doi:10.1097/01.ju.0000158156.80315.fe

202. Emiliozzi P, Martini M, d'Elia G, Scarpone P, Pansadoro A, Pansadoro V. A new technique for laparoscopic vesicourethral anastomosis: preliminary report. Urology. Dec 2008;72(6):1341-3. doi:10.1016/j.urology.2008.07.003

203. Engel JD, Kao WW, Williams SB, Hong YM. Oncologic outcome of robot-assisted laparoscopic prostatectomy in the high-risk setting. J Endourol. Dec 2010;24(12):1963-6. doi:10.1089/end.2010.0305

204. Erdogru T, Teber D, Frede T, Marrero R, Hammady A, Rassweiler J. The effect of previous transperitoneal laparoscopic inguinal herniorrhaphy on transperitoneal laparoscopic radical prostatectomy. J Urol. Mar 2005;173(3):769-72. doi:10.1097/01.ju.0000152649.49630.06

205. Erdogru T, Teber D, Frede T, et al. Comparison of transperitoneal and extraperitoneal laparoscopic radical prostatectomy using match-pair analysis. Eur Urol. Sep 2004;46(3):312-9; discussion 320. doi:10.1016/j.eururo.2004.05.004

206. Erdogru T, Yucel S, Frede T, Baykara M, Rassweiler J, Teber D. Laparoscopic radical prostatectomy: transfer validity. Int J Urol. May 2010;17(5):476-82. doi:10.1111/j.1442-2042.2010.02515.x

207. Escudero J, Backhaus M, Redon A, et al. Use of a barbed suture for the urethro-vesical anastomosis during the learning curve of the endoscopic extraperitoneal radical prostatectomy. Arch Esp Urol. 2012;65:752-8.

208. Evans SM, Millar JL, Frydenberg M, et al. Positive surgical margins: rate, contributing factors and impact on further treatment: findings from the Prostate Cancer Registry. BJU Int. Nov 2014;114(5):680-90. doi:10.1111/bju.12509

209. Farnham SB, Webster TM, Herrell SD, Smith JA. Intraoperative blood loss and transfusion requirements for robotic-assisted radical prostatectomy versus radical retropubic prostatectomy. Urology. Feb 2006;67(2):360-3. doi:10.1016/j.urology.2005.08.029

210. Feicke A, Baumgartner M, Talimi S, et al. Robotic-assisted laparoscopic extended pelvic lymph node dissection for prostate cancer: surgical technique and experience with the first 99 cases. Eur Urol. Apr 2009;55(4):876-83. doi:10.1016/j.eururo.2008.12.006

211. Ficarra V, Novara G, Fracalanza S, et al. A prospective, non-randomized trial comparing robot-assisted laparoscopic and retropubic radical prostatectomy in one European institution. BJU Int. Aug 2009;104(4):534-9. doi:10.1111/j.1464-410X.2009.08419.x

212. Ficarra V, Novara G, Galfano A, et al. Twelve-month self-reported quality of life after retropubic radical prostatectomy: a prospective study with Rand 36-Item Health Survey (Short Form-36). BJU Int. Feb 2006;97(2):274-8. doi:10.1111/j.1464-410X.2005.05893.x

213. Ficarra V, Novara G, Secco S, et al. Predictors of positive surgical margins after laparoscopic robot assisted radical prostatectomy. J Urol. Dec 2009;182(6):2682-8. doi:10.1016/j.juro.2009.08.037

214. Finley DS, Chang A, Morales B, Osann K, Skarecky D, Ahlering T. Impact of regional hypothermia on urinary continence and potency after robot-assisted radical prostatectomy. J Endourol. Jul 2010;24(7):1111-6. doi:10.1089/end.2010.0122.

215. Finley DS, Osann K, Chang A, Santos R, Skarecky D, Ahlering TE. Hypothermic robotic radical prostatectomy: impact on continence. J Endourol. Sep 2009;23(9):1443-50. doi:10.1089/end.2009.0411

216. Finley DS, Rodriguez E, Jr., Ahlering TE. Combined inguinal hernia repair with prosthetic mesh during transperitoneal robot assisted laparoscopic radical prostatectomy: a 4-year experience. J Urol. Oct 2007;178(4 Pt 1):1296-9; discussion 1299-300. doi:10.1016/j.juro.2007.05.154

217. Finley DS, Rodriguez E, Skarecky DW, Ahlering TE. Quantitative and qualitative analysis of the recovery of potency after radical prostatectomy: effect of unilateral vs bilateral nerve sparing. BJU Int. Nov 2009;104(10):1484-9. doi:10.1111/j.1464-410X.2009.08546.x

218. Finley DS, Savatta D, Rodriguez E, Kopelan A, Ahlering TE. Transperitoneal robotic-assisted laparoscopic radical prostatectomy and inguinal herniorrhaphy. J Robot Surg. 2008;1(4):269-72. doi:10.1007/s11701-007-0051-9

219. Fischer B, Engel N, Fehr JL, John H. Complications of robotic assisted radical prostatectomy. World J Urol. Dec 2008;26(6):595-602. doi:10.1007/s00345-008-0287-7

220. Flury SC, Starnes DN, Steers WD. Application of fibrin sealant at the urethrovesical anastomosis in robotic assisted radical prostatectomy: does it enable earlier Foley catheter and Jackson-Pratt drain removal? J Robot Surg. 2008;1(4):303-6. doi:10.1007/s11701-007-0061-7

221. Foley CL, Bott SR, Thomas K, Parkinson MC, Kirby RS. A large prostate at radical retropubic prostatectomy does not adversely affect cancer control, continence or potency rates. BJU Int. Sep 2003;92(4):370-4. doi:10.1046/j.1464-410x.2003.04361.x

222. Forsmark A, Gehrman J, Angenete E, et al. Health Economic Analysis of Open and Robot-assisted Laparoscopic Surgery for Prostate Cancer Within the Prospective Multicentre LAPPRO Trial. Eur Urol. 12 2018;74(6):816-824. doi:10.1016/j.eururo.2018.07.038

223. Forster JA, Palit V, Myatt A, Hadi S, Bryan NP. Technical description and outcomes of a continuous anastomosis in open radical prostatectomy. BJU Int. Oct 2009;104(7):929-33. doi:10.1111/j.1464-410X.2009.08526.x

224. Fracalanza S, Ficarra V, Cavalleri S, et al. Is robotically assisted laparoscopic radical prostatectomy less invasive than retropubic radical prostatectomy? Results from a prospective, unrandomized, comparative study. BJU Int. May 2008;101(9):1145-9. doi:10.1111/j.1464-410X.2008.07513.x

225. Frede T, Erdogru T, Zukosky D, Gulkesen H, Teber D, Rassweiler J. Comparison of training modalities for performing laparoscopic radical prostatectomy: experience with 1,000 patients. J Urol. Aug 2005;174(2):673-8; discussion 678. doi:10.1097/01.ju.0000165152.61295.cb

226. Freire MP, Weinberg AC, Lei Y, et al. Anatomic bladder neck preservation during robotic-assisted laparoscopic radical prostatectomy: description of technique and outcomes. Eur Urol. Dec 2009;56(6):972-80. doi:10.1016/j.eururo.2009.09.017

227. Friedlander DF, Alemozaffar M, Hevelone ND, Lipsitz SR, Hu JC. Stepwise description and outcomes of bladder neck sparing during robot-assisted laparoscopic radical prostatectomy. J Urol. Nov 2012;188(5):1754-60. doi:10.1016/j.juro.2012.07.045

228. Friðriksson J, Holmberg E, Adolfsson J, et al. Rehospitalization after radical prostatectomy in a nationwide, population based study. J Urol. Jul 2014;192(1):112-9. doi:10.1016/j.juro.2014.01.109

229. Froehner M, Koch R, Leike S, Novotny V, Twelker L, Wirth MP. Urinary tract-related quality of life after radical prostatectomy: open retropubic versus robot-assisted laparoscopic approach. Urol Int. 2013;90(1):36-40. doi:10.1159/000345320

230. Froehner M, Novotny V, Koch R, Leike S, Twelker L, Wirth MP. Perioperative complications after radical prostatectomy: open versus robot-assisted laparoscopic approach. Urol Int. 2013;90(3):312-5. doi:10.1159/000345323

231. Fromont G, Guillonneau B, Validire P, Vallancien G. Laparoscopic radical prostatectomy. preliminary pathologic evaluation. Urology. Oct 2002;60(4):661-5.

232. Frota R, Stein RJ, Turna B, et al. Are prostate needle biopsies predictive of the laterality of significant cancer and positive surgical margins? BJU Int. Dec 2009;104(11):1599-603. doi:10.1111/j.1464-410X.2009.08648.x

233. Frota R, Turna B, Santos BM, Lin YC, Gill IS, Aron M. The effect of prostate weight on the outcomes of laparoscopic radical prostatectomy. BJU Int. Mar 2008;101(5):589-93. doi:10.1111/j.1464-410X.2007.07263.x

234. Fukuhara H, Inoue K, Satake H, et al. Photodynamic diagnosis of positive margin during radical prostatectomy: preliminary experience with 5-aminolevulinic acid. Int J Urol. Aug 2011;18(8):585-91. doi:10.1111/j.1442-2042.2011.02789.x

235. Gainsburg DM, Wax D, Reich DL, Carlucci JR, Samadi DB. Intraoperative management of robotic-assisted versus open radical prostatectomy. JSLS. 2010 Jan-Mar 2010;14(1):1-5. doi:10.4293/108680810X12674612014266

236. Galfano A, Ascione A, Grimaldi S, Petralia G, Strada E, Bocciardi AM. A new anatomic approach for robot-assisted laparoscopic prostatectomy: a feasibility study for completely intrafascial surgery. Eur Urol. Sep 2010;58(3):457-61. doi:10.1016/j.eururo.2010.06.008

237. Galli S, Simonato A, Bozzola A, et al. Oncologic outcome and continence recovery after laparoscopic radical prostatectomy: 3 years' follow-up in a "second generation center". Eur Urol. May 2006;49(5):859-65. doi:10.1016/j.eururo.2006.01.035

238. Gallo L, Perdonà S, Autorino R, et al. Vesicourethral anastomosis during radical retropubic prostatectomy: does the number of sutures matter? Urology. Mar 2007;69(3):547-51. doi:10.1016/j.urology.2006.12.016

239. Ganzer R, Blana A, Denzinger S, et al. Intraoperative photodynamic evaluation of surgical margins during endoscopic extraperitoneal radical prostatectomy with the use of 5-aminolevulinic acid. J Endourol. Sep 2009;23(9):1387-94. doi:10.1089/end.2009.0374

240. Gao X, Wang KB, Pu XY, Zhou XF, Qiu JG. Modified apical dissection of the prostate improves early continence in laparoscopic radical prostatectomy: technique and initial results. J Cancer Res Clin Oncol. Apr 2010;136(4):511-6. doi:10.1007/s00432-009-0683-4

241. Gao X, Zhou JH, Li LY, Qiu JG, Pu XY. Laparoscopic radical prostatectomy: oncological and functional results of 126 patients with a minimum 3-year follow-up at a single Chinese institute. Asian J Androl. Sep 2009;11(5):548-56. doi:10.1038/aja.2009.42

242. Gao Z, Wu J, Wang K, et al. Comparison of, the extraperitoneal and transperitoneal laparoscopic radical prostatectomy. Article. Chinese Medical Journal. DEC 20 2006 2006;119(24):2125-2128.

243. Geraerts I, Van Poppel H, Devoogdt N, Van Cleynenbreugel B, Joniau S, Van Kampen M. Prospective evaluation of urinary incontinence, voiding symptoms and quality of life after open and robot-assisted radical prostatectomy. BJU Int. Nov 2013;112(7):936-43. doi:10.1111/bju.12258

244. Gettman MT, Hoznek A, Salomon L, et al. Laparoscopic radical prostatectomy: description of the extraperitoneal approach using the da Vinci robotic system. J Urol. Aug 2003;170(2 Pt 1):416-9. doi:10.1097/01.ju.0000076015.88739.a2

245. Ghavamian R, Knoll A, Boczko J, Melman A. Comparison of operative and functional outcomes of laparoscopic radical prostatectomy and radical retropubic prostatectomy: single surgeon experience. Urology. Jun 2006;67(6):1241-6. doi:10.1016/j.urology.2005.12.017

246. Ghavamian R, Schenk G, Hoenig DM, Williot P, Melman A. Overcoming the steep learning curve of laparoscopic radical prostatectomy: single-surgeon experience. J Endourol. Aug 2004;18(6):567-71. doi:10.1089/end.2004.18.567

247. Giberti C, Chiono L, Gallo F, Schenone M, Gastaldi E. Radical retropubic prostatectomy versus brachytherapy for low-risk prostatic cancer: a prospective study. World J Urol. Oct 2009;27(5):607-12. doi:10.1007/s00345-009-0418-9

248. Gill IS, Ukimura O. Thermal energy-free laparoscopic nerve-sparing radical prostatectomy: one-year potency outcomes. Urology. Aug 2007;70(2):309-14. doi:10.1016/j.urology.2007.03.072

249. Gillitzer R, Thomas C, Wiesner C, et al. Single center comparison of anastomotic strictures after radical perineal and radical retropubic prostatectomy. Urology. Aug 2010;76(2):417-22. doi:10.1016/j.urology.2009.10.009

250. Ginzburg S, Hu F, Staff I, et al. Does prior abdominal surgery influence outcomes or complications of robotic-assisted laparoscopic radical prostatectomy? Urology. Nov 2010;76(5):1125-9. doi:10.1016/j.urology.2010.03.039

251. Glickman L, Godoy G, Lepor H. Changes in continence and erectile function between 2 and 4 years after radical prostatectomy. J Urol. Feb 2009;181(2):731-5. doi:10.1016/j.juro.2008.10.019

252. Goeman L, Salomon L, La De Taille A, et al. Long-term functional and oncological results after retroperitoneal laparoscopic prostatectomy according to a prospective evaluation of 550 patients. World J Urol. Aug 2006;24(3):281-8. doi:10.1007/s00345-006-0054-6

253. Golabek T, Jaskulski J, Jarecki P, Dudek P, Szopiński T, Chłosta P. Laparoscopic radical prostatectomy with bladder neck preservation: positive surgical margin and urinary continence status. Wideochir Inne Tech Maloinwazyjne. Sep 2014;9(3):362-70. doi:10.5114/wiitm.2014.45085

254. Golabek T, Wiatr T, Przydacz M, et al. Optimizing the formation of vesicourethral anastomosis and reduction of procedure time. A two-year experience with a modified technique for endoscopic running vesicourethral anastomosis. Cent European J Urol. 2015;68(3):296-301. doi:10.5173/ceju.2015.617

255. Gomez CA, Soloway MS, Civantos F, Hachiya T. Bladder neck preservation and its impact on positive surgical margins during radical prostatectomy. Urology. Dec 1993;42(6):689-93; discussion 693-4. doi:10.1016/0090-4295(93)90534-h

256. Gondo T, Yoshioka K, Hashimoto T, et al. The powerful impact of double-layered posterior rhabdosphincter reconstruction on early recovery of urinary continence after robot-assisted radical prostatectomy. J Endourol. Sep 2012;26(9):1159-64. doi:10.1089/end.2012.0067

257. Gontero P, Marchioro G, Pisani R, et al. Is radical prostatectomy feasible in all cases of locally advanced non-bone metastatic prostate cancer? Results of a single-institution study. Eur Urol. Apr 2007;51(4):922-9; discussion 929-30. doi:10.1016/j.eururo.2006.08.050

258. Gonzalgo ML, Pavlovich CP, Trock BJ, Link RE, Sullivan W, Su LM. Classification and trends of perioperative morbidities following laparoscopic radical prostatectomy. J Urol. Jul 2005;174(1):135-9; discussion 139. doi:10.1097/01.ju.0000161607.04334.26

259. Good DW, Stewart GD, Stolzenburg JU, McNeill SA. Analysis of the pentafecta learning curve for laparoscopic radical prostatectomy. World J Urol. Oct 2014;32(5):1225-33. doi:10.1007/s00345-013-1198-9

260. Gosseine PN, Mangin P, Leclers F, Cormier L. [Pure laparoscopic versus robotic-assisted laparoscopic radical prostatectomy: comparative study to assess functional urinary outcomes]. Prog Urol. Oct 2009;19(9):611-7. doi:10.1016/j.purol.2009.05.008

261. Gözen AS, Akin Y, Ates M, Hruza M, Rassweiler J. Impact of laparoscopic radical prostatectomy on clinical T3 prostate cancer: experience of a single centre with long-term follow-up. BJU Int. Jul 2015;116(1):102-8. doi:10.1111/bju.12710

262. Gözen AS, Akin Y, Özden E, Ates M, Hruza M, Rassweiler J. Impact of body mass index on outcomes of laparoscopic radical prostatectomy with long-term follow-up. Scand J Urol. Feb 2015;49(1):70-6. doi:10.3109/21681805.2014.920416

263. Gözen AS, Tokas T, Akin Y, Klein J, Rassweiler J. Impact of barbed suture in controlling the dorsal vein complex during laparoscopic radical prostatectomy. Minim Invasive Ther Allied Technol. Apr 2015;24(2):108-13. doi:10.3109/13645706.2014.960940

264. Graefen M, Michl UHG, Heinzer H, et al. Indication, Technique and Outcome of Retropubic Nerve-Sparing Radical Prostatectomy. EAU Update Series. 2005;3(2):77-85. doi:doi.org/10.1016/j.euus.2005.03.008

265. Gralla O, Haas F, Knoll N, et al. Fast-track surgery in laparoscopic radical prostatectomy: basic principles. World J Urol. Apr 2007;25(2):185-91. doi:10.1007/s00345-006-0139-2

266. Greco F, Hoda MR, Wagner S, et al. Bilateral vs unilateral laparoscopic intrafascial nerve-sparing radical prostatectomy: evaluation of surgical and functional outcomes in 457 patients. BJU Int. Aug 2011;108(4):583-7. doi:10.1111/j.1464-410X.2010.09836.x

267. Greco F, Wagner S, Hoda MR, et al. Laparoscopic vs open retropubic intrafascial nerve-sparing radical prostatectomy: surgical and functional outcomes in 300 patients. BJU Int. Aug 2010;106(4):543-7. doi:10.1111/j.1464-410X.2009.09157.x

268. Greco KA, Meeks JJ, Wu S, Nadler RB. Robot-assisted radical prostatectomy in men aged > or =70 years. BJU Int. Nov 2009;104(10):1492-5. doi:10.1111/j.1464-410X.2009.08718.x

269. Gregori A, Simonato A, Lissiani A, Bozzola A, Galli S, Gaboardi F. Laparoscopic radical prostatectomy: perioperative complications in an initial and consecutive series of 80 cases. Eur Urol. Aug 2003;44(2):190-4; discussion 194.

270. Gregorio SA, Rivas JG, Molina SS, et al. Laparoscopic radical prostatectomy training for residents: Hospital Universitario La Paz model. Cent European J Urol. 2014;67(3):247-52. doi:10.5173/ceju.2014.03.art7

271. Grossfeld GD, Chang JJ, Broering JM, et al. Impact of positive surgical margins on prostate cancer recurrence and the use of secondary cancer treatment: data from the CaPSURE database. J Urol. Apr 2000;163(4):1171-7; quiz 1295.

272. Grossi FS, Di Lena S, Barnaba D, et al. Laparoscopic versus open radical retropubic prostatectomy: a case-control study at a single institution. Arch Ital Urol Androl. Jun 2010;82(2):109-12.

273. Guazzoni G, Cestari A, Naspro R, et al. Intra- and peri-operative outcomes comparing radical retropubic and laparoscopic radical prostatectomy: results from a prospective, randomised, single-surgeon study. Eur Urol. Jul 2006;50(1):98-104. doi:10.1016/j.eururo.2006.02.051

274. Guillonneau B, Cathelineau X, Doublet JD, Baumert H, Vallancien G. Laparoscopic radical prostatectomy: assessment after 550 procedures. Crit Rev Oncol Hematol. Aug 2002;43(2):123-33.

275. Guillonneau B, el-Fettouh H, Baumert H, et al. Laparoscopic radical prostatectomy: oncological evaluation after 1,000 cases a Montsouris Institute. J Urol. Apr 2003;169(4):1261-6. doi:10.1097/01.ju.0000055141.36916.be

276. Guillonneau B, Rozet F, Cathelineau X, et al. Perioperative complications of laparoscopic radical prostatectomy: the Montsouris 3-year experience. J Urol. Jan 2002;167(1):51-6.

277. Guillonneau B, Vallancien G. Laparoscopic radical prostatectomy: the Montsouris experience. J Urol. Feb 2000;163(2):418-22.

278. Gumus E, Boylu U, Turan T, Onol FF. The learning curve of robot-assisted radical prostatectomy. J Endourol. Oct 2011;25(10):1633-7. doi:10.1089/end.2011.0071

279. Gupta NP, Singh P, Nayyar R. Outcomes of robot-assisted radical prostatectomy in men with previous transurethral resection of prostate. BJU Int. Nov 2011;108(9):1501-5. doi:10.1111/j.1464-410X.2011.10113.x

280. Guru KA, Perlmutter AE, Sheldon MJ, et al. Apical margins after robot-assisted radical prostatectomy: does technique matter? J Endourol. Jan 2009;23(1):123-7. doi:10.1089/end.2008.0398

281. Habib AS, Polascik TJ, Weizer AZ, et al. Lidocaine patch for postoperative analgesia after radical retropubic prostatectomy. Anesth Analg. Jun 2009;108(6):1950-3. doi:10.1213/ane.0b013e3181a21185

282. Haglind E, Carlsson S, Stranne J, et al. Urinary Incontinence and Erectile Dysfunction After Robotic Versus Open Radical Prostatectomy: A Prospective, Controlled, Nonrandomised Trial. Eur Urol. 08 2015;68(2):216-25. doi:10.1016/j.eururo.2015.02.029

283. Hakimi AA, Blitstein J, Feder M, Shapiro E, Ghavamian R. Direct comparison of surgical and functional outcomes of robotic-assisted versus pure laparoscopic radical prostatectomy: single-surgeon experience. Urology. Jan 2009;73(1):119-23. doi:10.1016/j.urology.2008.08.491

284. Ham WS, Park SY, Kim WT, Koo KC, Lee YS, Choi YD. Open versus robotic radical prostatectomy: a prospective analysis based on a single surgeon's experience. J Robot Surg. Dec 2008;2(4):235-41. doi:10.1007/s11701-008-0111-9

285. Ham WS, Park SY, Rha KH, Kim WT, Choi YD. Robotic radical prostatectomy for patients with locally advanced prostate cancer is feasible: results of a single-institution study. J Laparoendosc Adv Surg Tech A. Jun 2009;19(3):329-32. doi:10.1089/lap.2008.0344

286. Hampton L, Nelson RA, Satterthwaite R, Wilson T, Crocitto L. Patients with prior TURP undergoing robot-assisted laparoscopic radical prostatectomy have higher positive surgical margin rates. J Robot Surg. Dec 2008;2(4):213-6. doi:10.1007/s11701-008-0121-7

287. Han M, Partin AW, Chan DY, Walsh PC. An evaluation of the decreasing incidence of positive surgical margins in a large retropubic prostatectomy series. J Urol. Jan 2004;171(1):23-6. doi:10.1097/01.ju.0000098604.09395.27

288. Han M, Partin AW, Pound CR, Epstein JI, Walsh PC. Long-term biochemical disease-free and cancer-specific survival following anatomic radical retropubic prostatectomy. The 15-year Johns Hopkins experience. Urol Clin North Am. Aug 2001;28(3):555-65. doi:10.1016/s0094-0143(05)70163-4

289. Hanchanale VS, Javlé P. Impact of hospital provider volume on outcome for radical urological cancer surgery in England. Urol Int. 2010;85(1):11-5. doi:10.1159/000318631

290. Hara I, Kawabata G, Miyake H, et al. Comparison of quality of life following laparoscopic and open prostatectomy for prostate cancer. J Urol. Jun 2003;169(6):2045-8. doi:10.1097/01.ju.0000063961.99940.6c

291. Hara I, Kawabata G, Tanaka K, et al. Oncological outcome of laparoscopic prostatectomy. Int J Urol. Jun 2007;14(6):515-20. doi:10.1111/j.1442-2042.2007.01773.x

292. Harke NN, Godes M, Wagner C, et al. Fluorescence-supported lymphography and extended pelvic lymph node dissection in robot-assisted radical prostatectomy: a prospective, randomized trial. World J Urol. Nov 2018;36(11):1817-1823. doi:10.1007/s00345-018-2330-7

293. Harke N, Godes M, Habibzada J, et al. Postoperative patient comfort in suprapubic drainage versus transurethral catheterization following robot-assisted radical prostatectomy: a prospective randomized clinical trial. World J Urol. Mar 2017;35(3):389-394. doi:10.1007/s00345-016-1883-6

294. Harty NJ, Kozinn SI, Canes D, Sorcini A, Moinzadeh A. Comparison of positive surgical margin rates in high risk prostate cancer: open versus minimally invasive radical prostatectomy. Int Braz J Urol. 2013 Sep-Oct 2013;39(5):639-46; discussion 647-8.

295. Hashimoto T, Yoshioka K, Horiguchi Y, et al. Clinical effect of a positive surgical margin without extraprostatic extension after robot-assisted radical prostatectomy. Urol Oncol. Dec 2015;33(12):503.e1-6. doi:10.1016/j.urolonc.2015.07.009

296. Haskins AE, Han PK, Lucas FL, Bristol I, Hansen M. Development of clinical models for predicting erectile function after localized prostate cancer treatment. Int J Urol. Dec 2014;21(12):1227-33. doi:10.1111/iju.12566

297. Hatiboglu G, Teber D, Tichy D, et al. Predictive factors for immediate continence after radical prostatectomy. World J Urol. Jan 2016;34(1):113-20. doi:10.1007/s00345-015-1594-4

298. Hautmann RE, Sauter TW, Wenderoth UK. Radical retropubic prostatectomy: morbidity and urinary continence in 418 consecutive cases. Urology. Feb 1994;43(2 Suppl):47-51. doi:10.1016/0090-4295(94)90218-6

299. Heathcote PS, Mactaggart PN, Boston RJ, James AN, Thompson LC, Nicol DL. Health-related quality of life in Australian men remaining disease-free after radical prostatectomy. Med J Aust. May 1998;168(10):483-6.

300. Heinrich E, Schön G, Schiefelbein F, Michel MS, Trojan L. Clinical impact of intraoperative frozen sections during nerve-sparing radical prostatectomy. World J Urol. Dec 2010;28(6):709-13. doi:10.1007/s00345-010-0529-3

301. Heinzer H, Graefen M, Noldus J, Hammerer P, Huland H. Early complication of anatomical radical retropubic prostatectomy: lessons from a single-center experience. Urol Int. 1997;59(1):30-3. doi:10.1159/000283013

302. Heldt JP, Jellison FC, Yuen WD, et al. Patients with end-stage renal disease are candidates for robot-assisted laparoscopic radical prostatectomy. J Endourol. Jul 2011;25(7):1175-80. doi:10.1089/end.2010.0680

303. Hellawell GO, Moon DA. Laparoscopic radical prostatectomy: reducing the learning curve. Urology. Dec 2008;72(6):1347-50. doi:10.1016/j.urology.2007.12.027

304. Hemal AK, Agarwal MM, Babbar P. Impact of newer unidirectional and bidirectional barbed suture on vesicourethral anastomosis during robot-assisted radical prostatectomy and its comparison with polyglecaprone-25 suture: an initial experience. Int Urol Nephrol. Feb 2012;44(1):125-32. doi:10.1007/s11255-011-9967-0

305. Herrmann TR, Rabenalt R, Stolzenburg JU, et al. Oncological and functional results of open, robot-assisted and laparoscopic radical prostatectomy: does surgical approach and surgical experience matter? World J Urol. Apr 2007;25(2):149-60. doi:10.1007/s00345-007-0164-9

306. Hisasue S, Takahashi A, Kato R, et al. Early and late complications of radical retropubic prostatectomy: experience in a single institution. Jpn J Clin Oncol. May 2004;34(5):274-9. doi:10.1093/jjco/hyh042

307. Hocaoglu Y, Bastian P, Buchner A, et al. Impact of previous mesh hernia repair on the performance of open radical prostatectomy - complications and functional outcome. BJU Int. Dec 2010;106(11):1628-31. doi:10.1111/j.1464-410X.2010.09495.x

308. Hoda MR, Hamza A, Greco F, et al. Management of localized prostate cancer by retropubic radical prostatectomy in patients after renal transplantation. Nephrol Dial Transplant. Oct 2010;25(10):3416-20. doi:10.1093/ndt/gfq193

309. Hohwü L, Akre O, Pedersen KV, Jonsson M, Nielsen CV, Gustafsson O. Open retropubic prostatectomy versus robot-assisted laparoscopic prostatectomy: a comparison of length of sick leave. Scand J Urol Nephrol. 2009;43(4):259-64. doi:10.1080/00365590902834802

310. Hong H, Mel L, Taylor J, Wu Q, Reeves H. Effects of robotic-assisted laparoscopic prostatectomy on surgical pathology specimens. Diagn Pathol. Mar 2012;7:24. doi:10.1186/1746-1596-7-24

311. Hong JY, Kim JY, Choi YD, Rha KH, Yoon SJ, Kil HK. Incidence of venous gas embolism during robotic-assisted laparoscopic radical prostatectomy is lower than that during radical retropubic prostatectomy. Br J Anaesth. Dec 2010;105(6):777-81. doi:10.1093/bja/aeq247

312. Hong JY, Kim WO, Kil HK. Detection of subclinical CO2 embolism by transesophageal echocardiography during laparoscopic radical prostatectomy. Urology. Mar 2010;75(3):581-4. doi:10.1016/j.urology.2009.04.064

313. Hong SK, Kim DS, Lee WK, et al. Significance of postbiopsy hemorrhage observed on preoperative magnetic resonance imaging in performing robot-assisted laparoscopic radical prostatectomy. World J Urol. Dec 2010;28(6):721-6. doi:10.1007/s00345-010-0506-x

314. Hong SK, Lee ST, Kim SS, et al. Effect of bony pelvic dimensions measured by preoperative magnetic resonance imaging on performing robot-assisted laparoscopic prostatectomy. BJU Int. Sep 2009;104(5):664-8. doi:10.1111/j.1464-410X.2009.08624.x

315. Hong YM, Sutherland DE, Linder B, Engel JD. "Learning curve" may not be enough: assessing the oncological experience curve for robotic radical prostatectomy. J Endourol. Mar 2010;24(3):473-7. doi:10.1089/end.2009.0121

316. Horstmann M, Vollmer C, Schwab C, et al. Single-centre evaluation of the extraperitoneal and transperitoneal approach in robotic-assisted radical prostatectomy. Scand J Urol Nephrol. Apr 2012;46(2):117-23. doi:10.3109/00365599.2011.637957

317. Hoznek A, Antiphon P, Borkowski T, et al. Assessment of surgical technique and perioperative morbidity associated with extraperitoneal versus transperitoneal laparoscopic radical prostatectomy. Urology. Mar 2003;61(3):617-22.

318. Hoznek A, Salomon L, Olsson LE, et al. Laparoscopic radical prostatectomy. The Créteil experience. Eur Urol. Jul 2001;40(1):38-45. doi:49747

319. Hruza M, Bermejo JL, Flinspach B, et al. Long-term oncological outcomes after laparoscopic radical prostatectomy. BJU Int. Feb 2013;111(2):271-80. doi:10.1111/j.1464-410X.2012.11317.x

320. Hruza M, Weiss HO, Pini G, et al. Complications in 2200 consecutive laparoscopic radical prostatectomies: standardised evaluation and analysis of learning curves. Eur Urol. Nov 2010;58(5):733-41. doi:10.1016/j.eururo.2010.08.024

321. Hsu EI, Hong EK, Lepor H. Influence of body weight and prostate volume on intraoperative, perioperative, and postoperative outcomes after radical retropubic prostatectomy. Urology. Mar 2003;61(3):601-6. doi:10.1016/s0090-4295(02)02422-6

322. Hu JC, Elkin EP, Pasta DJ, et al. Predicting quality of life after radical prostatectomy: results from CaPSURE. J Urol. Feb 2004;171(2 Pt 1):703-7; discussion 707-8. doi:10.1097/01.ju.0000107964.61300.f6

323. Hu JC, Gold KF, Pashos CL, Mehta SS, Litwin MS. Role of surgeon volume in radical prostatectomy outcomes. J Clin Oncol. Feb 2003;21(3):401-5. doi:10.1200/JCO.2003.05.169

324. Hu JC, Gu X, Lipsitz SR, et al. Comparative effectiveness of minimally invasive vs open radical prostatectomy. JAMA. Oct 2009;302(14):1557-64. doi:10.1001/jama.2009.1451

325. Hu JC, Nelson RA, Wilson TG, et al. Perioperative complications of laparoscopic and robotic assisted laparoscopic radical prostatectomy. J Urol. Feb 2006;175(2):541-6; discussion 546. doi:10.1016/S0022-5347(05)00156-4

326. Hu JC, Wang Q, Pashos CL, Lipsitz SR, Keating NL. Utilization and outcomes of minimally invasive radical prostatectomy. J Clin Oncol. May 2008;26(14):2278-84. doi:10.1200/JCO.2007.13.4528

327. Huang AC, Kowalczyk KJ, Hevelone ND, et al. The impact of prostate size, median lobe, and prior benign prostatic hyperplasia intervention on robot-assisted laparoscopic prostatectomy: technique and outcomes. Eur Urol. 04 2011;59(4):595-603. doi:10.1016/j.eururo.2011.01.033

328. Hull GW, Rabbani F, Abbas F, Wheeler TM, Kattan MW, Scardino PT. Cancer control with radical prostatectomy alone in 1,000 consecutive patients. J Urol. Feb 2002;167(2 Pt 1):528-34. doi:10.1097/00005392-200202000-00018

329. Hung CF, Yang CK, Ou YC. Robotic assisted laparoscopic radical prostatectomy following transurethral resection of the prostate: perioperative, oncologic and functional outcomes. Prostate Int. 2014;2(2):82-9. doi:10.12954/PI.14046

330. Hurtes X, Rouprêt M, Vaessen C, et al. Anterior suspension combined with posterior reconstruction during robot-assisted laparoscopic prostatectomy improves early return of urinary continence: a prospective randomized multicentre trial. BJU Int. Sep 2012;110(6):875-83. doi:10.1111/j.1464-410X.2011.10849.x

331. Hyams ES, Mullins JK, Pierorazio PM, Partin AW, Allaf ME, Matlaga BR. Impact of robotic technique and surgical volume on the cost of radical prostatectomy. J Endourol. Mar 2013;27(3):298-303. doi:10.1089/end.2012.0147

332. Ihsan-Tasci A, Simsek A, Dogukan-Torer MB, et al. Oncologic results, functional outcomes, and complication rates of transperitoneal robotic assisted radical prostatectomy: single centre's experience. Actas Urol Esp. Mar 2015;39(2):70-7. doi:10.1016/j.acuro.2014.02.021

333. Isbarn H, Jeldres C, Budäus L, et al. Effect of body mass index on histopathologic parameters: results of large European contemporary consecutive open radical prostatectomy series. Urology. Mar 2009;73(3):615-9. doi:10.1016/j.urology.2008.09.038

334. Iseki R, Ohori M, Hatano T, Tachibana M. [Urinary incontinence in early experience with robot-assisted laparoscopic prostatectomy-comparison with radical retropubic prostatectomy]. Hinyokika Kiyo. Aug 2012;58(8):409-14.

335. Ito K, Kenji S, Yoshii H, et al. Modified posterior musculofascial plate reconstruction decreases the posterior vesicourethral angle and improves urinary continence recovery in patients undergoing laparoscopic radical prostatectomy. Mol Clin Oncol. Nov 2013;1(6):970-976. doi:10.3892/mco.2013.182

336. Jackson MA, Bellas N, Siegrist T, et al. Experienced Open vs Early Robotic-assisted Laparoscopic Radical Prostatectomy: A 10-year Prospective and Retrospective Comparison. Urology. 05 2016;91:111-8. doi:10.1016/j.urology.2015.12.072

337. Jacobs BL, Montgomery JS, Dunn RL, et al. A comparison of extraperitoneal and intraperitoneal approaches for robotic prostatectomy. Surg Innov. Sep 2012;19(3):268-74. doi:10.1177/1553350611429028

338. Jacobsen NE, Moore KN, Estey E, Voaklander D. Open versus laparoscopic radical prostatectomy: a prospective comparison of postoperative urinary incontinence rates. J Urol. Feb 2007;177(2):615-9. doi:10.1016/j.juro.2006.09.022

339. Jaffe J, Castellucci S, Cathelineau X, et al. Robot-assisted laparoscopic prostatectomy: a single-institutions learning curve. Urology. Jan 2009;73(1):127-33. doi:10.1016/j.urology.2008.08.482

340. Jaffe J, Stakhovsky O, Cathelineau X, Barret E, Vallancien G, Rozet F. Surgical outcomes for men undergoing laparoscopic radical prostatectomy after transurethral resection of the prostate. J Urol. Aug 2007;178(2):483-7; discussion 487. doi:10.1016/j.juro.2007.03.114

341. Jayram G, Decastro GJ, Large MC, et al. Robotic radical prostatectomy in patients with high-risk disease: a review of short-term outcomes from a high-volume center. J Endourol. Mar 2011;25(3):455-7. doi:10.1089/end.2010.0349

342. Jenkins LC, Nogueira M, Wilding GE, et al. Median lobe in robot-assisted radical prostatectomy: evaluation and management. Urology. May 2008;71(5):810-3. doi:10.1016/j.urology.2007.12.054

343. Jeong J, Choi EY, Kim IY. Clavien classification of complications after the initial series of robot-assisted radical prostatectomy: the Cancer Institute of New Jersey/Robert Wood Johnson Medical School experience. J Endourol. Sep 2010;24(9):1457-61. doi:10.1089/end.2010.0027

344. Jeong W, Araki M, Park SY, et al. Robot-assisted laparoscopic radical prostatectomy in the Asian population: modified port configuration and ultradissection. Int J Urol. Mar 2010;17(3):297-300. doi:10.1111/j.1442-2042.2010.02480.x

345. Jo JK, Oh JJ, Lee S, et al. Can robot-assisted laparoscopic radical prostatectomy (RALP) be performed very soon after biopsy? World J Urol. Apr 2017;35(4):605-612. doi:10.1007/s00345-016-1893-4

346. Johnson EK, Hedgepeth RC, He C, Wood DP. The impact of anterior urethropexy during robotic prostatectomy on urinary and sexual outcomes. J Endourol. Apr 2011;25(4):615-9. doi:10.1089/end.2010.0413

347. Johnson I, Ottosson F, Diep LM, et al. Switching from laparoscopic radical prostatectomy to robot assisted laparoscopic prostatectomy: comparing oncological outcomes and complications. Scand J Urol. Apr 2018;52(2):116-121. doi:10.1080/21681805.2017.1420099

348. Joseph JV, Rosenbaum R, Madeb R, Erturk E, Patel HR. Robotic extraperitoneal radical prostatectomy: an alternative approach. J Urol. Mar 2006;175(3 Pt 1):945-50; discussion 951. doi:10.1016/S0022-5347(05)00340-X

349. Joseph JV, Vicente I, Madeb R, Erturk E, Patel HR. Robot-assisted vs pure laparoscopic radical prostatectomy: are there any differences? BJU Int. Jul 2005;96(1):39-42. doi:10.1111/j.1464-410X.2005.05563.x

350. Joshi AR, Spivak J, Rubach E, Goldberg G, DeNoto G. Concurrent robotic trans-abdominal pre-peritoneal (TAP) herniorrhaphy during robotic-assisted radical prostatectomy. Int J Med Robot. Sep 2010;6(3):311-4. doi:10.1002/rcs.334

351. Joshi N, de Blok W, van Muilekom E, van der Poel H. Impact of posterior musculofascial reconstruction on early continence after robot-assisted laparoscopic radical prostatectomy: results of a prospective parallel group trial. Eur Urol. Jul 2010;58(1):84-9. doi:10.1016/j.eururo.2010.03.028

352. Jung JH, Seo JW, Lim MS, et al. Extended pelvic lymph node dissection including internal iliac packet should be performed during robot-assisted laparoscopic radical prostatectomy for high-risk prostate cancer. J Laparoendosc Adv Surg Tech A. Oct 2012;22(8):785-90. doi:10.1089/lap.2011.0516

353. Jurczok A, Zacharias M, Wagner S, Hamza A, Fornara P. Prospective non-randomized evaluation of four mediators of the systemic response after extraperitoneal laparoscopic and open retropubic radical prostatectomy. BJU Int. Jun 2007;99(6):1461-6. doi:10.1111/j.1464-410X.2007.06849.x

354. Kadono Y, Ueno S, Kadomoto S, et al. Use of preoperative factors including urodynamic evaluations and nerve-sparing status for predicting urinary continence recovery after robot-assisted radical prostatectomy: Nerve-sparing technique contributes to the reduction of postprostatectomy incontinence. Neurourol Urodyn. 11 2016;35(8):1034-1039. doi:10.1002/nau.22877

355. Kalisvaart JF, Osann KE, Finley DS, Ornstein DK. Posterior reconstruction and anterior suspension with single anastomotic suture in robot-assisted laparoscopic radical prostatectomy: a simple method to improve early return of continence. J Robot Surg. Oct 2009;3(3):149-53. doi:10.1007/s11701-009-0151-9

356. Kalmar AF, Foubert L, Hendrickx JF, et al. Influence of steep Trendelenburg position and CO(2) pneumoperitoneum on cardiovascular, cerebrovascular, and respiratory homeostasis during robotic prostatectomy. Br J Anaesth. Apr 2010;104(4):433-9. doi:10.1093/bja/aeq018

357. Kang HW, Jung HD, Lee JY, et al. Prostate-specific antigen density predicts favorable pathology and biochemical recurrence in patients with intermediate-risk prostate cancer. Asian J Androl. 2016 May-Jun 2016;18(3):480-4. doi:10.4103/1008-682X.154313

358. Kao TC, Cruess DF, Garner D, et al. Multicenter patient self-reporting questionnaire on impotence, incontinence and stricture after radical prostatectomy. J Urol. Mar 2000;163(3):858-64.

359. Kaouk JH, Desai MM, Abreu SC, Papay F, Gill IS. Robotic assisted laparoscopic sural nerve grafting during radical prostatectomy: initial experience. J Urol. Sep 2003;170(3):909-12. doi:10.1097/01.ju.0000073208.18059.62

360. Karakiewicz PI, Bazinet M, Aprikian AG, Tanguay S, Elhilali MM. Thirty-day mortality rates and cumulative survival after radical retropubic prostatectomy. Urology. Dec 1998;52(6):1041-6. doi:10.1016/s0090-4295(98)00350-1

361. Karl A, Buchner A, Becker H, Staehler M, Seitz M, Stief C. Perioperative blood loss in open retropubic radical prostatectomy - Is it safe to get operated at an educational hospital? Eur J Med Res. Jul 2009;14(7):292-6. doi:10.1186/2047-783x-14-7-292

362. Kasraeian A, Barret E, Chan J, et al. Comparison of the rate, location and size of positive surgical margins after laparoscopic and robot-assisted laparoscopic radical prostatectomy. BJU Int. Oct 2011;108(7):1174-8. doi:10.1111/j.1464-410X.2010.10077.x

363. Kattan MW, Vickers AJ, Yu C, et al. Preoperative and postoperative nomograms incorporating surgeon experience for clinically localized prostate cancer. Cancer. Mar 2009;115(5):1005-10. doi:10.1002/cncr.24083

364. Katz DJ, Yee DS, Godoy G, Nogueira L, Chong KT, Coleman JA. Lymph node dissection during robotic-assisted laparoscopic prostatectomy: comparison of lymph node yield and clinical outcomes when including common iliac nodes with standard template dissection. BJU Int. Aug 2010;106(3):391-6. doi:10.1111/j.1464-410X.2009.09102.x

365. Katz R, Borkowski T, Hoznek A, Salomon L, Gettman MT, Abbou CC. Laparoscopic radical prostatectomy in patients following transurethral resection of the prostate. Urol Int. 2006;77(3):216-21. doi:10.1159/000094812

366. Katz R, Salomon L, Hoznek A, de la Taille A, Antiphon P, Abbou CC. Positive surgical margins in laparoscopic radical prostatectomy: the impact of apical dissection, bladder neck remodeling and nerve preservation. J Urol. Jun 2003;169(6):2049-52. doi:10.1097/01.ju.0000065822.15012.b7

367. Kaufman MR, Smith JA, Baumgartner RG, et al. Positive influence of robotically assisted laparoscopic prostatectomy on the collaborative-care pathway for open radical prostatectomy. BJU Int. Mar 2006;97(3):473-5. doi:10.1111/j.1464-410X.2005.05993.x

368. Kaul S, Sammon J, Bhandari A, Peabody J, Rogers CG, Menon M. A novel method of urethrovesical anastomosis during robot-assisted radical prostatectomy using a unidirectional barbed wound closure device: feasibility study and early outcomes in 51 patients. J Endourol. Nov 2010;24(11):1789-93. doi:10.1089/end.2010.0200

369. Kaul S, Savera A, Badani K, Fumo M, Bhandari A, Menon M. Functional outcomes and oncological efficacy of Vattikuti Institute prostatectomy with Veil of Aphrodite nerve-sparing: an analysis of 154 consecutive patients. BJU Int. Mar 2006;97(3):467-72. doi:10.1111/j.1464-410X.2006.05990.x

370. Kawamorita N, Saito S, Ishidoya S, et al. Radical prostatectomy for high-risk prostate cancer: biochemical outcome. Int J Urol. Sep 2009;16(9):733-8. doi:10.1111/j.1442-2042.2009.02352.x

371. Kaye KW, Creed KE, Wilson GJ, D'Antuono M, Dawkins HJ. Urinary continence after radical retropubic prostatectomy. Analysis and synthesis of contributing factors: a unified concept. Br J Urol. Sep 1997;80(3):444-501. doi:10.1046/j.1464-410x.1997.00373.x

372. Kermarrec I, Mangin P, Koutlidis N, Mourey E, Cormier L. [Does robotics improve laparoscopic radical prostatectomy in complex surgical cases?]. Prog Urol. Oct 2010;20(9):638-43. doi:10.1016/j.purol.2010.03.002

373. Khaira HS, Bruyere F, O'Malley PJ, Peters JS, Costello AJ. Does obesity influence the operative course or complications of robot-assisted laparoscopic prostatectomy. BJU Int. Dec 2006;98(6):1275-8; discussion 1278. doi:10.1111/j.1464-410X.2006.06488.x

374. Khoder WY, Schlenker B, Waidelich R, et al. Open complete intrafascial nerve-sparing retropubic radical prostatectomy: technique and initial experience. Urology. Mar 2012;79(3):717-21. doi:10.1016/j.urology.2011.11.045

375. Khoder WY, Waidelich R, Buchner A, Becker AJ, Stief CG. Prospective comparison of one year follow-up outcomes for the open complete intrafascial retropubic versus interfascial nerve-sparing radical prostatectomy. Springerplus. 2014;3:335. doi:10.1186/2193-1801-3-335

376. Khoder WY, Waidelich R, Seitz M, et al. Do we need the nerve sparing radical prostatectomy techniques (intrafascial vs. interfascial) in men with erectile dysfunction? Results of a single-centre study. World J Urol. Mar 2015;33(3):301-7. doi:10.1007/s00345-014-1302-9

377. Kim IY, Hwang EA, Mmeje C, Ercolani M, Lee DH. Impact of posterior urethral plate repair on continence following robot-assisted laparoscopic radical prostatectomy. Yonsei Med J. May 2010;51(3):427-31. doi:10.3349/ymj.2010.51.3.427

378. Kim KH, Lim SK, Kim HY, et al. Extended vs standard lymph node dissection in robot-assisted radical prostatectomy for intermediate- or high-risk prostate cancer: a propensity-score-matching analysis. BJU Int. Jul 2013;112(2):216-23. doi:10.1111/j.1464-410X.2012.11765.x

379. Kim MJ, Park SY, Rha KH. Influence of prostate weight, obesity and height on surgical outcomes of robot-assisted laparoscopic radical prostatectomy in Korean men. J Robot Surg. 2008;1(4):287-90. doi:10.1007/s11701-007-0057-3

380. Kim SC, Song C, Kim W, et al. Factors determining functional outcomes after radical prostatectomy: robot-assisted versus retropubic. Eur Urol. Sep 2011;60(3):413-9. doi:10.1016/j.eururo.2011.05.011

381. Kim SD, Kim TH, Cho JW, You YC, Sung GT. Effect of Posterior Urethral Reconstruction (PUR) in Early Recovery of Urinary Continence after Robotic-Assisted Radical Prostatectomy. Korean J Urol. 12/ 2009;50(12):1203-1207.

382. Kim Y-J, Han BK, Byun S-S, Lee SE. Comparison of Perioperative Outcomes of Extraperitoneal Laparoscopic Radical Prostatectomy (ELRP) versus Open Radical Retropubic Prostatectomy (RRP): Single Surgeon's Initial Experience. Korean J Urol. 2/ 2007;48(2):131-137.

383. Kinoshita H, Nakagawa K, Usui Y, et al. High-definition resolution three-dimensional imaging systems in laparoscopic radical prostatectomy: randomized comparative study with high-definition resolution two-dimensional systems. Surg Endosc. Aug 2015;29(8):2203-9. doi:10.1007/s00464-014-3925-8

384. Klein EA, Bianco FJ, Serio AM, et al. Surgeon experience is strongly associated with biochemical recurrence after radical prostatectomy for all preoperative risk categories. J Urol. Jun 2008;179(6):2212-6; discussion 2216-7. doi:10.1016/j.juro.2008.01.107

385. Kleinclauss FM, Neuzillet Y, Tillou X, et al. Morbidity of retropubic radical prostatectomy for prostate cancer in renal transplant recipients: multicenter study from Renal Transplantation Committee of French Urological Association. Urology. Dec 2008;72(6):1366-70. doi:10.1016/j.urology.2008.03.018

386. Klevecka V, Burmester L, Musch M, Roggenbuck U, Kroepfl D. Intraoperative and early postoperative complications of radical retropubic prostatectomy. Urol Int. 2007;79(3):217-25. doi:10.1159/000107953

387. Ko WJ, Hruby GW, Turk AT, Landman J, Badani KK. Pathological confirmation of nerve-sparing types performed during robot-assisted radical prostatectomy (RARP). BJU Int. Mar 2013;111(3):451-8. doi:10.1111/j.1464-410X.2012.11393.x

388. Ko YH, Coelho RF, Chauhan S, et al. Factors affecting return of continence 3 months after robot-assisted radical prostatectomy: analysis from a large, prospective data by a single surgeon. J Urol. Jan 2012;187(1):190-4. doi:10.1016/j.juro.2011.09.037

389. Koehler N, Gansera L, Stolzenburg JU, et al. Early continence in patients with localized prostate cancer. A comparison between open retropubic (RRPE) and endoscopic extraperitoneal radical prostatectomy (EERPE). Urol Oncol. 2012 Nov-Dec 2012;30(6):798-803. doi:10.1016/j.urolonc.2010.10.013

390. Koliakos N, Mottrie A, Buffi N, De Naeyer G, Willemsen P, Fonteyne E. Posterior and anterior fixation of the urethra during robotic prostatectomy improves early continence rates. Scand J Urol Nephrol. Feb 2010;44(1):5-10. doi:10.3109/00365590903413627

391. Koo KC, Tuliao P, Yoon YE, et al. Robot-assisted radical prostatectomy in the Korean population: a 5-year propensity-score matched comparative analysis versus open radical prostatectomy. Int J Urol. Aug 2014;21(8):781-5. doi:10.1111/iju.12447

392. Kordan Y, Barocas DA, Altamar HO, et al. Comparison of transfusion requirements between open and robotic-assisted laparoscopic radical prostatectomy. BJU Int. Oct 2010;106(7):1036-40. doi:10.1111/j.1464-410X.2010.09233.x

393. Kouba E, Hubbard JS, Moore D, Wallen EM, Pruthi RS. A prospective evaluation of the short-term impact and recovery of health-related quality of life in men undergoing radical prostatectomy. BJU Int. Jan 2007;99(1):72-6. doi:10.1111/j.1464-410X.2007.06533.x

394. Koutlidis N, Duperron C, de la Vega MF, Mourey E, Michel F, Cormier L. Capsular incision in normal prostatic tissue during robot-assisted radical prostatectomy: a new concept or a waste of time? World J Urol. Oct 2014;32(5):1235-40. doi:10.1007/s00345-013-1199-8

395. Koutlidis N, Mourey E, Champigneulle J, Mangin P, Cormier L. Robot-assisted or pure laparoscopic nerve-sparing radical prostatectomy: what is the optimal procedure for the surgical margins? A single center experience. Int J Urol. Dec 2012;19(12):1076-81. doi:10.1111/j.1442-2042.2012.03102.x

396. Kowalczyk KJ, Huang AC, Hevelone ND, et al. Stepwise approach for nerve sparing without countertraction during robot-assisted radical prostatectomy: technique and outcomes. Eur Urol. Sep 2011;60(3):536-47. doi:10.1016/j.eururo.2011.05.001

397. Kowalczyk KJ, Levy JM, Caplan CF, et al. Temporal national trends of minimally invasive and retropubic radical prostatectomy outcomes from 2003 to 2007: results from the 100% Medicare sample. Eur Urol. Apr 2012;61(4):803-9. doi:10.1016/j.eururo.2011.12.020

398. Kozal S, Peyronnet B, Cattarino S, et al. Influence of pathological factors on oncological outcomes after robot-assisted radical prostatectomy for localized prostate cancer: Results of a prospective study. Urol Oncol. Jul 2015;33(7):330.e1-7. doi:10.1016/j.urolonc.2015.03.020

399. Krambeck AE, DiMarco DS, Rangel LJ, et al. Radical prostatectomy for prostatic adenocarcinoma: a matched comparison of open retropubic and robot-assisted techniques. BJU Int. Feb 2009;103(4):448-53. doi:10.1111/j.1464-410X.2008.08012.x

400. Krane LS, Bhandari M, Peabody JO, Menon M. Impact of percutaneous suprapubic tube drainage on patient discomfort after radical prostatectomy. Eur Urol. Aug 2009;56(2):325-30. doi:10.1016/j.eururo.2009.04.018

401. Krane LS, Wambi C, Bhandari A, Stricker HJ. Posterior support for urethrovesical anastomosis in robotic radical prostatectomy: single surgeon analysis. Can J Urol. Oct 2009;16(5):4836-40.

402. Ku JH, Jeong CW, Park YH, Cho MC, Kwak C, Kim HH. Nerve-sparing procedure in radical prostatectomy: a risk factor for hernia repair following open retropubic, pure laparoscopic and robot-assisted laparoscopic procedures. Scand J Urol Nephrol. Apr 2011;45(3):164-70. doi:10.3109/00365599.2010.544674

403. Ku JY, Ha HK. Learning curve of robot-assisted laparoscopic radical prostatectomy for a single experienced surgeon: comparison with simultaneous laparoscopic radical prostatectomy. World J Mens Health. Apr 2015;33(1):30-5. doi:10.5534/wjmh.2015.33.1.30

404. Ku TS, Kane CJ, Sen S, Henderson WG, Dudley RA, Cason BA. Effects of hospital procedure volume and resident training on clinical outcomes and resource use in radical retropubic prostatectomy surgery in the Department of Veterans Affairs. J Urol. Jan 2008;179(1):272-8; discussion 278-9. doi:10.1016/j.juro.2007.08.149

405. Kundu SD, Roehl KA, Eggener SE, Antenor JA, Han M, Catalona WJ. Potency, continence and complications in 3,477 consecutive radical retropubic prostatectomies. J Urol. Dec 2004;172(6 Pt 1):2227-31. doi:10.1097/01.ju.0000145222.94455.73

406. Kurokawa S, Umemoto Y, Mizuno K, et al. New steps of robot-assisted radical prostatectomy using the extraperitoneal approach: a propensity-score matched comparison between extraperitoneal and transperitoneal approach in Japanese patients. BMC Urol. Nov 21 2017;17(1):106. doi:10.1186/s12894-017-0298-z

407. Kwon EO, Bautista TC, Jung H, Goharderakhshan RZ, Williams SG, Chien GW. Impact of robotic training on surgical and pathologic outcomes during robot-assisted laparoscopic radical prostatectomy. Urology. Aug 2010;76(2):363-8. doi:10.1016/j.urology.2009.09.085

408. Kwon YS, Han CS, Yu JW, et al. Neutrophil and Lymphocyte Counts as Clinical Markers for Stratifying Low-Risk Prostate Cancer. Clin Genitourin Cancer. Feb 2016;14(1):e1-8. doi:10.1016/j.clgc.2015.07.018

409. Lallas CD, Pe ML, Patel JV, Sharma P, Gomella LG, Trabulsi EJ. Transperitoneal robotic-assisted laparoscopic prostatectomy after prosthetic mesh herniorrhaphy. JSLS. 2009 Apr-Jun 2009;13(2):142-7.

410. Lama M, Salinas N, Martinez J, Gribbell R, Cabrera O, Sudy C. Prospective study and comparative of surgical and oncologic outcome between laparoscopic and retropubical radical prostatectomy. Actas Urol Esp. 2009;33(2):167-71.

411. Lasser MS, Renzulli J, Turini GA, Haleblian G, Sax HC, Pareek G. An unbiased prospective report of perioperative complications of robot-assisted laparoscopic radical prostatectomy. Urology. May 2010;75(5):1083-9. doi:10.1016/j.urology.2009.09.082

412. Laungani RG, Kaul S, Muhletaler F, Badani KK, Peabody J, Menon M. Impact of previous inguinal hernia repair on transperitoneal robotic prostatectomy. Can J Urol. Aug 2007;14(4):3635-9.

413. Laurila TA, Huang W, Jarrard DF. Robotic-assisted laparoscopic and radical retropubic prostatectomy generate similar positive margin rates in low and intermediate risk patients. Urol Oncol. 2009 Sep-Oct 2009;27(5):529-33. doi:10.1016/j.urolonc.2008.05.001

414. Lavery HJ, Nabizada-Pace F, Carlucci JR, Brajtbord JS, Samadi DB. Nerve-sparing robotic prostatectomy in preoperatively high-risk patients is safe and efficacious. Urol Oncol. 2012 Jan-Feb 2012;30(1):26-32. doi:10.1016/j.urolonc.2009.11.023

415. Le JD, Cooperberg MR, Sadetsky N, et al. Changes in specific domains of sexual function and sexual bother after radical prostatectomy. BJU Int. Oct 2010;106(7):1022-9. doi:10.1111/j.1464-410X.2010.09231.x

416. Leandri P, Rossignol G, Gautier JR, Ramon J. Radical retropubic prostatectomy: morbidity and quality of life. Experience with 620 consecutive cases. J Urol. Mar 1992;147(3 Pt 2):883-7. doi:10.1016/s0022-5347(17)37412-8

417. Lebeau T, Rouprêt M, Ferhi K, et al. Assessing the complications of laparoscopic robot-assisted surgery: the case of radical prostatectomy. Surg Endosc. Feb 2011;25(2):536-42. doi:10.1007/s00464-010-1210-z

418. Lee BC, Rodin DM, Shah KK, Dahl DM. Laparoscopic inguinal hernia repair during laparoscopic radical prostatectomy. BJU Int. Mar 2007;99(3):637-9. doi:10.1111/j.1464-410X.2006.06687.x

419. Lee CH, Ha HK. Intravesical prostatic protrusion as a predictor of early urinary continence recovery after laparoscopic radical prostatectomy. Int J Urol. Jul 2014;21(7):653-6. doi:10.1111/iju.12419

420. Lee DH, Jung HB, Chung MS, Lee SH, Chung BH. Patent processus vaginalis in adults who underwent robot-assisted laparoscopic radical prostatectomy: predictive signs of postoperative inguinal hernia in the internal inguinal floor. Int J Urol. Feb 2013;20(2):177-82. doi:10.1111/j.1442-2042.2012.03118.x

421. Lee DH, Koo KC, Lee SH, Chung BH. A simple procedure to prevent postoperative inguinal hernia after robot-assisted laparoscopic radical prostatectomy: a plugging method of the internal inguinal floor for patients with patent processus vaginalis. J Urol. Feb 2014;191(2):468-72. doi:10.1016/j.juro.2013.09.035

422. Lee DJ, Cheetham P, Badani KK. Penile rehabilitation protocol after robot-assisted radical prostatectomy: assessment of compliance with phosphodiesterase type 5 inhibitor therapy and effect on early potency. BJU Int. Feb 2010;105(3):382-8. doi:10.1111/j.1464-410X.2009.08820.x

423. Lee DJ, Cheetham P, Badani KK. Predictors of early urinary continence after robotic prostatectomy. Can J Urol. Jun 2010;17(3):5200-5.

424. Lee DK, Montgomery DP, Porter JR. Concurrent transperitoneal repair for incidentally detected inguinal hernias during robotically assisted radical prostatectomy. Urology. Dec 2013;82(6):1320-2. doi:10.1016/j.urology.2013.08.028

425. Lee D, Choi SK, Park J, et al. Comparative analysis of oncologic outcomes for open vs. robot-assisted radical prostatectomy in high-risk prostate cancer. Korean J Urol. Aug 2015;56(8):572-9. doi:10.4111/kju.2015.56.8.572

426. Lee H, Kim K, Hwang SI, et al. Impact of prostatic apical shape and protrusion on early recovery of continence after robot-assisted radical prostatectomy. Urology. Oct 2014;84(4):844-9. doi:10.1016/j.urology.2014.06.011

427. Lee HW, Lee HM, Seo SI. Comparison of Initial Surgical Outcomes between Laparoscopic Radical Prostatectomy and Robot-Assisted Laparoscopic Radical Prostatectomy Performed by a Single Surgeon. Korean J Urol. 5/ 2009;50(5):468-474.

428. Lee JW, Jeong WJ, Park SY, Loreazo EI, Oh CK, Rha KH. Learning curve for robot-assisted laparoscopic radical prostatectomy for pathologic t2 disease. Korean J Urol. Jan 2010;51(1):30-3. doi:10.4111/kju.2010.51.1.30

429. Lee SH, Chung MS, Chung YG, Park KK, Chung BH. Does performance of robot-assisted laparoscopic radical prostatectomy within 2 weeks of prostate biopsy affect the outcome? Int J Urol. Feb 2011;18(2):141-6. doi:10.1111/j.1442-2042.2010.02675.x

430. Lee SW, Han DH, Lee K-S, Jeon SS. Effect of Continuous Urethro-Vesical Anastomosis Technique in Incontinence After Radical Retropubic Prostatectomy, 1:1 Matching Study. International neurourology journal. 2015;19(2):113-119. doi:10.5213/inj.2015.19.2.113

431. Lee Z, Sehgal SS, Graves RV, et al. Functional and oncologic outcomes of graded bladder neck preservation during robot-assisted radical prostatectomy. J Endourol. Jan 2014;28(1):48-55. doi:10.1089/end.2013.0290

432. Leewansangtong S, Wiangsakunna W, Taweemankongsap T. Perioperative outcomes of open radical prostatectomy versus laparoscopic radical prostatectomy in Asian men: comparison of two initial series by the same surgeon. Int Braz J Urol. 2009 Mar-Apr 2009;35(2):151-6; discussion 156-7.

433. Lei Y, Alemozaffar M, Williams SB, et al. Athermal division and selective suture ligation of the dorsal vein complex during robot-assisted laparoscopic radical prostatectomy: description of technique and outcomes. Eur Urol. Feb 2011;59(2):235-43. doi:10.1016/j.eururo.2010.08.043

434. Leibman BD, Dillioglugil O, Abbas F, Tanli S, Kattan MW, Scardino PT. Impact of a clinical pathway for radical retropubic prostatectomy. Urology. Jul 1998;52(1):94-9. doi:10.1016/s0090-4295(98)00130-7

435. Lein M, Stibane I, Mansour R, et al. Complications, urinary continence, and oncologic outcome of 1000 laparoscopic transperitoneal radical prostatectomies-experience at the Charité Hospital Berlin, Campus Mitte. Eur Urol. Dec 2006;50(6):1278-82; discussion 1283-4. doi:10.1016/j.eururo.2006.06.023

436. Lepor H, Kaci L. The impact of open radical retropubic prostatectomy on continence and lower urinary tract symptoms: a prospective assessment using validated self-administered outcome instruments. J Urol. Mar 2004;171(3):1216-9. doi:10.1097/01.ju.0000113964.68020.a7

437. Lepor H, Lipkin M, Slova D. The preoperative use of erythropoietin stimulating proteins prior to radical prostatectomy is not associated with increased cardiovascular or thromboembolic morbidity or mortality. Urology. Jun 2010;75(6):1424-8. doi:10.1016/j.urology.2009.06.095

438. Lepor H, Nieder AM, Ferrandino MN. Intraoperative and postoperative complications of radical retropubic prostatectomy in a consecutive series of 1,000 cases. J Urol. Nov 2001;166(5):1729-33.

439. Leroy TJ, Thiel DD, Duchene DA, et al. Safety and peri-operative outcomes during learning curve of robot-assisted laparoscopic prostatectomy: a multi-institutional study of fellowship-trained robotic surgeons versus experienced open radical prostatectomy surgeons incorporating robot-assisted laparoscopic prostatectomy. J Endourol. Oct 2010;24(10):1665-9. doi:10.1089/end.2009.0657

440. Leung RA, Kim TS, Tewari AK. Future directions of robotic surgery: a case study of the Cornell athermal robotic technique of prostatectomy. ScientificWorldJournal. Mar 2006;6:2553-9. doi:10.1100/tsw.2006.395

441. Levinson AW, Lavery HJ, Ward NT, Su LM, Pavlovich CP. Is a return to baseline sexual function possible? An analysis of sexual function outcomes following laparoscopic radical prostatectomy. World J Urol. Feb 2011;29(1):29-34. doi:10.1007/s00345-010-0616-5

442. Levinson AW, Ward NT, Sulman A, et al. The impact of prostate size on perioperative outcomes in a large laparoscopic radical prostatectomy series. J Endourol. Jan 2009;23(1):147-52. doi:10.1089/end.2008.0366

443. Leyh-Bannurah SR, Hansen J, Isbarn H, et al. Open and robot-assisted radical retropubic prostatectomy in men receiving ongoing low-dose aspirin medication: revisiting an old paradigm? BJU Int. Sep 2014;114(3):396-403. doi:10.1111/bju.12504

444. Li B, Suzuki K, Tsuru N, Ushiyama T, Ozono S. Retrospective comparative study of 59 cases of laparoscopic radical prostatectomy: transperitoneal anterior versus transperitoneal posterior approach. Int J Urol. Nov 2007;14(11):1005-8. doi:10.1111/j.1442-2042.2007.01878.x

445. Liao X, Qiao P, Tan Z, Shi H, Xing N. "Total reconstruction" of the urethrovesical anastomosis contributes to early urinary continence in laparoscopic radical prostatectomy. Int Braz J Urol. 2016 Mar-Apr 2016;42(2):215-22.

446. Liatsikos E, Rabenalt R, Burchardt M, et al. Prevention and management of perioperative complications in laparoscopic and endoscopic radical prostatectomy. World J Urol. Dec 2008;26(6):571-80. doi:10.1007/s00345-008-0328-2

447. Licht MR, Klein EA, Tuason L, Levin H. Impact of bladder neck preservation during radical prostatectomy on continence and cancer control. Urology. Dec 1994;44(6):883-7. doi:10.1016/s0090-4295(94)80175-4

448. Lim JH, Park CM, Kim HK, Park JY. Comparison of perioperative outcomes between running versus interrupted vesicourethral anastomosis in open radical prostatectomy: A single-surgeon experience. Korean J Urol. 6/ 2015;56(6):443-448.

449. Lim SK, Kim KH, Shin TY, et al. Retzius-sparing robot-assisted laparoscopic radical prostatectomy: combining the best of retropubic and perineal approaches. BJU Int. Aug 2014;114(2):236-44. doi:10.1111/bju.12705

450. Lim TJ, Lee JH, Lim JW, Moon SK, Jeon SH, Chang SG. Preoperative factors predictive of continence recovery after radical retropubic prostatectomy. Korean J Urol. Aug 2012;53(8):524-30. doi:10.4111/kju.2012.53.8.524

451. Lin BM, Hyndman ME, Steele KE, et al. Incidence and risk factors for inguinal and incisional hernia after laparoscopic radical prostatectomy. Urology. Apr 2011;77(4):957-62. doi:10.1016/j.urology.2010.12.011

452. Lindner U, Lawrentschuk N, Abouassaly R, Fleshner NE, Trachtenberg J. Radical prostatectomy in obese patients: Improved surgical outcomes in recent years. Int J Urol. Aug 2010;17(8):727-32. doi:10.1111/j.1442-2042.2010.02570.x

453. Link BA, Nelson R, Josephson DY, Lau C, Wilson TG. Training of urologic oncology fellows does not adversely impact outcomes of robot-assisted laparoscopic prostatectomy. J Endourol. Feb 2009;23(2):301-5. doi:10.1089/end.2008.0378

454. Link BA, Nelson R, Josephson DY, et al. The impact of prostate gland weight in robot assisted laparoscopic radical prostatectomy. J Urol. Sep 2008;180(3):928-32. doi:10.1016/j.juro.2008.05.029

455. Link RE, Su LM, Bhayani SB, Pavlovich CP. Making ends meet: a cost comparison of laparoscopic and open radical retropubic prostatectomy. J Urol. Jul 2004;172(1):269-74. doi:10.1097/01.ju.0000128773.99707.5b

456. Link RE, Su LM, Sullivan W, Bhayani SB, Pavlovich CP. Health related quality of life before and after laparoscopic radical prostatectomy. J Urol. Jan 2005;173(1):175-9; discussion 179. doi:10.1097/01.ju.0000147190.67218.1b

457. Liss MA, Palazzi K, Stroup SP, Jabaji R, Raheem OA, Kane CJ. Outcomes and complications of pelvic lymph node dissection during robotic-assisted radical prostatectomy. World J Urol. Jun 2013;31(3):481-8. doi:10.1007/s00345-013-1056-9

458. Liss M, Osann K, Ornstein D. Positive surgical margins during robotic radical prostatectomy: a contemporary analysis of risk factors. BJU Int. Aug 2008;102(5):603-8. doi:10.1111/j.1464-410X.2008.07672.x

459. Litwiller SE, Djavan B, Klopukh BV, Richier JC, Roehrborn CG. Radical retropubic prostatectomy for localized carcinoma of the prostate in a large metropolitan hospital: changing trends over a 10-year period (1984-1994). Dallas Outcomes Research Group for Urological Disorders. Urology. May 1995;45(5):813-22. doi:10.1016/s0090-4295(99)80089-2

460. Lo KL, Ng CF, Lam CN, Hou SS, To KF, Yip SK. Short-term outcome of patients with robot-assisted versus open radical prostatectomy: for localised carcinoma of prostate. Hong Kong Med J. Feb 2010;16(1):31-5.

461. Loeb S, Epstein JI, Ross AE, Schultz L, Humphreys EB, Jarow JP. Benign prostate glands at the bladder neck margin in robotic vs open radical prostatectomy. BJU Int. May 2010;105(10):1446-9. doi:10.1111/j.1464-410X.2010.09336.x

462. Loeb S, Roehl KA, Helfand BT, Catalona WJ. Complications of open radical retropubic prostatectomy in potential candidates for active monitoring. Urology. Oct 2008;72(4):887-91. doi:10.1016/j.urology.2007.12.016

463. Löppenberg B, Noldus J, Holz A, Palisaar RJ. Reporting complications after open radical retropubic prostatectomy using the Martin criteria. J Urol. Sep 2010;184(3):944-8. doi:10.1016/j.juro.2010.05.032

464. Louie-Johnsun MW, Handmer MM, Calopedos RJ, et al. The Australian laparoscopic non robotic radical prostatectomy experience - analysis of 2943 cases (USANZ supplement). BJU Int. Oct 2016;118 Suppl 3:43-48. doi:10.1111/bju.13610

465. Louie-Johnsun M, Ouyang R, Indrajit B, Haque M. Laparoscopic radical prostatectomy: introduction of training during our first 50 cases. ANZ J Surg. Mar 2012;82(3):131-5. doi:10.1111/j.1445-2197.2011.05986.x

466. Lowrance WT, Elkin EB, Jacks LM, et al. Comparative effectiveness of prostate cancer surgical treatments: a population based analysis of postoperative outcomes. J Urol. Apr 2010;183(4):1366-72. doi:10.1016/j.juro.2009.12.021

467. Luciani LG, Mattevi D, Mantovani W, et al. Retropubic, Laparoscopic, and Robot-Assisted Radical Prostatectomy: A Comparative Analysis of the Surgical Outcomes in a Single Regional Center. Curr Urol. Nov 2017;11(1):36-41. doi:10.1159/000447192

468. Ludovico GM, Dachille G, Pagliarulo G, et al. Bilateral nerve sparing robotic-assisted radical prostatectomy is associated with faster continence recovery but not with erectile function recovery compared with retropubic open prostatectomy: the need for accurate selection of patients. Oncol Rep. Jun 2013;29(6):2445-50. doi:10.3892/or.2013.2365

469. Lukasewycz S, Holman M, Kozlowski P, et al. Does a perioperative belladonna and opium suppository improve postoperative pain following robotic assisted laparoscopic radical prostatectomy? Results of a single institution randomized study. Can J Urol. Oct 2010;17(5):5377-82.

470. Luke S, Delprado W, Louie-Johnsun M. Teaching laparoscopic radical prostatectomy during the primary surgeon's early learning curve--analysis of our first 207 cases. BJU Int. Nov 2014;114 Suppl 1:38-44. doi:10.1111/bju.12799

471. Madeb R, Golijanin D, Knopf J, et al. Patient-reported validated functional outcome after extraperitoneal robotic-assisted nerve-sparing radical prostatectomy. JSLS. 2007 Oct-Dec 2007;11(4):443-8.

472. Madi R, Daignault S, Wood DP. Extraperitoneal v intraperitoneal robotic prostatectomy: analysis of operative outcomes. J Endourol. Dec 2007;21(12):1553-7. doi:10.1089/end.2007.9872

473. Maffezzini M, Seveso M, Taverna G, Giusti G, Benetti A, Graziotti P. Evaluation of complications and results in a contemporary series of 300 consecutive radical retropubic prostatectomies with the anatomic approach at a single institution. Urology. May 2003;61(5):982-6. doi:10.1016/s0090-4295(02)02517-7

474. Magera JS, Inman BA, Slezak JM, Bagniewski SM, Sebo TJ, Myers RP. Increased optical magnification from 2.5x to 4.3x with technical modification lowers the positive margin rate in open radical retropubic prostatectomy. J Urol. Jan 2008;179(1):130-5. doi:10.1016/j.juro.2007.08.128

475. Magheli A, Busch J, Leva N, et al. Comparison of surgical technique (open vs. laparoscopic) on pathological and long term functional outcomes following radical prostatectomy. BMC Urol. Feb 2014;14:18. doi:10.1186/1471-2490-14-18

476. Magheli A, Gonzalgo ML, Su LM, et al. Impact of surgical technique (open vs laparoscopic vs robotic-assisted) on pathological and biochemical outcomes following radical prostatectomy: an analysis using propensity score matching. BJU Int. Jun 2011;107(12):1956-62. doi:10.1111/j.1464-410X.2010.09795.x

477. Majima T, Yoshino Y, Matsukawa Y, et al. Causative factors for de novo inguinal hernia after robot-assisted radical prostatectomy. J Robot Surg. Jun 2018;12(2):277-282. doi:10.1007/s11701-017-0729-6

478. Malcolm JB, Fabrizio MD, Barone BB, et al. Quality of life after open or robotic prostatectomy, cryoablation or brachytherapy for localized prostate cancer. J Urol. May 2010;183(5):1822-8. doi:10.1016/j.juro.2009.12.102

479. Maldonado-Valadez R, Teber D, Erdogru T, Safi KC, Frede T, Rassweiler J. The impact of neoadjuvant hormonal therapy on the outcome of laparoscopic radical prostatectomy: a matched pair analysis. J Urol. Jun 2006;175(6):2092-6. doi:10.1016/S0022-5347(06)00260-6

480. Malik R, Laze J, Lepor H. The effect of local compression and topical epinephrine on perioperative bleeding and degree of urinary extravasation on postoperative cystogram following radical retropubic prostatectomy. Can J Urol. Aug 2010;17(4):5272-7.

481. Manassero F, Traversi C, Ales V, et al. Contribution of early intensive prolonged pelvic floor exercises on urinary continence recovery after bladder neck-sparing radical prostatectomy: results of a prospective controlled randomized trial. Neurourol Urodyn. 2007;26(7):985-9. doi:10.1002/nau.20442

482. Manferrari F, Brunocilla E, Baccos A, et al. Laparoscopic radical prostatectomy: 10 years of experience at a single institution. Anticancer Res. May 2014;34(5):2443-8.

483. Manganiello M, Kenney P, Canes D, Sorcini A, Moinzadeh A. Unidirectional barbed suture versus standard monofilament for urethrovesical anastomosis during robotic assisted laparoscopic radical prostatectomy. Int Braz J Urol. 2012 Jan-Feb 2012;38(1):89-96. doi:10.1590/s1677-55382012000100013

484. Manoharan M, Ayyathurai R, Nieder AM, Soloway MS. Modified Pfannenstiel approach for radical retropubic prostatectomy: a 3-year experience. Prostate Cancer Prostatic Dis. 2008;11(1):74-8. doi:10.1038/sj.pcan.4500969

485. Marchetti PE, Shikanov S, Razmaria AA, Zagaja GP, Shalhav AL. Impact of prostate weight on probability of positive surgical margins in patients with low-risk prostate cancer after robotic-assisted laparoscopic radical prostatectomy. Urology. Mar 2011;77(3):677-81. doi:10.1016/j.urology.2010.07.512

486. Mariano MB, Tefilli MV, Fonseca GN, Goldraich IH. Laparoscopic radical prostatectomy: 10 years experience. Int Braz J Urol. 2009 Sep-Oct 2009;35(5):565-71; discussion 571-2.

487. Marien TP, Lepor H. Does a nerve-sparing technique or potency affect continence after open radical retropubic prostatectomy? BJU Int. Dec 2008;102(11):1581-4. doi:10.1111/j.1464-410X.2008.07921.x

488. Marien T, Sankin A, Lepor H. Factors predicting preservation of erectile function in men undergoing open radical retropubic prostatectomy. J Urol. Apr 2009;181(4):1817-22. doi:10.1016/j.juro.2008.11.105

489. Martin AD, Desai PJ, Nunez RN, et al. Does a history of previous surgery or radiation to the prostate affect outcomes of robot-assisted radical prostatectomy? BJU Int. Jun 2009;103(12):1696-8. doi:10.1111/j.1464-410X.2008.08276.x

490. Martin AD, Nakamura LY, Nunez RN, Wolter CE, Humphreys MR, Castle EP. Incontinence after radical prostatectomy: a patient centered analysis and implications for preoperative counseling. J Urol. Jul 2011;186(1):204-8. doi:10.1016/j.juro.2011.02.2698

491. Martin GL, Nunez RN, Humphreys MD, et al. Interval from prostate biopsy to robot-assisted radical prostatectomy: effects on perioperative outcomes. BJU Int. Dec 2009;104(11):1734-7. doi:10.1111/j.1464-410X.2009.08685.x

492. Martina GR, Giumelli P, Scuzzarella S, Remotti M, Caruso G, Lovisolo J. Laparoscopic extraperitoneal radical prostatectomy--learning curve of a laparoscopy-naive urologist in a community hospital. Urology. May 2005;65(5):959-63. doi:10.1016/j.urology.2004.11.019

493. Martínez CH, Chalasani V, Lim D, et al. Effect of prostate gland size on the learning curve for robot-assisted laparoscopic radical prostatectomy: does size matter initially? J Endourol. Feb 2010;24(2):261-6. doi:10.1089/end.2009.0325

494. Martínez-Piñeiro L, Cáceres F, Sánchez C, et al. Learning Curve of Laparoscopic Radical Prostatectomy in a University Teaching Hospital: Experience after the First 600 Cases. European Urology Supplements. 2006;5(19):914-924. doi:10.1016/j.eursup.2006.07.018

495. Martinez-Salamanca JI, Ramanathan R, Rao S, et al. Second Prize: Pelvic neuroanatomy and innovative approaches to minimize nerve damage and maximize cancer control in patients undergoing robot-assisted radical prostatectomy. J Endourol. Jun 2008;22(6):1137-46. doi:10.1089/end.2008.0097

496. Martinschek A, Heinzelmann K, Ritter M, Heinrich E, Trojan L. Radical prostatectomy after previous transurethral resection of the prostate: robot-assisted laparoscopic versus open radical prostatectomy in a matched-pair analysis. J Endourol. Sep 2012;26(9):1136-41. doi:10.1089/end.2012.0074

497. Martinschek A, Pfalzgraf D, Rafail B, Ritter M, Heinrich E, Trojan L. Transurethral versus suprapubic catheter at robot-assisted radical prostatectomy: a prospective randomized trial with 1-year follow-up. World J Urol. Mar 2016;34(3):407-11. doi:10.1007/s00345-015-1678-1

498. Martis G, Diana M, Ombres M, Cardi A, Mastrangeli R, Mastrangeli B. Retropubic versus perineal radical prostatectomy in early prostate cancer: eight-year experience. J Surg Oncol. May 2007;95(6):513-8. doi:10.1002/jso.20714

499. Mason BM, Hakimi AA, Faleck D, Chernyak V, Rozenblitt A, Ghavamian R. The role of preoperative endo-rectal coil magnetic resonance imaging in predicting surgical difficulty for robotic prostatectomy. Urology. Nov 2010;76(5):1130-5. doi:10.1016/j.urology.2010.05.037

500. Mason S, Van Hemelrijck M, Chandra A, Brown C, Cahill D. Laparoscopic radical prostatectomy outcome data: how should surgeon's performance be reported? A retrospective learning curve analysis of two surgeons. Ecancermedicalscience. 2016;10:651. doi:10.3332/ecancer.2016.651

501. Massoud W, Thanigasalam R, El Hajj A, et al. Does the use of a barbed polyglyconate absorbable suture have an impact on urethral anastomosis time, urethral stenosis rates, and cost effectiveness during robot-assisted radical prostatectomy? Urology. Jul 2013;82(1):90-4. doi:10.1016/j.urology.2013.02.002

502. Masterson TA, Cheng L, Boris RS, Koch MO. Open vs. robotic-assisted radical prostatectomy: a single surgeon and pathologist comparison of pathologic and oncologic outcomes. Urol Oncol. Oct 2013;31(7):1043-8. doi:10.1016/j.urolonc.2011.12.002

503. Matsumoto R, Sakashita S. [Prospective study of extended versus limited lymphadenectomy in patients undergoing radical prostatectomy with localized prostate cancer]. Hinyokika Kiyo. Jul 2011;57(7):359-62.

504. Matsushima M, Miyajima A, Hattori S, et al. Comparison of continence outcomes of early catheter removal on postoperative day 2 and 4 after laparoscopic radical prostatectomy: a randomized controlled trial. BMC Urol. Jul 2015;15:77. doi:10.1186/s12894-015-0065-y

505. Matsuyama H, Matsumoto H, Nagao K, Harada N, Hara T, Sakano S. Running suture versus interrupted suture for vesicourethral anastomosis in retropubic radical prostatectomy: a randomized study. Int J Urol. Mar 2015;22(3):271-7. doi:10.1111/iju.12667

506. Mattei A, Naspro R, Annino F, Burke D, Guida R, Gaston R. Tension and energy-free robotic-assisted laparoscopic radical prostatectomy with interfascial dissection of the neurovascular bundles. Eur Urol. Sep 2007;52(3):687-94. doi:10.1016/j.eururo.2007.05.029

507. May M, Dorst M, May J, et al. Radical retropubic vs. radical perineal prostatectomy: a comparison of relative benefits in four urban hospitals. Urol Nurs. Dec 2007;27(6):519-26.

508. Mayer EK, Winkler MH, Aggarwal R, et al. Robotic prostatectomy: the first UK experience. Int J Med Robot. Dec 2006;2(4):321-8. doi:10.1002/rcs.113

509. Mazaris EM, Chatzidarellis E, Varkarakis IM, Dellis A, Deliveliotis C. Reducing the number of sutures for vesicourethral anastomosis in radical retropubic prostatectomy. Int Braz J Urol. 2009 Mar-Apr 2009;35(2):158-63. doi:10.1590/s1677-55382009000200005

510. Mazaris EM, Varkarakis I, Chrisofos M, et al. Use of nonsteroidal anti-inflammatory drugs after radical retropubic prostatectomy: a prospective, randomized trial. Urology. Dec 2008;72(6):1293-7. doi:10.1016/j.urology.2007.12.039

511. McNeill AS, Nabi G, McLornan L, Cook J, Bollina P, Stolzenberg JU. Endoscopic extraperitoneal radical prostatectomy: critical analysis of outcomes and learning curve. BJU Int. Nov 2010;106(10):1537-43. doi:10.1111/j.1464-410X.2010.09322.x

512. Menard J, de la Taille A, Hoznek A, et al. Laparoscopic radical prostatectomy after transurethral resection of the prostate: surgical and functional outcomes. Urology. Sep 2008;72(3):593-7. doi:10.1016/j.urology.2008.03.019

513. Mendiola FP, Zorn KC, Mikhail AA, et al. Urinary and sexual function outcomes among different age groups after robot-assisted laparoscopic prostatectomy. J Endourol. Mar 2008;22(3):519-24. doi:10.1089/end.2006.9845

514. M M, A T, JO P, et al. Vattikuti Institute prostatectomy, a technique of robotic radical prostatectomy for management of localized carcinoma of the prostate: experience of over 1100 cases. The Urologic clinics of North America. 2004 Nov 2004;31(4)doi:10.1016/j.ucl.2004.06.011

515. Menon M, Bhandari M, Gupta N, et al. Biochemical recurrence following robot-assisted radical prostatectomy: analysis of 1384 patients with a median 5-year follow-up. Eur Urol. Dec 2010;58(6):838-46. doi:10.1016/j.eururo.2010.09.010

516. Menon M, Dalela D, Jamil M, et al. Functional Recovery, Oncologic Outcomes and Postoperative Complications after Robot-Assisted Radical Prostatectomy: An Evidence-Based Analysis Comparing the Retzius Sparing and Standard Approaches. J Urol. May 2018;199(5):1210-1217. doi:10.1016/j.juro.2017.11.115

517. Menon M, Hemal AK, Team V. Vattikuti Institute prostatectomy: a technique of robotic radical prostatectomy: experience in more than 1000 cases. J Endourol. Sep 2004;18(7):611-9; discussion 619. doi:10.1089/end.2004.18.611

518. Menon M, Kaul S, Bhandari A, Shrivastava A, Tewari A, Hemal A. Potency following robotic radical prostatectomy: a questionnaire based analysis of outcomes after conventional nerve sparing and prostatic fascia sparing techniques. J Urol. Dec 2005;174(6):2291-6, discussion 2296. doi:10.1097/01.ju.0000181825.54480.eb

519. Menon M, Muhletaler F, Campos M, Peabody JO. Assessment of early continence after reconstruction of the periprostatic tissues in patients undergoing computer assisted (robotic) prostatectomy: results of a 2 group parallel randomized controlled trial. J Urol. Sep 2008;180(3):1018-23. doi:10.1016/j.juro.2008.05.046

520. Menon M, Shrivastava A, Bhandari M, Satyanarayana R, Siva S, Agarwal PK. Vattikuti Institute prostatectomy: technical modifications in 2009. Eur Urol. Jul 2009;56(1):89-96. doi:10.1016/j.eururo.2009.04.032

521. Menon M, Shrivastava A, Kaul S, et al. Vattikuti Institute prostatectomy: contemporary technique and analysis of results. Eur Urol. Mar 2007;51(3):648-57; discussion 657-8. doi:10.1016/j.eururo.2006.10.055

522. Menon M, Shrivastava A, Sarle R, Hemal A, Tewari A. Vattikuti Institute Prostatectomy: a single-team experience of 100 cases. J Endourol. Nov 2003;17(9):785-90. doi:10.1089/089277903770802380

523. Menon M, Shrivastava A, Tewari A. Laparoscopic radical prostatectomy: conventional and robotic. Urology. Nov 2005;66(5 Suppl):101-4. doi:10.1016/j.urology.2005.06.008

524. Menon M, Tewari A, Baize B, Guillonneau B, Vallancien G. Prospective comparison of radical retropubic prostatectomy and robot-assisted anatomic prostatectomy: the Vattikuti Urology Institute experience. Urology. Nov 2002;60(5):864-8. doi:10.1016/s0090-4295(02)01881-2

525. Menon M, Tewari A, Peabody J, Team V. Vattikuti Institute prostatectomy: technique. J Urol. Jun 2003;169(6):2289-92. doi:10.1097/01.ju.0000067464.53313.dd

526. Menon M, Tewari A, Team VIP. Robotic radical prostatectomy and the Vattikuti Urology Institute technique: an interim analysis of results and technical points. Urology. Apr 2003;61(4 Suppl 1):15-20. doi:10.1016/s0090-4295(03)00116-x

527. Merrilees AD, Bethwaite PB, Russell GL, Robinson RG, Delahunt B. Parameters of perineural invasion in radical prostatectomy specimens lack prognostic significance. Mod Pathol. Sep 2008;21(9):1095-100. doi:10.1038/modpathol.2008.81

528. Michl UH, Friedrich MG, Graefen M, Haese A, Heinzer H, Huland H. Prediction of postoperative sexual function after nerve sparing radical retropubic prostatectomy. J Urol. Jul 2006;176(1):227-31. doi:10.1016/S0022-5347(06)00632-X

529. Mikhail AA, Orvieto MA, Billatos ES, et al. Robotic-assisted laparoscopic prostatectomy: first 100 patients with one year of follow-up. Urology. Dec 2006;68(6):1275-9. doi:10.1016/j.urology.2006.08.1060

530. Mikhail AA, Stockton BR, Orvieto MA, et al. Robotic-assisted laparoscopic prostatectomy in overweight and obese patients. Urology. Apr 2006;67(4):774-9. doi:10.1016/j.urology.2005.10.049

531. Miller J, Smith A, Kouba E, Wallen E, Pruthi RS. Prospective evaluation of short-term impact and recovery of health related quality of life in men undergoing robotic assisted laparoscopic radical prostatectomy versus open radical prostatectomy. J Urol. Sep 2007;178(3 Pt 1):854-8; discussion 859. doi:10.1016/j.juro.2007.05.051

532. Minniti D, Chiadò Piat S, Di Novi C. Robot-assisted versus open radical prostatectomy: an evidence-based comparison. Technol Health Care. 2011;19(5):331-9. doi:10.3233/THC-2011-0635

533. Mistretta FA, Galfano A, Di Trapani E, et al. Robot assisted radical prostatectomy in kidney transplant recipients: surgical, oncological and functional outcomes of two different robotic approaches. Int Braz J Urol. Mar-Apr 2019;45(2):262-272. doi:10.1590/s1677-5538.ibju.2018.0308

534. Mitre AI, Chammas MF, Rocha JE, Duarte RJ, Ebaid GX, Rocha FT. Laparoscopic radical prostatectomy: the learning curve of a low volume surgeon. ScientificWorldJournal. 2013;2013:974276. doi:10.1155/2013/974276

535. Miyake H, Fujimoto H, Komiyama M, Fujisawa M. Development of "extended radical retropubic prostatectomy": a surgical technique for improving margin positive rates in prostate cancer. Eur J Surg Oncol. Mar 2010;36(3):281-6. doi:10.1016/j.ejso.2009.10.013

536. Morgan MS, Ozayar A, Friedlander JI, et al. An Assessment of Patient Comfort and Morbidity After Robot-Assisted Radical Prostatectomy with Suprapubic Tube Versus Urethral Catheter Drainage. J Endourol. Mar 2016;30(3):300-5. doi:10.1089/end.2015.0206

537. Mortezavi A, Hermanns T, Hefermehl LJ, et al. Continuous low-dose aspirin therapy in robotic-assisted laparoscopic radical prostatectomy does not increase risk of surgical hemorrhage. J Laparoendosc Adv Surg Tech A. Jun 2013;23(6):500-5. doi:10.1089/lap.2013.0013

538. Moskovic DJ, Lavery HJ, Rehman J, Nabizada-Pace F, Brajtbord J, Samadi DB. High body mass index does not affect outcomes following robotic assisted laparoscopic prostatectomy. Can J Urol. Aug 2010;17(4):5291-8.

539. Mottrie A, Van Migem P, De Naeyer G, Schatteman P, Carpentier P, Fonteyne E. Robot-assisted laparoscopic radical prostatectomy: oncologic and functional results of 184 cases. Eur Urol. Sep 2007;52(3):746-50. doi:10.1016/j.eururo.2007.02.029

540. Mouraviev V, Nosnik I, Sun L, et al. Financial comparative analysis of minimally invasive surgery to open surgery for localized prostate cancer: a single-institution experience. Urology. Feb 2007;69(2):311-4. doi:10.1016/j.urology.2006.10.025

541. Mourmouris P, Argun OB, Tufek I, et al. Nonprosthetic Direct Inguinal Hernia Repair During Robotic Radical Prostatectomy. J Endourol. Feb 2016;30(2):218-22. doi:10.1089/end.2015.0556

542. Msezane LP, Reynolds WS, Gofrit ON, Shalhav AL, Zagaja GP, Zorn KC. Bladder neck contracture after robot-assisted laparoscopic radical prostatectomy: evaluation of incidence and risk factors and impact on urinary function. J Endourol. Jan 2008;22(1):97-104.

543. Murphy DG, Kerger M, Crowe H, Peters JS, Costello AJ. Operative details and oncological and functional outcome of robotic-assisted laparoscopic radical prostatectomy: 400 cases with a minimum of 12 months follow-up. Eur Urol. Jun 2009;55(6):1358-66. doi:10.1016/j.eururo.2008.12.035

544. Nadler RB, Casey JT, Zhao LC, et al. Is the transition from open to robotic prostatectomy fair to your patients? A single-surgeon comparison with 2-year follow-up. J Robot Surg. Jan 2010;3(4):201-7. doi:10.1007/s11701-009-0162-6

545. Nadu A, Salomon L, Hoznek A, et al. Early removal of the catheter after laparoscopic radical prostatectomy. J Urol. Nov 2001;166(5):1662-4.

546. Nakamura K, Kasraeian A, Yacoub S, Pendleton J, Anai S, Rosser CJ. The use of enoxaparin to prevent venous thromboembolism in patients undergoing radical retropubic prostatectomy: feasibility and utility. Int Braz J Urol. 2007 May-Jun 2007;33(3):347-52; discussion 352-4. doi:10.1590/s1677-55382007000300007

547. Nakamura LY, Nunez RN, Castle EP, Andrews PE, Humphreys MR. Different approaches to an inguinal hernia repair during a simultaneous robot-assisted radical prostatectomy. J Endourol. Apr 2011;25(4):621-4. doi:10.1089/end.2010.0417

548. Namiki S, Egawa S, Baba S, et al. Recovery of quality of life in year after laparoscopic or retropubic radical prostatectomy: a multi-institutional longitudinal study. Urology. Mar 2005;65(3):517-23. doi:10.1016/j.urology.2004.09.065

549. Nandipati KC, Raina R, Agarwal A, Zippe CD. Nerve-sparing surgery significantly affects long-term continence after radical prostatectomy. Urology. Dec 2007;70(6):1127-30. doi:10.1016/j.urology.2007.07.042

550. Neff DA, See WA. Laparoscopic mesh herniorrhaphy: impact on outcomes associated with radical retropubic prostatectomy. Urol Oncol. 2011 Jan-Feb 2011;29(1):66-9. doi:10.1016/j.urolonc.2009.06.006

551. Neill MG, Chabert CC, Merrilees DA, Eden CG. The impact of training on service provision in laparoscopic radical prostatectomy. BJU Int. May 2009;103(9):1231-4; discussion 1234-5. doi:10.1111/j.1464-410X.2008.08262.x

552. Neill MG, Lockwood GA, McCluskey SA, Fleshner NE. Preoperative evaluation of the "hostile pelvis" in radical prostatectomy with computed tomographic pelvimetry. BJU Int. Mar 2007;99(3):534-8. doi:10.1111/j.1464-410X.2006.06640.x

553. Neill MG, Louie-Johnsun M, Chabert C, Eden C. Does intrafascial dissection during nerve-sparing laparoscopic radical prostatectomy compromise cancer control? BJU Int. Dec 2009;104(11):1730-3.

554. Nelson B, Kaufman M, Broughton G, et al. Comparison of length of hospital stay between radical retropubic prostatectomy and robotic assisted laparoscopic prostatectomy. J Urol. Mar 2007;177(3):929-31. doi:10.1016/j.juro.2006.10.070

555. Nguyen MM, Kamoi K, Stein RJ, et al. Early continence outcomes of posterior musculofascial plate reconstruction during robotic and laparoscopic prostatectomy. BJU Int. May 2008;101(9):1135-9. doi:10.1111/j.1464-410X.2007.07425.x

556. Nielsen ME, Schaeffer EM, Marschke P, Walsh PC. High anterior release of the levator fascia improves sexual function following open radical retropubic prostatectomy. J Urol. Dec 2008;180(6):2557-64; discussion 2564. doi:10.1016/j.juro.2008.08.047

557. Nilsson AE, Carlsson S, Jonsson NM, Onelöv E, Steineck G, Wiklund NP. Erectile function after robotic nerve sparing and semi-sparing of the neurovascular bundles. J Robot Surg. 2007;1(3):191-5. doi:10.1007/s11701-007-0034-x

558. Noh C, Kshirsagar A, Mohler JL. Outcomes after radical retropubic prostatectomy. Urology. Feb 2003;61(2):412-6. doi:10.1016/s0090-4295(02)02147-7

559. Noldus J, Michl U, Graefen M, Haese A, Hammerer P, Huland H. Patient-reported sexual function after nerve-sparing radical retropubic prostatectomy. Eur Urol. Aug 2002;42(2):118-24. doi:10.1016/s0302-2838(02)00219-1

560. Novara G, Ficarra V, D'Elia C, Secco S, Cavalleri S, Artibani W. Prospective evaluation with standardised criteria for postoperative complications after robotic-assisted laparoscopic radical prostatectomy. Eur Urol. Mar 2010;57(3):363-70. doi:10.1016/j.eururo.2009.11.032

561. Novara G, Ficarra V, D'Elia C, Secco S, Cavalleri S, Artibani W. Trifecta outcomes after robot-assisted laparoscopic radical prostatectomy. BJU Int. Jan 2011;107(1):100-4. doi:10.1111/j.1464-410X.2010.09505.x

562. Novara G, Ficarra V, D'elia C, et al. Evaluating urinary continence and preoperative predictors of urinary continence after robot assisted laparoscopic radical prostatectomy. J Urol. Sep 2010;184(3):1028-33. doi:10.1016/j.juro.2010.04.069

563. Novara G, Ficarra V, D'Elia C, et al. Preoperative criteria to select patients for bilateral nerve-sparing robotic-assisted radical prostatectomy. J Sex Med. Feb 2010;7(2 Pt 1):839-45. doi:10.1111/j.1743-6109.2009.01589.x

564. Nowfar S, Kopp R, Palazzi-Churas K, Derweesh IH, Kane CJ. Initial experience with aspirin use during robotic radical prostatectomy. J Laparoendosc Adv Surg Tech A. Apr 2012;22(3):225-9. doi:10.1089/lap.2011.0388

565. O'Brien BA, Cohen RJ, Wheeler TM, Moorin RE. A post-radical-prostatectomy nomogram incorporating new pathological variables and interaction terms for improved prognosis. BJU Int. Feb 2011;107(3):389-95. doi:10.1111/j.1464-410X.2010.09539.x

566. O'Donnell PD, Finan BF. Continence following nerve-sparing radical prostatectomy. J Urol. Nov 1989;142(5):1227-8; discussion 1229. doi:10.1016/s0022-5347(17)39038-9

567. O'Malley PJ, Van Appledorn S, Bouchier-Hayes DM, Crowe H, Costello AJ. Robotic radical prostatectomy in Australia: initial experience. World J Urol. Jun 2006;24(2):165-70. doi:10.1007/s00345-006-0064-4

568. O'Malley RL, Telegrafi S, Laze J, Lepor H. Para-anastomotic haematoma volume predicts the presence of anastomotic extravasation after radical retropubic prostatectomy. BJU Int. Jan 2010;105(1):34-6. doi:10.1111/j.1464-410X.2009.08709.x

569. Okamura T, Akita H, Tozawa K, Kohri K. Clinicopathological evaluation of radical retropubic prostatectomy-positive outcome even at institutes with relatively limited experience. Hinyokika Kiyo. Aug 2007;53(8):533-6; discussion 537.

570. Olsson LE, Salomon L, Nadu A, et al. Prospective patient-reported continence after laparoscopic radical prostatectomy. Urology. Oct 2001;58(4):570-2.

571. Ong WL, Evans SM, Spelman T, Kearns PA, Murphy DG, Millar JL. Comparison of oncological and health-related quality of life outcomes between open and robot-assisted radical prostatectomy for localised prostate cancer - findings from the population-based Victorian Prostate Cancer Registry. BJU Int. Oct 2016;118(4):563-9. doi:10.1111/bju.13380

572. Orvieto MA, Alsikafi NF, Shalhav AL, et al. Impact of surgical margin status on long-term cancer control after radical prostatectomy. BJU Int. Dec 2006;98(6):1199-203. doi:10.1111/j.1464-410X.2006.06563.x

573. Orvieto MA, Coelho RF, Chauhan S, Palmer KJ, Rocco B, Patel VR. Incidence of lymphoceles after robot-assisted pelvic lymph node dissection. BJU Int. Oct 2011;108(7):1185-90. doi:10.1111/j.1464-410X.2011.10094.x

574. Ou YC, Yang CK, Chang KS, et al. The surgical learning curve for robotic-assisted laparoscopic radical prostatectomy: experience of a single surgeon with 500 cases in Taiwan, China. Asian J Androl. 2014 Sep-Oct 2014;16(5):728-34. doi:10.4103/1008-682X.128515

575. Ou YC, Yang CR, Wang J, Cheng CL, Patel VR. Comparison of robotic-assisted versus retropubic radical prostatectomy performed by a single surgeon. Anticancer Res. May 2009;29(5):1637-42.

576. Ou YC, Yang CR, Wang J, Cheng CL, Patel VR. Robotic-assisted laparoscopic radical prostatectomy: learning curve of first 100 cases. Int J Urol. Jul 2010;17(7):635-40. doi:10.1111/j.1442-2042.2010.02546.x

577. Ou YC, Yang CR, Wang J, et al. The learning curve for reducing complications of robotic-assisted laparoscopic radical prostatectomy by a single surgeon. BJU Int. Aug 2011;108(3):420-5. doi:10.1111/j.1464-410X.2010.09847.x

578. Ozu C, Hagiuda J, Nakagami Y, et al. Radical retropubic prostatectomy with running vesicourethral anastomosis and early catheter removal: our experience. Int J Urol. May 2009;16(5):487-92. doi:10.1111/j.1442-2042.2009.02281.x

579. Paiva CS, Andreoni C, Cunha GP, Khalil W, Ortiz V. Differences among patients undergoing perineal or retropubic radical prostatectomy in pain and perioperative variables: a prospective study. BJU Int. Nov 2009;104(9):1219-26. doi:10.1111/j.1464-410X.2009.08551.x

580. Palisaar JR, Wenske S, Sommerer F, Hinkel A, Noldus J. Open radical retropubic prostatectomy gives favourable surgical and functional outcomes after transurethral resection of the prostate. BJU Int. Sep 2009;104(5):611-5. doi:10.1111/j.1464-410X.2009.08474.x

581. Papachristos A, Basto M, Te Marvelde L, Moon D. Laparoscopic versus robotic-assisted radical prostatectomy: an Australian single-surgeon series. ANZ J Surg. Mar 2015;85(3):154-8. doi:10.1111/ans.12602

582. Parikh A, Toepfer N, Baylor K, Henry Y, Berger P, Rukstalis D. Preoperative aspirin is safe in patients undergoing urologic robot-assisted surgery. J Endourol. Jul 2012;26(7):852-6. doi:10.1089/end.2011.0491

583. Park B, Kim W, Jeong BC, et al. Comparison of oncological and functional outcomes of pure versus robotic-assisted laparoscopic radical prostatectomy performed by a single surgeon. Scand J Urol. Feb 2013;47(1):10-8. doi:10.3109/00365599.2012.696137

584. Park EY, Koo BN, Min KT, Nam SH. The effect of pneumoperitoneum in the steep Trendelenburg position on cerebral oxygenation. Acta Anaesthesiol Scand. Aug 2009;53(7):895-9. doi:10.1111/j.1399-6576.2009.01991.x

585. Park JW, Won Lee H, Kim W, et al. Comparative assessment of a single surgeon's series of laparoscopic radical prostatectomy: conventional versus robot-assisted. J Endourol. Apr 2011;25(4):597-602. doi:10.1089/end.2010.0229

586. Park SY, Jeong W, Choi YD, Chung BH, Hong SJ, Rha KH. Yonsei experience in robotic urologic surgery-application in various urological procedures. Yonsei Med J. Dec 2008;49(6):897-900. doi:10.3349/ymj.2008.49.6.897

587. Park S, Jaffer O, Lotan Y, Saboorian H, Roehrborn CG, Cadeddu JA. Contemporary laparoscopic and open radical retropubic prostatectomy: pathologic outcomes and Kattan postoperative nomograms are equivalent. Urology. Jan 2007;69(1):118-22. doi:10.1016/j.urology.2006.09.033

588. Park SY, Ham WS, Choi YD, Rha KH. Robot-assisted Laparoscopic Radical Prostatectomy: Clinical Experience of 200 Cases. Korean J Urol. 3/ 2008;49(3):215-220.

589. Parker WR, Wang R, He C, Wood DP. Five year expanded prostate cancer index composite-based quality of life outcomes after prostatectomy for localized prostate cancer. BJU Int. Feb 2011;107(4):585-90. doi:10.1111/j.1464-410X.2010.09579.x

590. Pasticier G, Rietbergen JB, Guillonneau B, Fromont G, Menon M, Vallancien G. Robotically assisted laparoscopic radical prostatectomy: feasibility study in men. Eur Urol. Jul 2001;40(1):70-4. doi:10.1159/000049751

591. Pastore AL, Palleschi G, Messas A, et al. Are early continence recovery and oncologic outcomes influenced by use of different devices in prostatic apex dissection during laparoscopic radical prostatectomy? J Endourol. Nov 2014;28(11):1313-9. doi:10.1089/end.2014.0255

592. Pastore AL, Palleschi G, Silvestri L, et al. Laparoscopic radical prostatectomy after previous transurethral resection of prostate using a catheter balloon inflated in prostatic urethra: Oncological and functional outcomes from a matched pair analysis. Int J Urol. Nov 2015;22(11):1037-42. doi:10.1111/iju.12869

593. Patel VR, Coelho RF, Chauhan S, et al. Continence, potency and oncological outcomes after robotic-assisted radical prostatectomy: early trifecta results of a high-volume surgeon. BJU Int. Sep 2010;106(5):696-702. doi:10.1111/j.1464-410X.2010.09541.x

594. Patel VR, Coelho RF, Palmer KJ, Rocco B. Periurethral suspension stitch during robot-assisted laparoscopic radical prostatectomy: description of the technique and continence outcomes. Eur Urol. Sep 2009;56(3):472-8. doi:10.1016/j.eururo.2009.06.007

595. Patel VR, Coelho RF, Rocco B, et al. Positive surgical margins after robotic assisted radical prostatectomy: a multi-institutional study. J Urol. Aug 2011;186(2):511-6. doi:10.1016/j.juro.2011.03.112

596. Patel VR, Palmer KJ, Coughlin G, Samavedi S. Robot-assisted laparoscopic radical prostatectomy: perioperative outcomes of 1500 cases. J Endourol. Oct 2008;22(10):2299-305. doi:10.1089/end.2008.9711

597. Patel VR, Shah S, Arend D. Histopathologic outcomes of robotic radical prostatectomy. ScientificWorldJournal. Jun 2006;6:2566-72. doi:10.1100/tsw.2006.397

598. Patel VR, Sivaraman A, Coelho RF, et al. Pentafecta: a new concept for reporting outcomes of robot-assisted laparoscopic radical prostatectomy. Eur Urol. May 2011;59(5):702-7. doi:10.1016/j.eururo.2011.01.032

599. Patel VR, Thaly R, Shah K. Robotic radical prostatectomy: outcomes of 500 cases. BJU Int. May 2007;99(5):1109-12. doi:10.1111/j.1464-410X.2007.06762.x

600. Patel VR, Tully AS, Holmes R, Lindsay J. Robotic radical prostatectomy in the community setting--the learning curve and beyond: initial 200 cases. J Urol. Jul 2005;174(1):269-72. doi:10.1097/01.ju.0000162082.12962.40

601. Paterson C, Alashkham A, Lang S, Nabi G. Early oncological and functional outcomes following radical treatment of high-risk prostate cancer in men older than 70 years: A prospective longitudinal study. Urol Oncol. Aug 2016;34(8):335.e1-7. doi:10.1016/j.urolonc.2016.03.002

602. Paterson C, McLuckie S, Yew-Fung C, Tang B, Lang S, Nabi G. Videotaping of surgical procedures and outcomes following extraperitoneal laparoscopic radical prostatectomy for clinically localized prostate cancer. J Surg Oncol. Dec 2016;114(8):1016-1023. doi:10.1002/jso.24484

603. Patil N, Krane L, Javed K, Williams T, Bhandari M, Menon M. Evaluating and grading cystographic leakage: correlation with clinical outcomes in patients undergoing robotic prostatectomy. BJU Int. Apr 2009;103(8):1108-10. doi:10.1111/j.1464-410X.2008.08168.x

604. Paul A, Ploussard G, Nicolaiew N, et al. Oncologic outcome after extraperitoneal laparoscopic radical prostatectomy: midterm follow-up of 1115 procedures. Eur Urol. Feb 2010;57(2):267-72. doi:10.1016/j.eururo.2009.09.029

605. Pavlovich CP, Trock BJ, Sulman A, Wagner AA, Mettee LZ, Su LM. 3-year actuarial biochemical recurrence-free survival following laparoscopic radical prostatectomy: experience from a tertiary referral center in the United States. J Urol. Mar 2008;179(3):917-21; discussion 921-2. doi:10.1016/j.juro.2007.10.067

606. Pearce SM, Pariser JJ, Karrison T, Patel SG, Eggener SE. Comparison of Perioperative and Early Oncologic Outcomes between Open and Robotic Assisted Laparoscopic Prostatectomy in a Contemporary Population Based Cohort. J Urol. 07 2016;196(1):76-81. doi:10.1016/j.juro.2016.01.105

607. Peeters E, Joniau S, Van Poppel H, Miserez M. Case-matched analysis of outcome after open retropubic radical prostatectomy in patients with previous preperitoneal inguinal hernia repair. Br J Surg. Mar 2012;99(3):431-5. doi:10.1002/bjs.7832

608. Pepe P, Fraggetta F, Galia A, Candiano G, Grasso G, Aragona F. Is a single focus of low-grade prostate cancer diagnosed on saturation biopsy predictive of clinically insignificant cancer? Urol Int. 2010;84(4):440-4. doi:10.1159/000296293

609. Perer E, Lee DI, Ahlering T, Clayman RV. Robotic revelation: laparoscopic radical prostatectomy by a nonlaparoscopic surgeon. J Am Coll Surg. Oct 2003;197(4):693-6. doi:10.1016/S1072-7515(03)00723-3

610. Permpongkosol S, Link RE, Su LM, et al. Complications of 2,775 urological laparoscopic procedures: 1993 to 2005. J Urol. Feb 2007;177(2):580-5. doi:10.1016/j.juro.2006.09.031

611. Pettus JA, Weight CJ, Thompson CJ, Middleton RG, Stephenson RA. Biochemical failure in men following radical retropubic prostatectomy: impact of surgical margin status and location. J Urol. Jul 2004;172(1):129-32. doi:10.1097/01.ju.0000132160.68779.96

612. Philippou P, Waine E, Rowe E. Robot-assisted laparoscopic prostatectomy versus open: comparison of the learning curve of a single surgeon. J Endourol. Aug 2012;26(8):1002-8. doi:10.1089/end.2011.0569

613. Phinthusophon K, Nualyong C, Srinualnad S, Taweemonkongsap T, Amornvesukij T. Laparoscopic radical prostatectomy: transperitoneal laparoscopic radical prostatectomy versus extraperitoneal endoscopic radical prostatectomy. J Med Assoc Thai. Dec 2007;90(12):2644-50.

614. Pick DL, Osann K, Skarecky D, Narula N, Finley DS, Ahlering TE. The impact of cavernosal nerve preservation on continence after robotic radical prostatectomy. BJU Int. Nov 2011;108(9):1492-6. doi:10.1111/j.1464-410X.2010.10015.x

615. Pierorazio PM, Mullins JK, Eifler JB, et al. Contemporaneous comparison of open vs minimally-invasive radical prostatectomy for high-risk prostate cancer. BJU Int. Oct 2013;112(6):751-7. doi:10.1111/j.1464-410X.2012.11757.x

616. Ploussard G, de la Taille A, Moulin M, et al. Comparisons of the perioperative, functional, and oncologic outcomes after robot-assisted versus pure extraperitoneal laparoscopic radical prostatectomy. Eur Urol. Mar 2014;65(3):610-9. doi:10.1016/j.eururo.2012.11.049

617. Ploussard G, Salomon L, Allory Y, et al. Pathological findings and prostate-specific antigen outcomes after laparoscopic radical prostatectomy for high-risk prostate cancer. BJU Int. Jul 2010;106(1):86-90. doi:10.1111/j.1464-410X.2009.09080.x

618. Ploussard G, Xylinas E, Paul A, et al. Is robot assistance affecting operating room time compared with pure retroperitoneal laparoscopic radical prostatectomy? J Endourol. Jun 2009;23(6):939-43. doi:10.1089/end.2008.0521

619. Ploussard G, Xylinas E, Salomon L, et al. Robot-assisted extraperitoneal laparoscopic radical prostatectomy: experience in a high-volume laparoscopy reference centre. BJU Int. Apr 2010;105(8):1155-60. doi:10.1111/j.1464-410X.2009.09013.x

620. Polland AR, Graversen JA, Mues AC, Badani KK. Polyglyconate unidirectional barbed suture for posterior reconstruction and anastomosis during robot-assisted prostatectomy: effect on procedure time, efficacy, and minimum 6-month follow-up. J Endourol. Sep 2011;25(9):1493-6. doi:10.1089/end.2010.0668

621. Porpiglia F, Checcucci E, Amparore D, et al. Augmented-reality robot-assisted radical prostatectomy using hyper-accuracy three-dimensional reconstruction (HA3D™) technology: a radiological and pathological study. BJU Int. 05 2019;123(5):834-845. doi:10.1111/bju.14549

622. Porpiglia F, Fiori C, Bertolo R, et al. Five-year Outcomes for a Prospective Randomised Controlled Trial Comparing Laparoscopic and Robot-assisted Radical Prostatectomy. Eur Urol Focus. Nov 2016;doi:10.1016/j.euf.2016.11.007

623. Porpiglia F, Fiori C, Grande S, Morra I, Scarpa RM. Selective versus standard ligature of the deep venous complex during laparoscopic radical prostatectomy: effects on continence, blood loss, and margin status. Eur Urol. Jun 2009;55(6):1377-83. doi:10.1016/j.eururo.2009.02.009

624. Porpiglia F, Morra I, Lucci Chiarissi M, et al. Randomised controlled trial comparing laparoscopic and robot-assisted radical prostatectomy. Eur Urol. Apr 2013;63(4):606-14. doi:10.1016/j.eururo.2012.07.007

625. Porpiglia F, Terrone C, Tarabuzzi R, et al. Transperitoneal versus extraperitoneal laparoscopic radical prostatectomy: experience of a single center. Urology. Aug 2006;68(2):376-80. doi:10.1016/j.urology.2006.02.039

626. Potdevin L, Ercolani M, Jeong J, Kim IY. Functional and oncologic outcomes comparing interfascial and intrafascial nerve sparing in robot-assisted laparoscopic radical prostatectomies. J Endourol. Sep 2009;23(9):1479-84. doi:10.1089/end.2009.0369

627. Poulakis V, de Vries R, Dillenburg W, Altmansberger HM, Becht E. Laparoscopic radical prostatectomy: impact of modified apical and posterolateral dissection in reduction of positive surgical margins in patients with clinical stage T2 prostate cancer and high risk of extracapsular extension. J Endourol. May 2006;20(5):332-9. doi:10.1089/end.2006.20.332

628. Poulakis V, Ferakis N, Dillenburg W, Vries R, Witzsch U, Becht E. Laparoscopic radical prostatectomy using an extraperitoneal approach: Nordwest hospital technique and initial experience in 255 cases. J Endourol. Jan 2006;20(1):45-53. doi:10.1089/end.2006.20.45

629. Poulakis V, Skriapas K, de Vries R, Dillenburg W, Witzsch U, Becht E. Vesicourethral anastomosis during endoscopic extraperitoneal radical prostatectomy: a prospective comparison between the single-knot running and interrupted technique. Urology. Dec 2006;68(6):1284-9. doi:10.1016/j.urology.2006.08.1063

630. Poulakis V, Witzsch U, de Vries R, Dillenburg W, Becht E. Laparoscopic radical prostatectomy in men older than 70 years of age with localized prostate cancer: comparison of morbidity, reconvalescence, and short-term clinical outcomes between younger and older men. Eur Urol. May 2007;51(5):1341-8; discussion 1349. doi:10.1016/j.eururo.2006.12.013

631. Prasad SM, Gu X, Lavelle R, Lipsitz SR, Hu JC. Comparative effectiveness of perineal versus retropubic and minimally invasive radical prostatectomy. J Urol. Jan 2011;185(1):111-5. doi:10.1016/j.juro.2010.08.090

632. Prasad SM, Keating NL, Wang Q, et al. Variations in surgeon volume and use of pelvic lymph node dissection with open and minimally invasive radical prostatectomy. Urology. Sep 2008;72(3):647-52; discussion 652-3. doi:10.1016/j.urology.2008.03.067

633. Prasad SM, Large MC, Patel AR, et al. Early removal of urethral catheter with suprapubic tube drainage versus urethral catheter drainage alone after robot-assisted laparoscopic radical prostatectomy. J Urol. Jul 2014;192(1):89-95. doi:10.1016/j.juro.2014.01.004

634. Prewitt R, Bochkarev V, McBride CL, Kinney S, Oleynikov D. The patterns and costs of the Da Vinci robotic surgery system in a large academic institution. J Robot Surg. May 2008;2(1):17-20. doi:10.1007/s11701-008-0075-9

635. Pu XY, Wang XH, Wu YL, Wang HP. Comparative study of the impact of 3- versus 8-month neoadjuvant hormonal therapy on outcome of laparoscopic radical prostatectomy. J Cancer Res Clin Oncol. Aug 2007;133(8):555-62. doi:10.1007/s00432-007-0204-2

636. Punnen S, Meng MV, Cooperberg MR, Greene KL, Cowan JE, Carroll PR. How does robot-assisted radical prostatectomy (RARP) compare with open surgery in men with high-risk prostate cancer? BJU Int. Aug 2013;112(4):E314-20. doi:10.1111/j.1464-410X.2012.11493.x

637. Quattrone C, Cicione A, Oliveira C, et al. Retropubic, laparoscopic and mini-laparoscopic radical prostatectomy: a prospective assessment of patient scar satisfaction. World J Urol. Aug 2015;33(8):1181-7. doi:10.1007/s00345-014-1425-z

638. Quinn DI, Henshall SM, Brenner PC, et al. Prognostic significance of preoperative factors in localized prostate carcinoma treated with radical prostatectomy: importance of percentage of biopsies that contain tumor and the presence of biopsy perineural invasion. Cancer. Apr 2003;97(8):1884-93. doi:10.1002/cncr.11263

639. Quinn DI, Henshall SM, Haynes AM, et al. Prognostic significance of pathologic features in localized prostate cancer treated with radical prostatectomy: implications for staging systems and predictive models. J Clin Oncol. Aug 2001;19(16):3692-705. doi:10.1200/JCO.2001.19.16.3692

640. Rabbani F, Ramasamy R, Patel MI, et al. Predictors of recovery of erectile function after unilateral cavernous nerve graft reconstruction at radical retropubic prostatectomy. J Sex Med. Jan 2010;7(1 Pt 1):166-81. doi:10.1111/j.1743-6109.2009.01436.x

641. Rabbani F, Stapleton AM, Kattan MW, Wheeler TM, Scardino PT. Factors predicting recovery of erections after radical prostatectomy. J Urol. Dec 2000;164(6):1929-34.

642. Rabbani F, Yunis LH, Pinochet R, et al. Comprehensive standardized report of complications of retropubic and laparoscopic radical prostatectomy. Eur Urol. Mar 2010;57(3):371-86. doi:10.1016/j.eururo.2009.11.034

643. Rajih E, Meskawi M, Alenizi AM, et al. Perioperative predictors for post-prostatectomy urinary incontinence in prostate cancer patients following robotic-assisted radical prostatectomy: Long-term results of a Canadian prospective cohort. Can Urol Assoc J. May 2019;13(5):E125-e131. doi:10.5489/cuaj.5356

644. Ramon J, Leandri P, Rossignol G, Gautier JR. Urinary continence following radical retropubic prostatectomy. Br J Urol. Jan 1993;71(1):47-51. doi:10.1111/j.1464-410x.1993.tb15879.x

645. Ramsden AR, Chodak GW. Can leakage at the vesico-urethral anastomosis be predicted after radical retropubic prostatectomy? BJU Int. Mar 2004;93(4):503-6. doi:10.1111/j.1464-410x.2003.04668.x

646. Rasiah KK, Stricker PD, Haynes AM, et al. Prognostic significance of Gleason pattern in patients with Gleason score 7 prostate carcinoma. Cancer. Dec 2003;98(12):2560-5. doi:10.1002/cncr.11850

647. Rassweiler J, Binder J, Frede T. Robotic and telesurgery: will they change our future? Curr Opin Urol. May 2001;11(3):309-20. doi:10.1097/00042307-200105000-00012

648. Rassweiler J, Frede T, Seemann O, Stock C, Sentker L. Telesurgical laparoscopic radical prostatectomy. Initial experience. Eur Urol. Jul 2001;40(1):75-83. doi:10.1159/000049752

649. Rassweiler J, Schulze M, Teber D, et al. Laparoscopic radical prostatectomy with the Heilbronn technique: oncological results in the first 500 patients. J Urol. Mar 2005;173(3):761-4. doi:10.1097/01.ju.0000153486.94741.e5

650. Rassweiler J, Schulze M, Teber D, Seemann O, Frede T. Laparoscopic radical prostatectomy: functional and oncological outcomes. Curr Opin Urol. Mar 2004;14(2):75-82.

651. Rassweiler J, Seemann O, Hatzinger M, Schulze M, Frede T. Technical evolution of laparoscopic radical prostatectomy after 450 cases. J Endourol. Apr 2003;17(3):143-54. doi:10.1089/089277903321618707

652. Rassweiler J, Seemann O, Schulze M, Teber D, Hatzinger M, Frede T. Laparoscopic versus open radical prostatectomy: a comparative study at a single institution. J Urol. May 2003;169(5):1689-93. doi:10.1097/01.ju.0000062614.56629.41

653. Rassweiler J, Sentker L, Seemann O, Hatzinger M, Rumpelt HJ. Laparoscopic radical prostatectomy with the Heilbronn technique: an analysis of the first 180 cases. J Urol. Dec 2001;166(6):2101-8.

654. Rassweiler J, Stolzenburg J, Sulser T, et al. Laparoscopic radical prostatectomy--the experience of the German Laparoscopic Working Group. Eur Urol. Jan 2006;49(1):113-9. doi:10.1016/j.eururo.2005.10.003

655. Raventos Busquets C, Gomez Lanza E, Cecchini Rossell L, Trilla Herrera E, Orsola los de Santos A, Planas Morin J. Comparison between open and laparoscopic approach in radical prostatectomy. Actas Urol Esp. 2007;31(2):141-5.

656. Razi A, Yahyazadeh SR, Sedighi Gilani MA, Kazemeyni SM. Bladder neck preservation during radical retropubic prostatectomy and postoperative urinary continence. Urol J. 2009;6(1):23-6; discussion 26.

657. Rehman J, Ragab MM, Venkatesh R, et al. Laparoscopic radical prostatectomy: Washington University initial experience and prospective evaluation of quality of life. J Endourol. Apr 2004;18(3):277-87. doi:10.1089/089277904773582903

658. Remzi M, Klingler HC, Tinzl MV, et al. Morbidity of laparoscopic extraperitoneal versus transperitoneal radical prostatectomy verus open retropubic radical prostatectomy. Eur Urol. Jul 2005;48(1):83-9; discussion 89. doi:10.1016/j.eururo.2005.03.026

659. Ritch CR, You C, May AT, et al. Biochemical recurrence-free survival after robotic-assisted laparoscopic vs open radical prostatectomy for intermediate- and high-risk prostate cancer. Urology. Jun 2014;83(6):1309-15. doi:10.1016/j.urology.2014.02.023

660. Rocco B, Gregori A, Stener S, et al. Posterior reconstruction of the rhabdosphincter allows a rapid recovery of continence after transperitoneal videolaparoscopic radical prostatectomy. Eur Urol. Apr 2007;51(4):996-1003. doi:10.1016/j.eururo.2006.10.014

661. Rocco B, Jereczek-Fossa BA, Matei DV, et al. Intraoperative radiotherapy during radical prostatectomy for intermediate-risk to locally advanced prostate cancer: treatment technique and evaluation of perioperative and functional outcome vs standard radical prostatectomy, in a matched-pair analysis. BJU Int. Dec 2009;104(11):1624-30. doi:10.1111/j.1464-410X.2009.08668.x

662. Rocco B, Matei DV, Melegari S, et al. Robotic vs open prostatectomy in a laparoscopically naive centre: a matched-pair analysis. BJU Int. Oct 2009;104(7):991-5. doi:10.1111/j.1464-410X.2009.08532.x

663. Rocco F, Carmignani L, Acquati P, et al. Restoration of posterior aspect of rhabdosphincter shortens continence time after radical retropubic prostatectomy. J Urol. Jun 2006;175(6):2201-6. doi:10.1016/S0022-5347(06)00262-X

664. Rocco F, Carmignani L, Acquati P, et al. Early continence recovery after open radical prostatectomy with restoration of the posterior aspect of the rhabdosphincter. Eur Urol. Aug 2007;52(2):376-83. doi:10.1016/j.eururo.2007.01.109

665. Rochat CH, Sauvain J, Dubernard P, Hebert AE, Kreaden U. Mid-term biochemical recurrence-free outcomes following robotic versus laparoscopic radical prostatectomy. J Robot Surg. Dec 2011;5(4):251-7. doi:10.1007/s11701-011-0266-7

666. Rodriguez AR, Kapoor R, Pow-Sang JM. Laparoscopic extraperitoneal radical prostatectomy in complex surgical cases. J Urol. May 2007;177(5):1765-70. doi:10.1016/j.juro.2007.01.034

667. Rodriguez AR, Rachna K, Pow-Sang JM. Laparoscopic extraperitoneal radical prostatectomy: impact of the learning curve on perioperative outcomes and margin status. JSLS. 2010 Jan-Mar 2010;14(1):6-13. doi:10.4293/108680809X12589998404209

668. Rodriguez E, Finley DS, Skarecky D, Ahlering TE. Single institution 2-year patient reported validated sexual function outcomes after nerve sparing robot assisted radical prostatectomy. J Urol. Jan 2009;181(1):259-63. doi:10.1016/j.juro.2008.09.015

669. Roehl KA, Han M, Ramos CG, Antenor JA, Catalona WJ. Cancer progression and survival rates following anatomical radical retropubic prostatectomy in 3,478 consecutive patients: long-term results. J Urol. Sep 2004;172(3):910-4. doi:10.1097/01.ju.0000134888.22332.bb

670. Rogers CG, Sammon JD, Sukumar S, Diaz M, Peabody J, Menon M. Robot assisted radical prostatectomy for elderly patients with high risk prostate cancer. Urol Oncol. Feb 2013;31(2):193-7. doi:10.1016/j.urolonc.2010.11.018

671. Rogers CG, Su LM, Link RE, Sullivan W, Wagner A, Pavlovich CP. Age stratified functional outcomes after laparoscopic radical prostatectomy. J Urol. Dec 2006;176(6 Pt 1):2448-52. doi:10.1016/j.juro.2006.07.153

672. Rogers T, Parra-Davila E, Malcher F, et al. Robotic radical prostatectomy with concomitant repair of inguinal hernia: is it safe? J Robot Surg. Jun 2018;12(2):325-330. doi:10.1007/s11701-017-0737-6

673. Roumeguere T, Bollens R, Vanden Bossche M, et al. Radical prostatectomy: a prospective comparison of oncological and functional results between open and laparoscopic approaches. World J Urol. May 2003;20(6):360-6. doi:10.1007/s00345-002-0306-z

674. Rozet F, Arroyo C, Cathelineau X, Barret E, Prapotnich D, Vallancien G. Extraperitoneal standard laparoscopic radical prostatectomy. J Endourol. Sep 2004;18(7):605-9; discussion 609-10. doi:10.1089/end.2004.18.605

675. Rozet F, Galiano M, Cathelineau X, Barret E, Cathala N, Vallancien G. Extraperitoneal laparoscopic radical prostatectomy: a prospective evaluation of 600 cases. J Urol. Sep 2005;174(3):908-11. doi:10.1097/01.ju.0000169260.42845.c9

676. Rozet F, Jaffe J, Braud G, et al. A direct comparison of robotic assisted versus pure laparoscopic radical prostatectomy: a single institution experience. J Urol. Aug 2007;178(2):478-82. doi:10.1016/j.juro.2007.03.111

677. Ruiz L, Salomon L, Hoznek A, et al. Comparison of early oncologic results of laparoscopic radical prostatectomy by extraperitoneal versus transperitoneal approach. Eur Urol. Jul 2004;46(1):50-4; discussion 54-6. doi:10.1016/j.eururo.2004.04.013

678. Ryu J, Kwon T, Kyung YS, et al. Retropubic versus robot-assisted laparoscopic prostatectomy for prostate cancer: a comparative study of postoperative complications. Korean J Urol. Nov 2013;54(11):756-61. doi:10.4111/kju.2013.54.11.756

679. Sacco E, Prayer-Galetti T, Pinto F, et al. Urinary incontinence after radical prostatectomy: incidence by definition, risk factors and temporal trend in a large series with a long-term follow-up. BJU Int. Jun 2006;97(6):1234-41. doi:10.1111/j.1464-410X.2006.06185.x

680. Sachedina N, De Los Santos R, Manoharan M, Soloway MS. Total prostatectomy and lymph node dissection may be done safely without pelvic drainage: an extended experience of over 600 cases. Can J Urol. Aug 2009;16(4):4721-5.

681. Sagalovich D, Calaway A, Srivastava A, Sooriakumaran P, Tewari AK. Assessment of required nodal yield in a high risk cohort undergoing extended pelvic lymphadenectomy in robotic-assisted radical prostatectomy and its impact on functional outcomes. BJU Int. Jan 2013;111(1):85-94. doi:10.1111/j.1464-410X.2012.11351.x

682. Sahabudin RM, Arni T, Ashani N, et al. Development of robotic program: an Asian experience. World J Urol. Jun 2006;24(2):161-4. doi:10.1007/s00345-006-0069-z

683. Saint-Elie DT, Marshall FF. Impact of laparoscopic inguinal hernia repair mesh on open radical retropubic prostatectomy. Urology. Nov 2010;76(5):1078-82. doi:10.1016/j.urology.2010.01.015

684. Saito FJ, Dall'Oglio MF, Ebaid GX, Bruschini H, Chade DC, Srougi M. Learning curve for radical retropubic prostatectomy. Int Braz J Urol. 2011 Jan-Feb 2011;37(1):67-74; discussion 75-8. doi:10.1590/s1677-55382011000100009

685. Saito S, Namiki S, Numahata K, et al. Relevance of postcatheter removal incontinence to postoperative urinary function after radical prostatectomy. Int J Urol. Sep 2006;13(9):1191-6. doi:10.1111/j.1442-2042.2006.01529.x

686. Sakko AJ, Butler MS, Byers S, et al. Immunohistochemical level of unsulfated chondroitin disaccharides in the cancer stroma is an independent predictor of prostate cancer relapse. Cancer Epidemiol Biomarkers Prev. Sep 2008;17(9):2488-97. doi:10.1158/1055-9965.EPI-08-0204

687. Salomon L, Anastasiadis AG, Katz R, et al. Urinary continence and erectile function: a prospective evaluation of functional results after radical laparoscopic prostatectomy. Eur Urol. Oct 2002;42(4):338-43.

688. Salomon L, Anastasiadis AG, Levrel O, et al. Location of positive surgical margins after retropubic, perineal, and laparoscopic radical prostatectomy for organ-confined prostate cancer. Urology. Feb 2003;61(2):386-90.

689. Salomon L, Levrel O, Anastasiadis AG, et al. Outcome and complications of radical prostatectomy in patients with PSA <10 ng/ml: comparison between the retropubic, perineal and laparoscopic approach. Prostate Cancer Prostatic Dis. 2002;5(4):285-90. doi:10.1038/sj.pcan.4500605

690. Salomon L, Levrel O, de la Taille A, et al. Radical prostatectomy by the retropubic, perineal and laparoscopic approach: 12 years of experience in one center. Eur Urol. Aug 2002;42(2):104-10; discussion 110-1.

691. Samadi DB, Muntner P, Nabizada-Pace F, Brajtbord JS, Carlucci J, Lavery HJ. Improvements in robot-assisted prostatectomy: the effect of surgeon experience and technical changes on oncologic and functional outcomes. J Endourol. Jul 2010;24(7):1105-10. doi:10.1089/end.2010.0136

692. Sammon JD, Karakiewicz PI, Sun M, et al. Robot-assisted versus open radical prostatectomy: the differential effect of regionalization, procedure volume and operative approach. J Urol. Apr 2013;189(4):1289-94. doi:10.1016/j.juro.2012.10.028

693. Sammon JD, Muhletaler F, Peabody JO, Diaz-Insua M, Satyanaryana R, Menon M. Long-term functional urinary outcomes comparing single- vs double-layer urethrovesical anastomosis: two-year follow-up of a two-group parallel randomized controlled trial. Urology. Nov 2010;76(5):1102-7. doi:10.1016/j.urology.2010.05.052

694. Sammon JD, Sharma P, Trinh QD, Ghani KR, Sukumar S, Menon M. Predictors of immediate continence following robot-assisted radical prostatectomy. J Endourol. Apr 2013;27(4):442-6. doi:10.1089/end.2012.0312

695. Sammon J, Kim TK, Trinh QD, et al. Anastomosis during robot-assisted radical prostatectomy: randomized controlled trial comparing barbed and standard monofilament suture. Urology. Sep 2011;78(3):572-9. doi:10.1016/j.urology.2011.03.069

696. Sánchez-Ortiz RF, Andrade-Geigel C, López-Huertas H, Cadillo-Chávez R, Soto-Avilés O. Preoperative International Prostate Symptom Score Predictive of Inguinal Hernia in Patients Undergoing Robotic Prostatectomy. J Urol. 06 2016;195(6):1744-7. doi:10.1016/j.juro.2015.11.069

697. Sanchez-Salas R, Prapotnich D, Rozet F, et al. Laparoscopic radical prostatectomy is feasible and effective in 'fit' senior men with localized prostate cancer. BJU Int. Nov 2010;106(10):1530-6. doi:10.1111/j.1464-410X.2010.09295.x

698. Sano T, Nakashima M, Haitani T, Kajita Y, Shichiri Y. Posterior musculofascial plate reconstruction promotes early restoration of continence and prevents severe incontinence in patients undergoing laparoscopic radical prostatectomy. Int J Urol. May 2012;19(5):475-9. doi:10.1111/j.1442-2042.2011.02954.x

699. Sasaki H, Miki J, Kimura T, et al. Lateral view dissection of the prostato-urethral junction to reduce positive apical margin in laparoscopic radical prostatectomy. Int J Urol. Aug 2009;16(8):664-9. doi:10.1111/j.1442-2042.2009.02328.x

700. Sayyid RK, Simpson WG, Lu C, Terris MK, Klaassen Z, Madi R. Retzius-Sparing Robotic-Assisted Laparoscopic Radical Prostatectomy: A Safe Surgical Technique with Superior Continence Outcomes. J Endourol. 12 2017;31(12):1244-1250. doi:10.1089/end.2017.0490

701. Schmeller N, Keller H, Janetschek G. Head-to-head comparison of retropubic, perineal and laparoscopic radical prostatectomy. Int J Urol. May 2007;14(5):402-5. doi:10.1111/j.1442-2042.2006.01727.x

702. Schmitges J, Sun M, Abdollah F, et al. Blood transfusions in radical prostatectomy: a contemporary population-based analysis. Urology. Feb 2012;79(2):332-8. doi:10.1016/j.urology.2011.08.079

703. Schmitges J, Trinh QD, Bianchi M, et al. The effect of annual surgical caseload on the rates of in-hospital pneumonia and other in-hospital outcomes after radical prostatectomy. Int Urol Nephrol. Jun 2012;44(3):799-806. doi:10.1007/s11255-011-0103-y

704. Schroeck FR, de Sousa CA, Kalman RA, et al. Trainees do not negatively impact the institutional learning curve for robotic prostatectomy as characterized by operative time, estimated blood loss, and positive surgical margin rate. Urology. Apr 2008;71(4):597-601. doi:10.1016/j.urology.2007.12.023

705. Schroeck FR, Sun L, Freedland SJ, et al. Comparison of prostate-specific antigen recurrence-free survival in a contemporary cohort of patients undergoing either radical retropubic or robot-assisted laparoscopic radical prostatectomy. BJU Int. Jul 2008;102(1):28-32. doi:10.1111/j.1464-410X.2008.07607.x

706. Sciarra A, Gentile V, De Matteis A, et al. Long-term experience with an anatomical anterograde approach to radical prostatectomy: results in terms of positive margin rate. Urol Int. 2008;80(2):151-6. doi:10.1159/000112605

707. Selli C, De Antoni P, Moro U, Macchiarella A, Giannarini G, Crisci A. Role of bladder neck preservation in urinary continence following radical retropubic prostatectomy. Scand J Urol Nephrol. 2004;38(1):32-7. doi:10.1080/00365590310017280

708. Sengupta S, Blute ML, Bagniewski SM, et al. After radical retropubic prostatectomy 'insignificant' prostate cancer has a risk of progression similar to low-risk 'significant' cancer. BJU Int. Jan 2008;101(2):170-4. doi:10.1111/j.1464-410X.2007.07270.x

709. Serni S, Masieri L, Lapini A, Nesi G, Carini M. A low incidence of positive surgical margins in prostate cancer at high risk of extracapsular extension after a modified anterograde radical prostatectomy. BJU Int. Feb 2004;93(3):279-83. doi:10.1111/j.1464-410x.2004.04602.x

710. Shah A, Okotie OT, Zhao L, Pins MR, Bhalani V, Dalton DP. Pathologic outcomes during the learning curve for robotic-assisted laparoscopic radical prostatectomy. Int Braz J Urol. 2008 Mar-Apr 2008;34(2):159-62; discussion 163. doi:10.1590/s1677-55382008000200005

711. Shalhav AL, Orvieto MA, Chien GW, Mikhail AA, Zagaja GP, Zorn KC. Minimizing knot tying during reconstructive laparoscopic urology. Urology. Sep 2006;68(3):508-13. doi:10.1016/j.urology.2006.03.071

712. Shannon BA, McNeal JE, Cohen RJ. Transition zone carcinoma of the prostate gland: a common indolent tumour type that occasionally manifests aggressive behaviour. Pathology. Dec 2003;35(6):467-71. doi:10.1080/00313020310001619154

713. Sharma NL, Papadopoulos A, Lee D, et al. First 500 cases of robotic-assisted laparoscopic radical prostatectomy from a single UK centre: learning curves of two surgeons. BJU Int. Sep 2011;108(5):739-47. doi:10.1111/j.1464-410X.2010.09941.x

714. Shelfo SW, Obek C, Soloway MS. Update on bladder neck preservation during radical retropubic prostatectomy: impact on pathologic outcome, anastomotic strictures, and continence. Urology. Jan 1998;51(1):73-8. doi:10.1016/s0090-4295(97)00463-9

715. Shigemura K, Yasufuku T, Yamanaka K, et al. Limited hydration may reduce intraoperative blood loss in retropubic radical prostatectomy. Kobe J Med Sci. Aug 2010;56(1):E18-23.

716. Shikanov SA, Thong A, Gofrit ON, et al. Robotic laparoscopic radical prostatectomy for biopsy Gleason 8 to 10: prediction of favorable pathologic outcome with preoperative parameters. J Endourol. Jul 2008;22(7):1477-81. doi:10.1089/end.2008.0091

717. Shikanov SA, Zorn KC, Zagaja GP, Shalhav AL. Trifecta outcomes after robotic-assisted laparoscopic prostatectomy. Urology. Sep 2009;74(3):619-23. doi:10.1016/j.urology.2009.02.082

718. Shikanov S, Desai V, Razmaria A, Zagaja GP, Shalhav AL. Robotic radical prostatectomy for elderly patients: probability of achieving continence and potency 1 year after surgery. J Urol. May 2010;183(5):1803-7. doi:10.1016/j.juro.2010.01.016

719. Shikanov S, Song J, Royce C, et al. Length of positive surgical margin after radical prostatectomy as a predictor of biochemical recurrence. J Urol. Jul 2009;182(1):139-44. doi:10.1016/j.juro.2009.02.139

720. Shikanov S, Woo J, Al-Ahmadie H, et al. Extrafascial versus interfascial nerve-sparing technique for robotic-assisted laparoscopic prostatectomy: comparison of functional outcomes and positive surgical margins characteristics. Urology. Sep 2009;74(3):611-6. doi:10.1016/j.urology.2009.01.092

721. Shimbo M, Endo F, Matsushita K, et al. Incidence, Risk Factors and a Novel Prevention Technique for Inguinal Hernia after Robot-Assisted Radical Prostatectomy. Urol Int. 2017;98(1):54-60. doi:10.1159/000448339

722. Si-Tu J, Lu MH, Li LY, et al. Prospective evaluation of pentafecta outcomes at 5 years after laparoscopic radical prostatectomy: results of 170 patients at a single center. Neoplasma. 2013;60(3):309-14. doi:10.4149/neo_2013_041

723. Siddiqui SA, Krane LS, Bhandari A, et al. The impact of previous inguinal or abdominal surgery on outcomes after robotic radical prostatectomy. Urology. May 2010;75(5):1079-82. doi:10.1016/j.urology.2009.09.004

724. Silberstein JL, Parsons JK, Palazzi-Churas K, et al. Robot-assisted laparoscopic radical prostatectomy in men with human immunodeficiency virus. Prostate Cancer Prostatic Dis. Dec 2010;13(4):328-32. doi:10.1038/pcan.2010.35

725. Silberstein JL, Su D, Glickman L, et al. A case-mix-adjusted comparison of early oncological outcomes of open and robotic prostatectomy performed by experienced high volume surgeons. BJU Int. Feb 2013;111(2):206-12. doi:10.1111/j.1464-410X.2012.11638.x

726. Silberstein JL, Vickers AJ, Power NE, et al. Pelvic lymph node dissection for patients with elevated risk of lymph node invasion during radical prostatectomy: comparison of open, laparoscopic and robot-assisted procedures. J Endourol. Jun 2012;26(6):748-53. doi:10.1089/end.2011.0266

727. Silva E, Ferreira U, Silva GD, et al. Surgical margins in radical prostatectomy: a comparison between retropubic and laparoscopic surgery. Int Urol Nephrol. 2007;39(3):865-9. doi:10.1007/s11255-006-9128-z

728. Sim HG, Yip SK, Lau WK, Tan JK, Cheng CW. Early experience with robot-assisted laparoscopic radical prostatectomy. Asian J Surg. Oct 2004;27(4):321-5. doi:10.1016/S1015-9584(09)60060-9

729. Sim HG, Yip SK, Lau WK, Tan YH, Wong MY, Cheng CW. Team-based approach reduces learning curve in robot-assisted laparoscopic radical prostatectomy. Int J Urol. May 2006;13(5):560-4. doi:10.1111/j.1442-2042.2006.01354.x

730. Sim KC, Sung DJ, Han NY, et al. Preoperative CT findings of subclinical hernia can predict for postoperative inguinal hernia following robot-assisted laparoscopic radical prostatectomy. Abdom Radiol (NY). 05 2018;43(5):1231-1236. doi:10.1007/s00261-017-1270-9

731. Simforoosh N, Javaherforooshzadeh A, Aminsharifi A, Tabibi A. Early continence after open and laparoscopic radical prostatectomy with sutureless vesicourethral alignment: an alternative technique, 8 years' experience. Urol J. 2009;6(3):163-9.

732. Simon MA, Kim S, Soloway MS. Prostate specific antigen recurrence rates are low after radical retropubic prostatectomy and positive margins. J Urol. Jan 2006;175(1):140-4; discussion 144-5. doi:10.1016/S0022-5347(05)00050-9

733. Simone G, Papalia R, Ferriero M, Guaglianone S, Gallucci M. Laparoscopic "single knot-single running" suture vesico-urethral anastomosis with posterior musculofascial reconstruction. World J Urol. Oct 2012;30(5):651-7. doi:10.1007/s00345-012-0840-2

734. Singh A, Fagin R, Shah G, Shekarriz B. Impact of prostate size and body mass index on perioperative morbidity after laparoscopic radical prostatectomy. J Urol. Feb 2005;173(2):552-4. doi:10.1097/01.ju.0000150101.95236.35

735. Siqueira TM, Mitre AI, Duarte RJ, et al. Transperitoneal versus extraperitoneal laparoscopic radical prostatectomy during the learning curve: does the surgical approach affect the complication rate? Int Braz J Urol. 2010 Jul-Aug 2010;36(4):450-7.

736. Skolarus TA, Hedgepeth RC, Zhang Y, et al. Does robotic technology mitigate the challenges of large prostate size? Urology. Nov 2010;76(5):1117-21. doi:10.1016/j.urology.2010.03.060

737. Slabaugh TK, Marshall FF. A comparison of minimally invasive open and laparoscopic radical retropubic prostatectomy. J Urol. Dec 2004;172(6 Pt 2):2545-8. doi:10.1097/01.ju.0000145059.44004.3b

738. Smith JA, Chan RC, Chang SS, et al. A comparison of the incidence and location of positive surgical margins in robotic assisted laparoscopic radical prostatectomy and open retropubic radical prostatectomy. J Urol. Dec 2007;178(6):2385-9; discussion 2389-90. doi:10.1016/j.juro.2007.08.008

739. Soares R, Di Benedetto A, Dovey Z, Bott S, McGregor RG, Eden CG. Minimum 5-year follow-up of 1138 consecutive laparoscopic radical prostatectomies. BJU Int. Apr 2015;115(4):546-53. doi:10.1111/bju.12887

740. Soderdahl DW, Diaz JI, Rabah DM, Schellhammer PF, Fabrizio MD. Laparoscopic radical prostatectomy: evaluation of specimen pathologic features to critically assess and modify surgical technique. Urology. Sep 2005;66(3):552-6. doi:10.1016/j.urology.2005.03.094

741. Soderdahl DW, Davis JW, Schellhammer PF, et al. Prospective Longitudinal Comparative Study of Health-Related Quality of Life in Patients Undergoing Invasive Treatments for Localized Prostate Cancer. Journal of Endourology. 2005/04/01 2005;19(3):318-326. doi:10.1089/end.2005.19.318

742. Son SJ, Lee SC, Jeong CW, Jeong SJ, Byun SS, Lee SE. Comparison of continence recovery between robot-assisted laparoscopic prostatectomy and open radical retropubic prostatectomy: a single surgeon experience. Korean J Urol. Sep 2013;54(9):598-602. doi:10.4111/kju.2013.54.9.598

743. Son-fa t H, Hio-fai L, Kin L, Men-kin T. Clinical results of radical prostatectomy for patients with prostate cancer in Macau. Chin Med J. 2008;121(4):295-298.

744. Song W, Park JH, Jeon HG, et al. Comparison of Oncologic Outcomes and Complications According to Surgical Approach to Radical Prostatectomy: Special Focus on the Perineal Approach. Clin Genitourin Cancer. 08 2017;15(4):e645-e652. doi:10.1016/j.clgc.2017.01.015

745. Sooriakumaran P, Haendler L, Nyberg T, et al. Biochemical recurrence after robot-assisted radical prostatectomy in a European single-centre cohort with a minimum follow-up time of 5 years. Eur Urol. Nov 2012;62(5):768-74. doi:10.1016/j.eururo.2012.05.024

746. Sooriakumaran P, Pini G, Nyberg T, et al. Erectile Function and Oncologic Outcomes Following Open Retropubic and Robot-assisted Radical Prostatectomy: Results from the LAParoscopic Prostatectomy Robot Open Trial. Eur Urol. 04 2018;73(4):618-627. doi:10.1016/j.eururo.2017.08.015

747. Springer C, Inferrera A, Pini G, Mohammed N, Fornara P, Greco F. Laparoscopic versus open bilateral intrafascial nerve-sparing radical prostatectomy after TUR-P for incidental prostate cancer: surgical outcomes and effect on postoperative urinary continence and sexual potency. World J Urol. Dec 2013;31(6):1505-10. doi:10.1007/s00345-013-1036-0

748. Srinualnad S. Early experience of robotic assisted laparoscopic radical prostatectomy. J Med Assoc Thai. Mar 2008;91(3):377-82.

749. Srinualnad S, Nualyong C. Nerve-sparing laparoscopic radical prostatectomy at Siriraj Hospital. J Med Assoc Thai. Apr 2007;90(4):730-6.

750. Srinualnad S, Nualyong C, Udompunturak S, Kongsuwan W. Endoscopic extraperitoneal radical prostatectomy (EERPE): a new approach for treatment of localized prostate cancer. J Med Assoc Thai. Oct 2006;89(10):1601-8.

751. Srinualnad S, Udompunturak S. Extraperitoneal laparoscopic radical prostatectomy: early experience in Thailand. Asian J Surg. Oct 2007;30(4):272-7. doi:10.1016/S1015-9584(08)60038-X

752. Srivastava A, Chopra S, Pham A, et al. Effect of a risk-stratified grade of nerve-sparing technique on early return of continence after robot-assisted laparoscopic radical prostatectomy. Eur Urol. Mar 2013;63(3):438-44. doi:10.1016/j.eururo.2012.07.009

753. Srougi M, Nesrallah LJ, Kauffmann JR, Nesrallah A, Leite KR. Urinary continence and pathological outcome after bladder neck preservation during radical retropubic prostatectomy: a randomized prospective trial. J Urol. Mar 2001;165(3):815-8.

754. Srougi M, Paranhos M, Leite KM, Dall'Oglio M, Nesrallah L. The influence of bladder neck mucosal eversion and early urinary extravasation on patient outcome after radical retropubic prostatectomy: a prospective controlled trial. BJU Int. Apr 2005;95(6):757-60. doi:10.1111/j.1464-410X.2005.05395.x

755. Stanford JL, Feng Z, Hamilton AS, et al. Urinary and sexual function after radical prostatectomy for clinically localized prostate cancer: the Prostate Cancer Outcomes Study. JAMA. Jan 2000;283(3):354-60. doi:10.1001/jama.283.3.354

756. Stav K, Zacci F, Bahar M, Leibovici D, Lindner A, Zisman A. Intracavernosal saline infusion decreases intraoperative blood loss during radical retropubic prostatectomy. Urol Oncol. 2008 Mar-Apr 2008;26(2):171-4. doi:10.1016/j.urolonc.2007.01.024

757. Sterrett S, Mammen T, Nazemi T, et al. Major urological oncological surgeries can be performed using minimally invasive robotic or laparoscopic methods with similar early perioperative outcomes compared to conventional open methods. World J Urol. Apr 2007;25(2):193-8. doi:10.1007/s00345-006-0140-9

758. Stolzenburg JU, Anderson C, Rabenalt R, Do M, Ho K, Truss MC. Endoscopic extraperitoneal radical prostatectomy in patients with prostate cancer and previous laparoscopic inguinal mesh placement for hernia repair. World J Urol. Sep 2005;23(4):295-9. doi:10.1007/s00345-005-0001-y

759. Stolzenburg JU, Do M, Rabenalt R, et al. Endoscopic extraperitoneal radical prostatectomy: initial experience after 70 procedures. J Urol. Jun 2003;169(6):2066-71. doi:10.1097/01.ju.0000067220.84015.8e

760. Stolzenburg JU, Ho KM, Do M, Rabenalt R, Dorschner W, Truss MC. Impact of previous surgery on endoscopic extraperitoneal radical prostatectomy. Urology. Feb 2005;65(2):325-31. doi:10.1016/j.urology.2004.09.026

761. Stolzenburg JU, Kallidonis P, Do M, et al. A comparison of outcomes for interfascial and intrafascial nerve-sparing radical prostatectomy. Urology. Sep 2010;76(3):743-8. doi:10.1016/j.urology.2010.03.089

762. Stolzenburg JU, Kallidonis P, Hicks J, et al. Effect of bladder neck preservation during endoscopic extraperitoneal radical prostatectomy on urinary continence. Urol Int. 2010;85(2):135-8. doi:10.1159/000314842

763. Stolzenburg JU, Kallidonis P, Minh D, et al. Endoscopic extraperitoneal radical prostatectomy: evolution of the technique and experience with 2400 cases. J Endourol. Sep 2009;23(9):1467-72. doi:10.1089/end.2009.0336

764. Stolzenburg JU, Qazi HA, Holze S, et al. Evaluating the learning curve of experienced laparoscopic surgeons in robot-assisted radical prostatectomy. J Endourol. Jan 2013;27(1):80-5. doi:10.1089/end.2012.0262

765. Stolzenburg JU, Rabenalt R, Dietel A, et al. Hernia repair during endoscopic (laparoscopic) radical prostatectomy. J Laparoendosc Adv Surg Tech A. Feb 2003;13(1):27-31. doi:10.1089/109264203321235430

766. Stolzenburg JU, Rabenalt R, DO M, et al. Endoscopic extraperitoneal radical prostatectomy: oncological and functional results after 700 procedures. J Urol. Oct 2005;174(4 Pt 1):1271-5; discussion 1275.

767. Stolzenburg JU, Rabenalt R, Do M, et al. Endoscopic extraperitoneal radical prostatectomy: the University of Leipzig experience of 1,300 cases. World J Urol. Mar 2007;25(1):45-51. doi:10.1007/s00345-007-0156-9

768. Stranne J, Aus G, Bergdahl S, et al. Post-radical prostatectomy inguinal hernia: a simple surgical intervention can substantially reduce the incidence--results from a prospective randomized trial. J Urol. Sep 2010;184(3):984-9. doi:10.1016/j.juro.2010.04.067

769. Su LM, Link RE, Bhayani SB, Sullivan W, Pavlovich CP. Nerve-sparing laparoscopic radical prostatectomy: replicating the open surgical technique. Urology. Jul 2004;64(1):123-7. doi:10.1016/j.urology.2004.02.010

770. Su YK, Katz BF, Sehgal SS, et al. Does previous transurethral prostate surgery affect oncologic and continence outcomes after RARP? J Robot Surg. Dec 2015;9(4):291-7. doi:10.1007/s11701-015-0529-9

771. Suardi N, Dell'Oglio P, Gallina A, et al. Evaluation of positive surgical margins in patients undergoing robot-assisted and open radical prostatectomy according to preoperative risk groups. Urol Oncol. Feb 2016;34(2):57.e1-7. doi:10.1016/j.urolonc.2015.08.019

772. Suardi N, Ficarra V, Willemsen P, et al. Long-term biochemical recurrence rates after robot-assisted radical prostatectomy: analysis of a single-center series of patients with a minimum follow-up of 5 years. Urology. Jan 2012;79(1):133-8. doi:10.1016/j.urology.2011.08.045

773. Suardi N, Moschini M, Gallina A, et al. Nerve-sparing approach during radical prostatectomy is strongly associated with the rate of postoperative urinary continence recovery. BJU Int. May 2013;111(5):717-22. doi:10.1111/j.1464-410X.2012.11315.x

774. Sugihara T, Yasunaga H, Horiguchi H, et al. Is mechanical bowel preparation in laparoscopic radical prostatectomy beneficial? An analysis of a Japanese national database. BJU Int. Jul 2013;112(2):E76-81. doi:10.1111/j.1464-410X.2012.11725.x

775. Sugihara T, Yasunaga H, Horiguchi H, et al. Robot-assisted versus other types of radical prostatectomy: population-based safety and cost comparison in Japan, 2012-2013. Cancer Sci. Nov 2014;105(11):1421-6. doi:10.1111/cas.12523

776. Sugiono M, Teber D, Anghel G, et al. Assessing the predictive validity and efficacy of a multimodal training programme for laparoscopic radical prostatectomy (LRP). Eur Urol. May 2007;51(5):1332-9; discussion 1340. doi:10.1016/j.eururo.2006.11.029

777. Suh YS, Jang HJ, Song W, et al. Location of positive surgical margin and its association with biochemical recurrence rate do not differ significantly in four different types of radical prostatectomy. Korean J Urol. Dec 2014;55(12):802-7. doi:10.4111/kju.2014.55.12.802

778. Sukkarieh T, Harmon J, Penna F, Parra R. Incidence and management of anastomotic leakage following laparoscopic prostatectomy with implementation of a new anastomotic technique incorporating posterior bladder neck tailoring. J Robot Surg. 2007;1(3):213-5. doi:10.1007/s11701-007-0046-6

779. Sultan MF, Merrilees AD, Chabert CC, Eden CG. Blood loss during laparoscopic radical prostatectomy. J Endourol. Apr 2009;23(4):635-8. doi:10.1089/end.2007.0302

780. Sutherland DE, Linder B, Guzman AM, et al. Posterior rhabdosphincter reconstruction during robotic assisted radical prostatectomy: results from a phase II randomized clinical trial. J Urol. Apr 2011;185(4):1262-7. doi:10.1016/j.juro.2010.11.085

781. Swindle P, Eastham JA, Ohori M, et al. Do margins matter? The prognostic significance of positive surgical margins in radical prostatectomy specimens. J Urol. Sep 2005;174(3):903-7. doi:10.1097/01.ju.0000169475.00949.78

782. Tagawa ST, Dorff TB, Rochanda L, et al. Subclinical haemostatic activation and current surgeon volume predict bleeding with open radical retropubic prostatectomy. BJU Int. Nov 2008;102(9):1086-91. doi:10.1111/j.1464-410X.2008.07780.x

783. Takeda T, Miyajima A, Kaneko G, Hasegawa M, Kikuchi E, Oya M. Unidirectional barbed suture for vesicourethral anastomosis during laparoscopic radical prostatectomy. Asian J Endosc Surg. Aug 2014;7(3):241-5. doi:10.1111/ases.12115

784. Takeyama K, Takahashi S, Maeda T, et al. Comparison of 1-day, 2-day, and 3-day administration of antimicrobial prophylaxis in radical prostatectomy. J Infect Chemother. Oct 2007;13(5):320-3. doi:10.1007/s10156-007-0540-9

785. Talcott JA, Rieker P, Propert KJ, et al. Patient-reported impotence and incontinence after nerve-sparing radical prostatectomy. J Natl Cancer Inst. Aug 1997;89(15):1117-23. doi:10.1093/jnci/89.15.1117

786. Tan G, Srivastava A, Grover S, et al. Optimizing vesicourethral anastomosis healing after robot-assisted laparoscopic radical prostatectomy: lessons learned from three techniques in 1900 patients. J Endourol. Dec 2010;24(12):1975-83. doi:10.1089/end.2009.0630

787. Teber D, Cresswell J, Ates M, et al. Laparoscopic radical prostatectomy in clinical T1a and T1b prostate cancer: oncologic and functional outcomes--a matched-pair analysis. Urology. Mar 2009;73(3):577-81. doi:10.1016/j.urology.2008.09.059

788. Teber D, Erdogru T, Cresswell J, Gözen AS, Frede T, Rassweiler JJ. Analysis of three different vesicourethral anastomotic techniques in laparoscopic radical prostatectomy. World J Urol. Dec 2008;26(6):617-22. doi:10.1007/s00345-008-0281-0

789. Teber D, Erdogru T, Zukosky D, Frede T, Rassweiler J. Prosthetic mesh hernioplasty during laparoscopic radical prostatectomy. Urology. Jun 2005;65(6):1173-8. doi:10.1016/j.urology.2004.12.063

790. Teber D, Gözen AS, Cresswell J, Canda AE, Yencilek F, Rassweiler J. Prevention and management of ureteral injuries occurring during laparoscopic radical prostatectomy: the Heilbronn experience and a review of the literature. World J Urol. Oct 2009;27(5):613-8. doi:10.1007/s00345-009-0428-7

791. Teber D, Sofikerim M, Ates M, et al. Is type 2 diabetes mellitus a predictive factor for incontinence after laparoscopic radical prostatectomy? A matched pair and multivariate analysis. J Urol. Mar 2010;183(3):1087-91. doi:10.1016/j.juro.2009.11.033

792. Terakawa T, Miyake H, Tanaka K, Takenaka A, Inoue TA, Fujisawa M. Surgical margin status of open versus laparoscopic radical prostatectomy specimens. Int J Urol. Aug 2008;15(8):704-7; discussion 708. doi:10.1111/j.1442-2042.2008.02057.x

793. Tewari AK, Patel ND, Leung RA, et al. Visual cues as a surrogate for tactile feedback during robotic-assisted laparoscopic prostatectomy: posterolateral margin rates in 1340 consecutive patients. BJU Int. Aug 2010;106(4):528-36. doi:10.1111/j.1464-410X.2009.09176.x

794. Tewari AK, Srivastava A, Huang MW, et al. Anatomical grades of nerve sparing: a risk-stratified approach to neural-hammock sparing during robot-assisted radical prostatectomy (RARP). BJU Int. Sep 2011;108(6 Pt 2):984-92. doi:10.1111/j.1464-410X.2011.10565.x

795. Tewari AK, Srivastava A, Mudaliar K, et al. Anatomical retro-apical technique of synchronous (posterior and anterior) urethral transection: a novel approach for ameliorating apical margin positivity during robotic radical prostatectomy. BJU Int. Nov 2010;106(9):1364-73. doi:10.1111/j.1464-410X.2010.09318.x

796. Tewari AK, Srivastava A, Sooriakumaran P, et al. Use of a novel absorbable barbed plastic surgical suture enables a "self-cinching" technique of vesicourethral anastomosis during robot-assisted prostatectomy and improves anastomotic times. J Endourol. Oct 2010;24(10):1645-50. doi:10.1089/end.2010.0316

797. Tewari A, Jhaveri J, Rao S, et al. Total reconstruction of the vesico-urethral junction. BJU Int. Apr 2008;101(7):871-7. doi:10.1111/j.1464-410X.2008.07424.x

798. Tewari A, Kaul S, Menon M. Robotic radical prostatectomy: a minimally invasive therapy for prostate cancer. Curr Urol Rep. Feb 2005;6(1):45-8. doi:10.1007/s11934-005-0066-6

799. Tewari A, Rao S, Mandhani A. Catheter-less robotic radical prostatectomy using a custom-made synchronous anastomotic splint and vesical urinary diversion device: report of the initial series and perioperative outcomes. BJU Int. Sep 2008;102(8):1000-4. doi:10.1111/j.1464-410X.2008.07875.x

800. Tewari A, Rao S, Martinez-Salamanca JI, et al. Cancer control and the preservation of neurovascular tissue: how to meet competing goals during robotic radical prostatectomy. BJU Int. Apr 2008;101(8):1013-8. doi:10.1111/j.1464-410X.2008.07456.x

801. Tewari A, Srivasatava A, Menon M, Team MotV. A prospective comparison of radical retropubic and robot-assisted prostatectomy: experience in one institution. BJU Int. Aug 2003;92(3):205-10. doi:10.1046/j.1464-410x.2003.04311.x

802. Tikuisis R, Miliauskas P, Samalavicius NE, Zurauskas A, Sruogis A. Epidural and general anesthesia versus general anesthesia in radical prostatectomy. Medicina (Kaunas). 2009;45(10):772-7.

803. Toren P, Alibhai SM, Matthew A, et al. The effect of nerve-sparing surgery on patient-reported continence post-radical prostatectomy. Can Urol Assoc J. Dec 2009;3(6):465-70. doi:10.5489/cuaj.1176

804. Touijer K, Eastham JA, Secin FP, et al. Comprehensive prospective comparative analysis of outcomes between open and laparoscopic radical prostatectomy conducted in 2003 to 2005. J Urol. May 2008;179(5):1811-7; discussion 1817. doi:10.1016/j.juro.2008.01.026

805. Touijer K, Kuroiwa K, Eastham JA, et al. Risk-adjusted analysis of positive surgical margins following laparoscopic and retropubic radical prostatectomy. Eur Urol. Oct 2007;52(4):1090-6. doi:10.1016/j.eururo.2006.12.014

806. Touijer K, Kuroiwa K, Vickers A, et al. Impact of a multidisciplinary continuous quality improvement program on the positive surgical margin rate after laparoscopic radical prostatectomy. Eur Urol. May 2006;49(5):853-8. doi:10.1016/j.eururo.2005.12.065

807. Touijer K, Secin FP, Cronin AM, et al. Oncologic outcome after laparoscopic radical prostatectomy: 10 years of experience. Eur Urol. May 2009;55(5):1014-9. doi:10.1016/j.eururo.2008.10.036

808. Tozawa K, Hashimoto Y, Yasui T, et al. Evaluation of operative complications related to laparoscopic radical prostatectomy. Int J Urol. Mar 2008;15(3):222-5. doi:10.1111/j.1442-2042.2007.01964.x

809. Tozawa K, Yasui T, Umemoto Y, et al. Pitfalls of robot-assisted radical prostatectomy: a comparison of positive surgical margins between robotic and laparoscopic surgery. Int J Urol. Oct 2014;21(10):976-9. doi:10.1111/iju.12492

810. Trabulsi EJ, Linden RA, Gomella LG, McGinnis DE, Strup SE, Lallas CD. The addition of robotic surgery to an established laparoscopic radical prostatectomy program: effect on positive surgical margins. Can J Urol. Apr 2008;15(2):3994-9.

811. Trabulsi EJ, Patel J, Viscusi ER, Gomella LG, Lallas CD. Preemptive multimodal pain regimen reduces opioid analgesia for patients undergoing robotic-assisted laparoscopic radical prostatectomy. Urology. Nov 2010;76(5):1122-4. doi:10.1016/j.urology.2010.03.052

812. Trabulsi EJ, Zola JC, Gomella LG, Lallas CD. Transition from pure laparoscopic to robotic-assisted radical prostatectomy: a single surgeon institutional evolution. Urol Oncol. 2010 Jan-Feb 2010;28(1):81-5. doi:10.1016/j.urolonc.2009.07.002

813. Travassos J, Figueiredo F, Xavier AO, De Juan K. Bilateral nerve-sparing extraperitoneal visual laser ablation radical prostatectomy: potency rates after 1 year follow up. J Endourol. Oct 2008;22(10):2327-32. doi:10.1089/end.2008.9720

814. Trinh QD, Sammon J, Sun M, et al. Perioperative outcomes of robot-assisted radical prostatectomy compared with open radical prostatectomy: results from the nationwide inpatient sample. Eur Urol. Apr 2012;61(4):679-85. doi:10.1016/j.eururo.2011.12.027

815. Truesdale MD, Lee DJ, Cheetham PJ, Hruby GW, Turk AT, Badani KK. Assessment of lymph node yield after pelvic lymph node dissection in men with prostate cancer: a comparison between robot-assisted radical prostatectomy and open radical prostatectomy in the modern era. J Endourol. Jul 2010;24(7):1055-60. doi:10.1089/end.2010.0128

816. Tsao AK, Smaldone MD, Averch TD, Jackman SV. Robot-assisted laparoscopic prostatectomy: the first 100 patients-improving patient safety and outcomes. J Endourol. Mar 2009;23(3):481-4. doi:10.1089/end.2008.0241

817. Tse E, Knaus R. Laparoscopic radical prostatectomy - results of 200 consecutive cases in a Canadian medical institution. Can J Urol. Apr 2004;11(2):2172-85.

818. Tseng TY, Kuebler HR, Cancel QV, et al. Prospective health-related quality-of-life assessment in an initial cohort of patients undergoing robotic radical prostatectomy. Urology. Nov 2006;68(5):1061-6. doi:10.1016/j.urology.2006.06.017

819. Tsivian A, Brodsky O, Shtricker A, Tsivian M, Benjamin S, Sidi AA. Urologic pelvic surgery following mesh hernia repair. Hernia. Oct 2009;13(5):523-7. doi:10.1007/s10029-009-0514-1

820. Türk I, Deger S, Winkelmann B, Schönberger B, Loening SA. Laparoscopic radical prostatectomy. Technical aspects and experience with 125 cases. Eur Urol. Jul 2001;40(1):46-52; discussion 53. doi:49748

821. Türkeri LN, Temiz Y, Yazici CM, Tinay I. A new suture technique for anastomosis in radical retropubic prostatectomy and early removal of urethral catheter. Can J Urol. Dec 2007;14(6):3734-8.

822. Twiss C, Slova D, Lepor H. Outcomes for men younger than 50 years undergoing radical prostatectomy. Urology. Jul 2005;66(1):141-6. doi:10.1016/j.urology.2005.01.049

823. Tzou DT, Dalkin BL, Christopher BA, Cui H. The failure of a nerve sparing template to improve urinary continence after radical prostatectomy: attention to study design. Urol Oncol. 2009 Jul-Aug 2009;27(4):358-62. doi:10.1016/j.urolonc.2008.01.013

824. Uberoi J, Brison D, Patel N, Sawczuk IS, Munver R. Robot-assisted laparoscopic radical prostatectomy in patients with prostate cancer with high-risk features: predictors of favorable pathologic outcome. J Endourol. Mar 2010;24(3):403-7. doi:10.1089/end.2009.0203

825. Uffort EE, Jensen JC. Impact of obesity on early erectile function recovery after robotic radical prostatectomy. JSLS. 2011 Jan-Mar 2011;15(1):32-7. doi:10.4293/108680810X12924466009203

826. Ukimura O, Aron M, Nakamoto M, et al. Three-dimensional surgical navigation model with TilePro display during robot-assisted radical prostatectomy. J Endourol. Jun 2014;28(6):625-30. doi:10.1089/end.2013.0749

827. Ukimura O, Magi-Galluzzi C, Gill IS. Real-time transrectal ultrasound guidance during laparoscopic radical prostatectomy: impact on surgical margins. J Urol. Apr 2006;175(4):1304-10. doi:10.1016/S0022-5347(05)00688-9

828. Uvin P, de Meyer JM, Van Holderbeke G. A comparison of the peri-operative data after open radical retropubic prostatectomy or robotic-assisted laparoscopic prostatectomy. Acta Chir Belg. 2010 May-Jun 2010;110(3):313-6. doi:10.1080/00015458.2010.11680623

829. Van Appledorn S, Bouchier-Hayes D, Agarwal D, Costello AJ. Robotic laparoscopic radical prostatectomy: setup and procedural techniques after 150 cases. Urology. Feb 2006;67(2):364-7. doi:10.1016/j.urology.2005.08.035

830. van der Poel HG, de Blok W. Role of extent of fascia preservation and erectile function after robot-assisted laparoscopic prostatectomy. Urology. Apr 2009;73(4):816-21. doi:10.1016/j.urology.2008.09.082

831. van der Poel HG, de Blok W, Bex A, Meinhardt W, Horenblas S. Peroperative transrectal ultrasonography-guided bladder neck dissection eases the learning of robot-assisted laparoscopic prostatectomy. BJU Int. Sep 2008;102(7):849-52. doi:10.1111/j.1464-410X.2008.07830.x

832. van der Poel HG, de Blok W, Joshi N, van Muilekom E. Preservation of lateral prostatic fascia is associated with urine continence after robotic-assisted prostatectomy. Eur Urol. Apr 2009;55(4):892-900. doi:10.1016/j.eururo.2009.01.021

833. van der Poel HG, de Blok W, Tillier C, van Muilekom E. Robot-assisted laparoscopic prostatectomy: nodal dissection results during the first 440 cases by two surgeons. J Endourol. Dec 2012;26(12):1618-24. doi:10.1089/end.2012.0360

834. van der Poel HG, Tillier C, de Blok W, van Muilekom E. Extended nodal dissection reduces sexual function recovery after robot-assisted laparoscopic prostatectomy. J Endourol. Sep 2012;26(9):1192-8. doi:10.1089/end.2012.0011

835. van Randenborgh H, Paul R, Kübler H, Breul J, Hartung R. Improved urinary continence after radical retropubic prostatectomy with preparation of a long, partially intraprostatic portion of the membraneous urethra: an analysis of 1013 consecutive cases. Prostate Cancer Prostatic Dis. 2004;7(3):253-7. doi:10.1038/sj.pcan.4500726

836. van Roermund JG, van Basten JP, Kiemeney LA, Karthaus HF, Witjes JA. Impact of obesity on surgical outcomes following open radical prostatectomy. Urol Int. 2009;82(3):256-61. doi:10.1159/000209353

837. Varkarakis J, Wirtenberger W, Pinggera GM, et al. Evaluation of urinary extravasation and results after continence-preserving radical retropubic prostatectomy. BJU Int. Nov 2004;94(7):991-5. doi:10.1111/j.1464-410X.2004.05092.x

838. Verze P, Greco F, Scuzzarella S, et al. The impact of previous prostate surgery on the outcomes of laparoscopic radical prostatectomy. Minerva Urol Nefrol. 02 2017;69(1):76-84. doi:10.23736/S0393-2249.16.02612-6

839. Vesey SG, McCabe JE, Hounsome L, Fowler S. UK radical prostatectomy outcomes and surgeon case volume: based on an analysis of the British Association of Urological Surgeons Complex Operations Database. BJU Int. Feb 2012;109(3):346-54. doi:10.1111/j.1464-410X.2011.10334.x

840. Vickers AJ, Bianco FJ, Serio AM, et al. The surgical learning curve for prostate cancer control after radical prostatectomy. J Natl Cancer Inst. Aug 2007;99(15):1171-7. doi:10.1093/jnci/djm060

841. Vickers AJ, Savage CJ, Hruza M, et al. The surgical learning curve for laparoscopic radical prostatectomy: a retrospective cohort study. Lancet Oncol. May 2009;10(5):475-80. doi:10.1016/S1470-2045(09)70079-8

842. Vickers A, Bianco F, Cronin A, et al. The learning curve for surgical margins after open radical prostatectomy: implications for margin status as an oncological end point. J Urol. Apr 2010;183(4):1360-5. doi:10.1016/j.juro.2009.12.015

843. Vijan SS, Wall JC, Greenlee SM, Farley DR. Consequences of endoscopic inguinal hernioplasty with mesh on subsequent open radical prostatectomy. Hernia. Aug 2008;12(4):415-9. doi:10.1007/s10029-008-0367-z

844. Viney R, Gommersall L, Zeif J, Hayne D, Shah ZH, Doherty A. Ultrasensitive prostate specific antigen assay following laparoscopic radical prostatectomy--an outcome measure for defining the learning curve. Ann R Coll Surg Engl. Jul 2009;91(5):399-403. doi:10.1308/003588409X428289

845. Vis AN, Schröder FH, van der Kwast TH. The actual value of the surgical margin status as a predictor of disease progression in men with early prostate cancer. Eur Urol. Aug 2006;50(2):258-65. doi:10.1016/j.eururo.2005.11.030

846. Vodopija N, Zupancic M, Korsic L, Kramer F, Parać I. Laparoscopic radical prostatectomy--analysis of our first 100 consecutive cases. Coll Antropol. Jun 2004;28(1):429-37.

847. Vora AA, Marchalik D, Kowalczyk KJ, et al. Robotic-assisted prostatectomy and open radical retropubic prostatectomy for locally-advanced prostate cancer: multi-institution comparison of oncologic outcomes. Prostate Int. 2013;1(1):31-6. doi:10.12954/PI.12001

848. Voss BL, Santiano K, Milano M, Mangold KA, Kaul KL. Integrity and amplification of nucleic acids from snap-frozen prostate tissues from robotic-assisted laparoscopic and open prostatectomies. Arch Pathol Lab Med. Apr 2013;137(4):525-30. doi:10.5858/arpa.2011-0550-OA

849. Wagner AA, Link RE, Trock BJ, Sullivan W, Pavlovich CP. Comparison of open and laparoscopic radical prostatectomy outcomes from a surgeon's early experience. Urology. Oct 2007;70(4):667-71. doi:10.1016/j.urology.2007.06.1104

850. Wagner A, Link R, Pavlovich C, Sullivan W, Su L. Use of a validated quality of life questionnaire to assess sexual function following laparoscopic radical prostatectomy. Int J Impot Res. 2006 Jan-Feb 2006;18(1):69-76. doi:10.1038/sj.ijir.3901376

851. Wallerstedt A, Tyritzis SI, Thorsteinsdottir T, et al. Short-term results after robot-assisted laparoscopic radical prostatectomy compared to open radical prostatectomy. Eur Urol. Apr 2015;67(4):660-70. doi:10.1016/j.eururo.2014.09.036

852. Walsh PC, Marschke P, Catalona WJ, et al. Efficacy of first-generation Cavermap to verify location and function of cavernous nerves during radical prostatectomy: a multi-institutional evaluation by experienced surgeons. Urology. Mar 2001;57(3):491-4. doi:10.1016/s0090-4295(00)01067-0

853. Walsh PC, Marschke P, Ricker D, Burnett AL. Patient-reported urinary continence and sexual function after anatomic radical prostatectomy. Urology. Jan 2000;55(1):58-61. doi:10.1016/s0090-4295(99)00397-0

854. Wambi CO, Siddiqui SA, Krane LS, Agarwal PK, Stricker HJ, Peabody JO. Early oncological outcomes of robot-assisted radical prostatectomy for high-grade prostate cancer. BJU Int. Dec 2010;106(11):1739-45. doi:10.1111/j.1464-410X.2010.09484.x

855. Wang L, Chung SF, Yip SK, Lau WK, Cheng CW, Sim HG. The natural history of voiding function after robot-assisted laparoscopic radical prostatectomy. Urol Oncol. 2011 Mar-Apr 2011;29(2):177-82. doi:10.1016/j.urolonc.2009.01.030

856. Wang R, Wood DP, Hollenbeck BK, et al. Risk factors and quality of life for post-prostatectomy vesicourethral anastomotic stenoses. Urology. Feb 2012;79(2):449-57. doi:10.1016/j.urology.2011.07.1383

857. Ward JF, Zincke H, Bergstralh EJ, Slezak JM, Myers RP, Blute ML. The impact of surgical approach (nerve bundle preservation versus wide local excision) on surgical margins and biochemical recurrence following radical prostatectomy. J Urol. Oct 2004;172(4 Pt 1):1328-32. doi:10.1097/01.ju.0000138681.64035.dc

858. Watts R, Botti M, Beale E, Crowe H, Costello AJ. Patient outcomes in the acute recovery phase following robotic-assisted prostate surgery: a prospective study. Int J Nurs Stud. Apr 2009;46(4):442-9. doi:10.1016/j.ijnurstu.2007.07.010

859. Webb DR, Sethi K, Gee K. An analysis of the causes of bladder neck contracture after open and robot-assisted laparoscopic radical prostatectomy. BJU Int. Apr 2009;103(7):957-63. doi:10.1111/j.1464-410X.2008.08278.x

860. Weizer AZ, Strope S, Wood DP. Margin control in robotic and laparoscopic prostatectomy: what are the REAL outcomes? Urol Oncol. 2010 Mar-Apr 2010;28(2):210-4. doi:10.1016/j.urolonc.2009.08.009

861. White MA, De Haan AP, Stephens DD, Maatman TK, Maatman TJ. Comparative analysis of surgical margins between radical retropubic prostatectomy and RALP: are patients sacrificed during initiation of robotics program? Urology. Mar 2009;73(3):567-71. doi:10.1016/j.urology.2008.11.011

862. Wiatr T, Golabek T, Dudek P, et al. Single Running Suture versus Single-Knot Running Suture for Vesicourethral Anastomosis in Laparoscopic Radical Prostatectomy: A Prospective Randomised Comparative Study. Urol Int. 2015;95(4):445-51. doi:10.1159/000438829

863. Williams SB, Alemozaffar M, Lei Y, et al. Randomized controlled trial of barbed polyglyconate versus polyglactin suture for robot-assisted laparoscopic prostatectomy anastomosis: technique and outcomes. Eur Urol. Dec 2010;58(6):875-81. doi:10.1016/j.eururo.2010.07.021

864. Williams SB, Chen MH, D'Amico AV, et al. Radical retropubic prostatectomy and robotic-assisted laparoscopic prostatectomy: likelihood of positive surgical margin(s). Urology. Nov 2010;76(5):1097-101. doi:10.1016/j.urology.2009.11.079

865. Williams SB, D'Amico AV, Weinberg AC, Gu X, Lipsitz SR, Hu JC. Population-based determinants of radical prostatectomy surgical margin positivity. BJU Int. Jun 2011;107(11):1734-40. doi:10.1111/j.1464-410X.2010.09662.x

866. Williams SB, Prasad SM, Weinberg AC, et al. Trends in the care of radical prostatectomy in the United States from 2003 to 2006. BJU Int. Jul 2011;108(1):49-55. doi:10.1111/j.1464-410X.2010.09822.x

867. Williams SB, Sutherland DE, Frazier HA, Schwartz A, Engel JD. Pathologic analysis of capsular and incisional denudation and positive margin status in the development of a robot-assisted laparoscopic prostatectomy program. J Robot Surg. Oct 2009;3(3):137. doi:10.1007/s11701-009-0148-4

868. Willis DL, Gonzalgo ML, Brotzman M, Feng Z, Trock B, Su LM. Comparison of outcomes between pure laparoscopic vs robot-assisted laparoscopic radical prostatectomy: a study of comparative effectiveness based upon validated quality of life outcomes. BJU Int. Mar 2012;109(6):898-905. doi:10.1111/j.1464-410X.2011.10551.x

869. Wilson LC, Kennett KM, Gilling PJ. Laparoscopic radical prostatectomy: early safety and efficacy. ANZ J Surg. Dec 2004;74(12):1065-8. doi:10.1111/j.1445-1433.2004.03269.x

870. Wilson LC, Pickford JE, Gilling PJ. Robot-assisted laparoscopic radical prostatectomy (RALP)--a new surgical treatment for cancer of the prostate. N Z Med J. Dec 2008;121(1287):32-8.

871. Wiltz AL, Shikanov S, Eggener SE, et al. Robotic radical prostatectomy in overweight and obese patients: oncological and validated-functional outcomes. Urology. Feb 2009;73(2):316-22. doi:10.1016/j.urology.2008.08.493

872. Wirth GJ, Psutka SP, Chapin BF, Wu S, Wu CL, Dahl DM. Midterm oncological outcomes of laparoscopic vs open radical prostatectomy (RP). BJU Int. Jul 2013;112(2):190-7. doi:10.1111/bju.12085

873. Wolanski P, Chabert C, Jones L, Mullavey T, Walsh S, Gianduzzo T. Preliminary results of robot-assisted laparoscopic radical prostatectomy (RALP) after fellowship training and experience in laparoscopic radical prostatectomy (LRP). BJU Int. Dec 2012;110 Suppl 4:64-70. doi:10.1111/j.1464-410X.2012.11479.x

874. Wolfram M, Bräutigam R, Engl T, et al. Robotic-assisted laparoscopic radical prostatectomy: the Frankfurt technique. World J Urol. Aug 2003;21(3):128-32. doi:10.1007/s00345-003-0346-z

875. Wong RP, Carter HB, Wolfson A, Faustin C, Cohen SR, Wu CL. Use of spinal anesthesia does not reduce intraoperative blood loss. Urology. Sep 2007;70(3):523-6. doi:10.1016/j.urology.2007.04.034

876. Woo JR, Shikanov S, Zorn KC, Shalhav AL, Zagaja GP. Impact of posterior rhabdosphincter reconstruction during robot-assisted radical prostatectomy: retrospective analysis of time to continence. J Endourol. Dec 2009;23(12):1995-9. doi:10.1089/end.2009.0117

877. Wood DP, Schulte R, Dunn RL, et al. Short-term health outcome differences between robotic and conventional radical prostatectomy. Urology. Nov 2007;70(5):945-9. doi:10.1016/j.urology.2007.06.1120

878. Wu SD, Meeks JJ, Cashy J, Perry KT, Nadler RB. Suture versus staple ligation of the dorsal venous complex during robot-assisted laparoscopic radical prostatectomy. BJU Int. Aug 2010;106(3):385-90. doi:10.1111/j.1464-410X.2009.09146.x

879. Wuethrich PY, Hsu Schmitz SF, Kessler TM, et al. Potential influence of the anesthetic technique used during open radical prostatectomy on prostate cancer-related outcome: a retrospective study. Anesthesiology. Sep 2010;113(3):570-6. doi:10.1097/ALN.0b013e3181e4f6ec

880. Wyler SF, Ruszat R, Straumann U, et al. Short-, intermediate-, and long-term quality of life after laparoscopic radical prostatectomy--does the learning curve of LRP have a negative impact on patients' quality of life? Eur Urol. Apr 2007;51(4):1004-12; discussion 1012-4. doi:10.1016/j.eururo.2006.10.065

881. Yamada Y, Fujimura T, Fukuhara H, et al. Incidence and risk factors of inguinal hernia after robot-assisted radical prostatectomy. World J Surg Oncol. Mar 2017;15(1):61. doi:10.1186/s12957-017-1126-3

882. Yang C-J, Ou Y-C, Yang C-K. Percutaneous cystostomy drainage for early removing urethral catheter in robotic-assisted laparoscopic radical prostatectomy: Improving on patients' discomfort. Urological Science. 2015/12/01/ 2015;26(4):240-242. doi:https://doi.org/10.1016/j.urols.2015.01.004

883. Yang J, Shao PF, Lv Q, et al. Continuous suture of a single absorbable suture: a new simplified vesicourethral anastomosis technique in laparoscopic radical prostatectomy. Int Surg. 2014 Sep-Oct 2014;99(5):656-61. doi:10.9738/INTSURG-D-13-00124.1

884. Yang Y, Luo Y, Hou GL, et al. Laparoscopic Radical Prostatectomy after Previous Transurethral Resection of the Prostate in Clinical T1a and T1b Prostate Cancer: A Matched-Pair Analysis. Urol J. Jul 2015;12(3):2154-9.

885. Yao SL, Lu-Yao G. Population-based study of relationships between hospital volume of prostatectomies, patient outcomes, and length of hospital stay. J Natl Cancer Inst. Nov 1999;91(22):1950-6. doi:10.1093/jnci/91.22.1950

886. Yaxley JW, Coughlin GD, Chambers SK, et al. Robot-assisted laparoscopic prostatectomy versus open radical retropubic prostatectomy: early outcomes from a randomised controlled phase 3 study. Lancet. 09 2016;388(10049):1057-1066. doi:10.1016/S0140-6736(16)30592-X

887. Yazici S, Inci K, Yuksel S, Bilen CY, Ozen H. Radical prostatectomy after previous prostate surgery: effects on surgical difficulty and pathologic outcomes. Urology. Apr 2009;73(4):856-9. doi:10.1016/j.urology.2008.09.024

888. Yee DS, Narula N, Amin MB, Skarecky DW, Ahlering TE. Robot-assisted radical prostatectomy: current evaluation of surgical margins in clinically low-, intermediate-, and high-risk prostate cancer. J Endourol. Sep 2009;23(9):1461-5. doi:10.1089/end.2009.0144

889. Yonekura H, Hirate H, Sobue K. Comparison of anesthetic management and outcomes of robot-assisted vs pure laparoscopic radical prostatectomy. J Clin Anesth. Dec 2016;35:281-286. doi:10.1016/j.jclinane.2016.08.014

890. Yoshimine S, Miyajima A, Nakagawa K, Ide H, Kikuchi E, Oya M. Extraperitoneal approach induces postoperative inguinal hernia compared with transperitoneal approach after laparoscopic radical prostatectomy. Jpn J Clin Oncol. Apr 2010;40(4):349-52. doi:10.1093/jjco/hyp172

891. You YC, Kim TH, Sung GT. Effect of Bladder Neck Preservation and Posterior Urethral Reconstruction during Robot-Assisted Laparoscopic Radical Prostatectomy for Urinary Continence. Korean J Urol. Jan 2012;53(1):29-33. doi:10.4111/kju.2012.53.1.29

892. Yu HY, Hevelone ND, Lipsitz SR, Kowalczyk KJ, Hu JC. Use, costs and comparative effectiveness of robotic assisted, laparoscopic and open urological surgery. J Urol. Apr 2012;187(4):1392-8. doi:10.1016/j.juro.2011.11.089

893. Yu HY, Hevelone ND, Lipsitz SR, Kowalczyk KJ, Nguyen PL, Hu JC. Hospital volume, utilization, costs and outcomes of robot-assisted laparoscopic radical prostatectomy. J Urol. May 2012;187(5):1632-7. doi:10.1016/j.juro.2011.12.071

894. Yuh BE, Ruel NH, Mejia R, Novara G, Wilson TG. Standardized comparison of robot-assisted limited and extended pelvic lymphadenectomy for prostate cancer. BJU Int. Jul 2013;112(1):81-8. doi:10.1111/j.1464-410X.2012.11788.x

895. Zarrelli G, Mastroprimiano G, Giovannone R, De Berardinis E, Gentile V, Busetto GM. Knotless "three-U-stitches" technique for urethrovesical anastomosis during laparoscopic radical prostatectomy. Int J Urol. Apr 2013;20(4):441-4. doi:10.1111/j.1442-2042.2012.03153.x

896. Zheng T, Zhang X, Ma X, et al. A matched-pair comparison between bilateral intrafascial and interfascial nerve-sparing techniques in extraperitoneal laparoscopic radical prostatectomy. Asian J Androl. Jul 2013;15(4):513-7. doi:10.1038/aja.2012.157

897. Zilberman DE, Tsivian M, Yong D, Albala DM. Surgical steps that elongate operative time in robot-assisted radical prostatectomy among the obese population. J Endourol. May 2011;25(5):793-6. doi:10.1089/end.2010.0428

898. Zilberman DE, Tsivian M, Yong D, Ferrandino MN, Albala DM. Does body mass index have an impact on the rate and location of positive surgical margins following robot assisted radical prostatectomy? Urol Oncol. 2012 Nov-Dec 2012;30(6):790-3. doi:10.1016/j.urolonc.2010.10.004

899. Zincke H, Bergstralh EJ, Blute ML, et al. Radical prostatectomy for clinically localized prostate cancer: long-term results of 1,143 patients from a single institution. J Clin Oncol. Nov 1994;12(11):2254-63. doi:10.1200/JCO.1994.12.11.2254

900. Zincke H, Oesterling JE, Blute ML, Bergstralh EJ, Myers RP, Barrett DM. Long-term (15 years) results after radical prostatectomy for clinically localized (stage T2c or lower) prostate cancer. J Urol. Nov 1994;152(5 Pt 2):1850-7. doi:10.1016/s0022-5347(17)32399-6

901. Zorn KC, Gofrit ON, Orvieto MA, et al. Da Vinci robot error and failure rates: single institution experience on a single three-arm robot unit of more than 700 consecutive robot-assisted laparoscopic radical prostatectomies. J Endourol. Nov 2007;21(11):1341-4. doi:10.1089/end.2006.0455

902. Zorn KC, Gofrit ON, Orvieto MA, Mikhail AA, Zagaja GP, Shalhav AL. Robotic-assisted laparoscopic prostatectomy: functional and pathologic outcomes with interfascial nerve preservation. Eur Urol. Mar 2007;51(3):755-62; discussion 763. doi:10.1016/j.eururo.2006.10.019

903. Zorn KC, Gofrit ON, Steinberg GP, Taxy JB, Zagaja GP, Shalhav AL. Planned nerve preservation to reduce positive surgical margins during robot-assisted laparoscopic radical prostatectomy. J Endourol. Jun 2008;22(6):1303-9. doi:10.1089/end.2008.0009

904. Zorn KC, Katz MH, Bernstein A, et al. Pelvic lymphadenectomy during robot-assisted radical prostatectomy: Assessing nodal yield, perioperative outcomes, and complications. Urology. Aug 2009;74(2):296-302. doi:10.1016/j.urology.2009.01.077

905. Zorn KC, Orvieto MA, Gong EM, et al. Robotic radical prostatectomy learning curve of a fellowship-trained laparoscopic surgeon. J Endourol. Apr 2007;21(4):441-7. doi:10.1089/end.2006.0239

906. Zorn KC, Orvieto MA, Mikhail AA, et al. Effect of prostate weight on operative and postoperative outcomes of robotic-assisted laparoscopic prostatectomy. Urology. Feb 2007;69(2):300-5. doi:10.1016/j.urology.2006.10.021

907. Zorn KC, Trinh QD, Jeldres C, et al. Prospective randomized trial of barbed polyglyconate suture to facilitate vesico-urethral anastomosis during robot-assisted radical prostatectomy: time reduction and cost benefit. BJU Int. May 2012;109(10):1526-32. doi:10.1111/j.1464-410X.2011.10763.x

908. Zorn KC, Wille MA, Thong AE, et al. Continued improvement of perioperative, pathological and continence outcomes during 700 robot-assisted radical prostatectomies. Can J Urol. Aug 2009;16(4):4742-9; discussion 4749.

909. Zugor V, Labanaris AP, Porres D, Witt JH. Surgical, oncologic, and short-term functional outcomes in patients undergoing robot-assisted prostatectomy after previous transurethral resection of the prostate. J Endourol. May 2012;26(5):515-9. doi:10.1089/end.2011.0205

910. Zugor V, Witt JH, Heidenreich A, Porres D, Labanaris AP. Surgical and oncological outcomes in patients with preoperative PSA >20 ng/ml undergoing robot-assisted radical prostatectomy. Anticancer Res. May 2012;32(5):2091-5.
